# Supplementary material for: Extensive binding of poorly characterized human transcription factors to genomic dark matter
Source: Nat Commun. 2026 Aug 5;17:7770. doi: 10.1038/s41467-026-75376-z (PMC13444102; doi:10.1038/s41467-026-75376-z)
Supplement: Supplementary file 1 [file 41467_2026_75376_MOESM1_ESM.pdf]

Supplementary Information

Extensive binding of poorly characterized human transcription factors to genomic dark matter

Rozita Razavi[1,#], Ali Fathi[1,#], Isaac Yellan[1,#], Alexander Brechalov[1,#], Kaitlin U. Laverty[1,2], Arttu Jolma[1], Aldo Hernandez-Corchado[3], Hong Zheng[1], Ally W.H. Yang[1], Mihai Albu[1], Marjan Barazandeh[1], Chun Hu[1], Ilya E. Vorontsov[4], Zain M. Patel[1], The Codebook Consortium*, Ivan V. Kulakovskiy[4,5,6], Philipp Bucher[7], Quaid D. Morris[2], Hamed S. Najafabadi[3,8], and Timothy R. Hughes[1]**

[1]Donnelly Centre and Department of Molecular Genetics, 160 College Street, Toronto, ON M5S 3E1, Canada
[2]Sloan Kettering Institute, Memorial Sloan Kettering Cancer Center, New York, NY 25 10065, USA
[3]Victor P. Dahdaleh Institute of Genomic Medicine, Montréal, QC H3A 0G1, Canada
[4]Vavilov Institute of General Genetics, Russian Academy of Sciences, 119991, Moscow, Russia
[5]Institute of Protein Research, Russian Academy of Sciences, 142290, Pushchino, Russia
[6]Institute of Biochemistry and Genetics, Ufa Federal Research Centre of Russian Academy of Sciences, 450054, Ufa, Russia
[7]Swiss Institute of Bioinformatics, 1015, Lausanne, Switzerland
[8]Department of Human Genetics, McGill University, Montréal, QC H3A 0C7, Canada
[#]These authors contributed equally: Rozita Razavi, Ali Fathi, Isaac Yellan, Alexander Brechalov

* A list of authors and their affiliations appears at the end of the paper.
** To whom correspondence should be addressed: t.hughes@utoronto.ca

Table of Contents:

Supplementary Figures

Supplementary Fig. 1 3
Supplementary Fig. 2 5
Supplementary Fig. 3 7
Supplementary Fig. 4 9
Supplementary Fig. 5 11
Supplementary Fig. 6 13
Supplementary Fig. 7 15
Supplementary Fig. 8 17
Supplementary Fig. 9 19
Supplementary Fig. 10 21
Supplementary Fig. 11 23
Supplementary Fig. 12 25

Supplementary Note 1. Overview of Dark TFs' function. This document provides a summary of the functional impact of Dark TFs. It is based on a survey (**Fig. 7**) of Dark TF interactions with chromatin remodelers, other zinc-finger proteins, and the specific categories and ages of enriched TEs within each TF's TOPs, their motifs, as well as documented functions based on a literature review. 27

Supplementary Note 2. Analysis of binding site ages for TFs. This document provides a summary of the evolutionary analyses performed to estimate the age of each TOP site for all TFs in this study, as well as the ages of the TFs. 29

Supplementary References 30

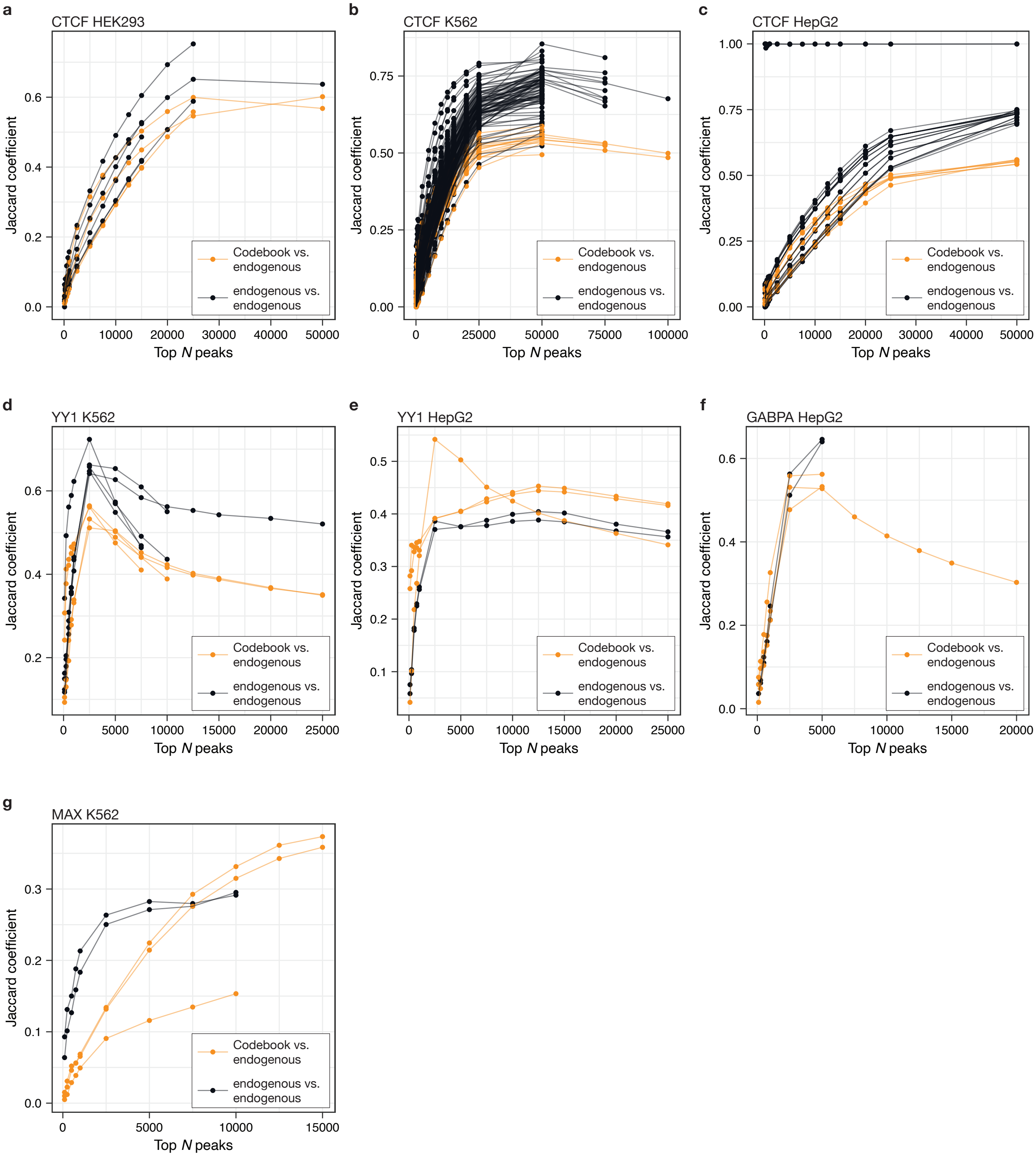
a
CTCF HEK293
Jaccard coefficient
Top N peaks
Codebook vs. endogenous
endogenous vs. endogenous
b
CTCF K562
Jaccard coefficient
Top N peaks
Codebook vs. endogenous
endogenous vs. endogenous
c
CTCF HepG2
Jaccard coefficient
Top N peaks
Codebook vs. endogenous
endogenous vs. endogenous
d
YY1 K562
Jaccard coefficient
Top N peaks
Codebook vs. endogenous
endogenous vs. endogenous
e
YY1 HepG2
Jaccard coefficient
Top N peaks
Codebook vs. endogenous
endogenous vs. endogenous
f
GABPA HepG2
Jaccard coefficient
Top N peaks
Codebook vs. endogenous
endogenous vs. endogenous
g
MAX K562
Jaccard coefficient
Top N peaks
Codebook vs. endogenous
endogenous vs. endogenous

Supplementary Fig. 1. Comparison of ChIP-seq peaks obtained from ectopically expressed TF in Codebook project with ChIP-seq peaks from previous studies using endogenous TFs. Individual plots show the overlap (Jaccard coefficient) between peak sets for pairs of ChIP-seq experiments. ChIP-seq peaks are ordered by their scores and the Jaccard coefficient is calculated using the top *N* peaks from each experiment, with different values of *N*. Comparisons between pairs of ChIP-seq experiments that immunoprecipitated the endogenous TF (endogenous vs. endogenous) are distinguished from comparisons between a Codebook ChIP-seq experiment and a ChIP-seq experiment that immunoprecipitated the endogenous TF (Codebook vs endogenous). All Codebook ChIP-seq experiments are performed in HEK293 cells, each panel shows comparisons for a specific TF with endogenous immunoprecipitated experiments performed in a specific cell line. (**a**), CTCF, HEK293 cells. (**b**) CTCF, K562 cells. (**c)** CTCF, HepG2 cells. (**d**) YY1, K562 cells. (**e**) YY1, HepG2 cells. (**f**) GABPA, HepG2 cells. (**g**) MAX, K572 cells. Source data are provided as a Source Data file. TF: transcription factor.

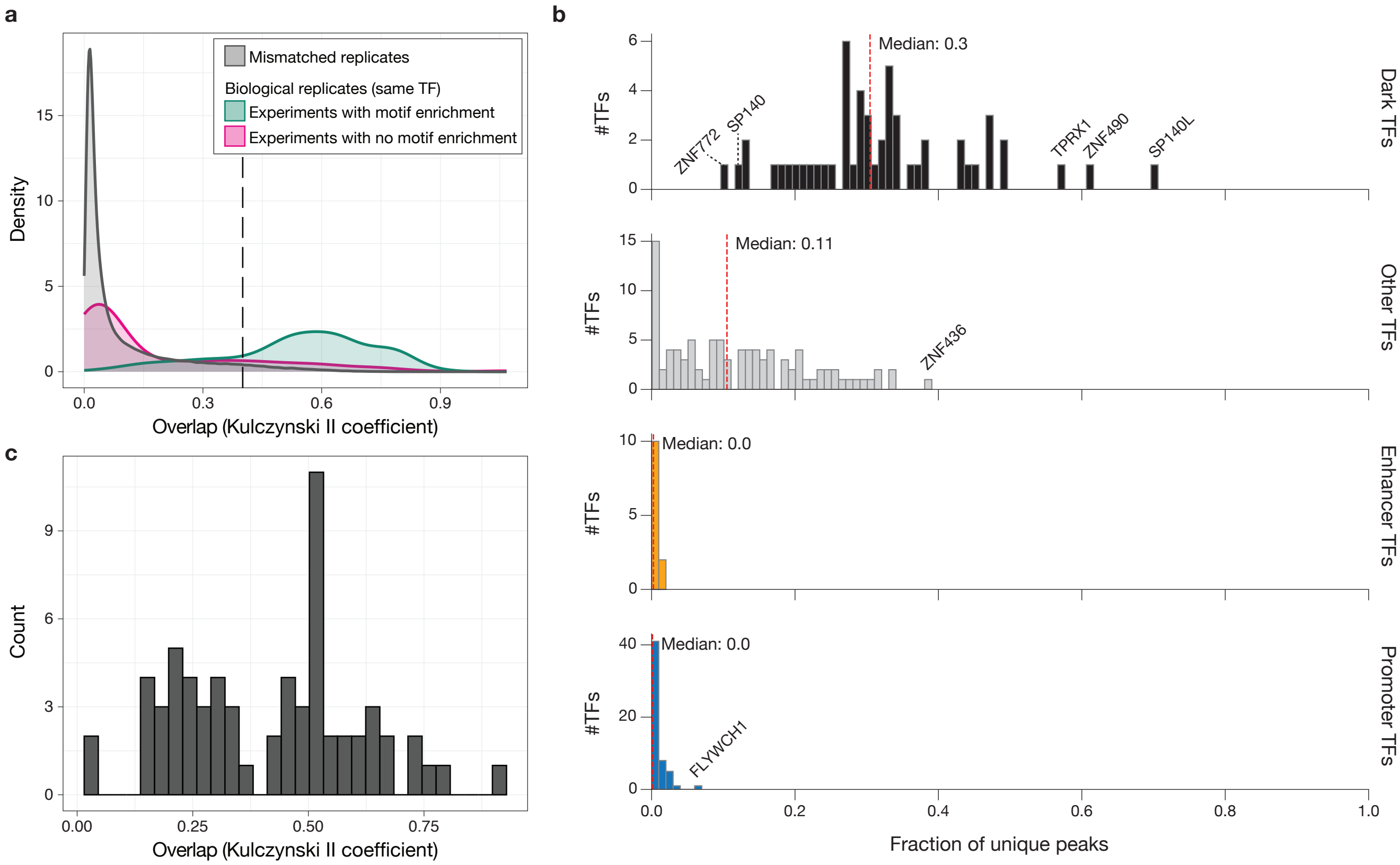
a
Mismatched replicates
Biological replicates (same TF)
Experiments with motif enrichment
Experiments with no motif enrichment
Density
Overlap (Kulczynski II coefficient)
b
Median: 0.3
ZNF772
SP140
TPRX1
ZNF490
SP140L
Dark TFs
Median: 0.11
ZNF436
Other TFs
Median: 0.0
Enhancer TFs
Median: 0.0
FLYWCH1
Promoter TFs
#TFs
Fraction of unique peaks
c
Count
Overlap (Kulczynski II coefficient)

Supplementary Fig. 2. Peak overlap assessment of ChIP-seq as a measure of success. (**a**) Distribution of peak overlap between ChIP-seq replicates, for the experiments yielded a motif (green), experiments that did not yield a motif (red), and the mismatched (i.e., TF identities permuted) replicates (grey). Values are calculated Kulczynski II similarity metric (i.e. average of overlaps). Dotted line indicates the threshold at which the peak overlap of experimental replicates without motif enrichment considered successful and were included in downstream analyses. (**b**) Distribution peak uniqueness for defined TF categories (Promoter, Enhancer, Dark, and Other), measured as the fraction of ChIP-seq peaks (at the universal threshold) not overlapping with any peak from any other TF in this study. (**c**) Distribution of Kulczynski II similarity metric between successful ChIP-seq replicates (as in (**a**)), restricted to the TFs that have a low peak overlap with other TFs (specifically, the 87 TFs in the upper left darker region of the square matrix in **Fig. 2b**). Source data are provided as a Source Data file. TF: transcription factor.

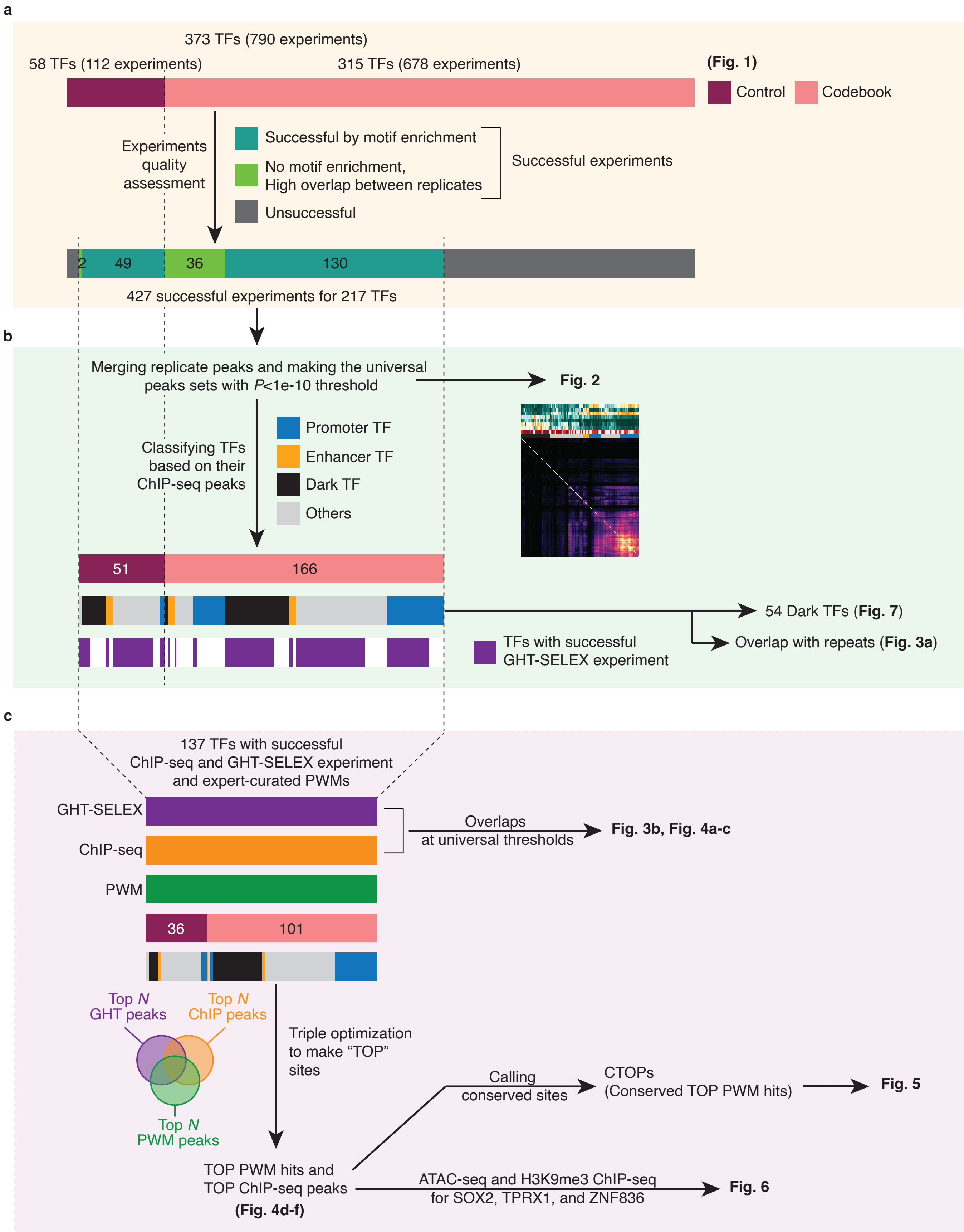
a
373 TFs (790 experiments)
58 TFs (112 experiments)
315 TFs (678 experiments)
(Fig. 1)
Control
Codebook
Experiments quality assessment
Successful by motif enrichment
No motif enrichment, High overlap between replicates
Unsuccessful
Successful experiments
2
49
36
130
427 successful experiments for 217 TFs
b
Merging replicate peaks and making the universal peaks sets with P<1e-10 threshold
Fig. 2
Classifying TFs based on their ChIP-seq peaks
Promoter TF
Enhancer TF
Dark TF
Others
51
166
54 Dark TFs (Fig. 7)
Overlap with repeats (Fig. 3a)
TFs with successful GHT-SELEX experiment
c
137 TFs with successful ChIP-seq and GHT-SELEX experiment and expert-curated PWMs
GHT-SELEX
ChIP-seq
PWM
Overlaps at universal thresholds
Fig. 3b, Fig. 4a-c
36
101
Top N GHT peaks
Top N ChIP peaks
Top N PWM peaks
Triple optimization to make "TOP" sites
Calling conserved sites
CTOPs (Conserved TOP PWM hits)
Fig. 5
TOP PWM hits and TOP ChIP-seq peaks
(Fig. 4d-f)
ATAC-seq and H3K9me3 ChIP-seq for SOX2, TPRX1, and ZNF836
Fig. 6

Supplementary Fig. 3. Overview of ChP-seq data processing. (**a**) selection process for successful ChIP-seq experiments based on replicate overlap and motif enrichment. Data for 790 ChIP-seq experiments (including replicates for 373 TFs) were assessed and 427 (217 TFs) were considered successful. (**b**) peaks for the successful replicates were merged to create one set of peaks for each TF. The overlap of peak sets with each other and various genomic tracks were evaluated, and four binding categories of TFs emerged. 137 of these TFs also had successful GHT-SELEX experiments as labelled in purple. (**c**) defining triple-optimized overlap peaks (TOPs) and conserved TOPs (CTOPs). TOPs were defined based on overlap of ChIP-seq and GHT-SELEX peaks as well as PWM predicted binding sites at optimized thresholds (see **Methods**). CTOPs were defied based on phyloP conservation pattern of the PWM hit. TF: transcription factor, PWM: Position weight matrix, TOP: triple-optimized overlap, CTOP: conserved triple-optimized overlap.

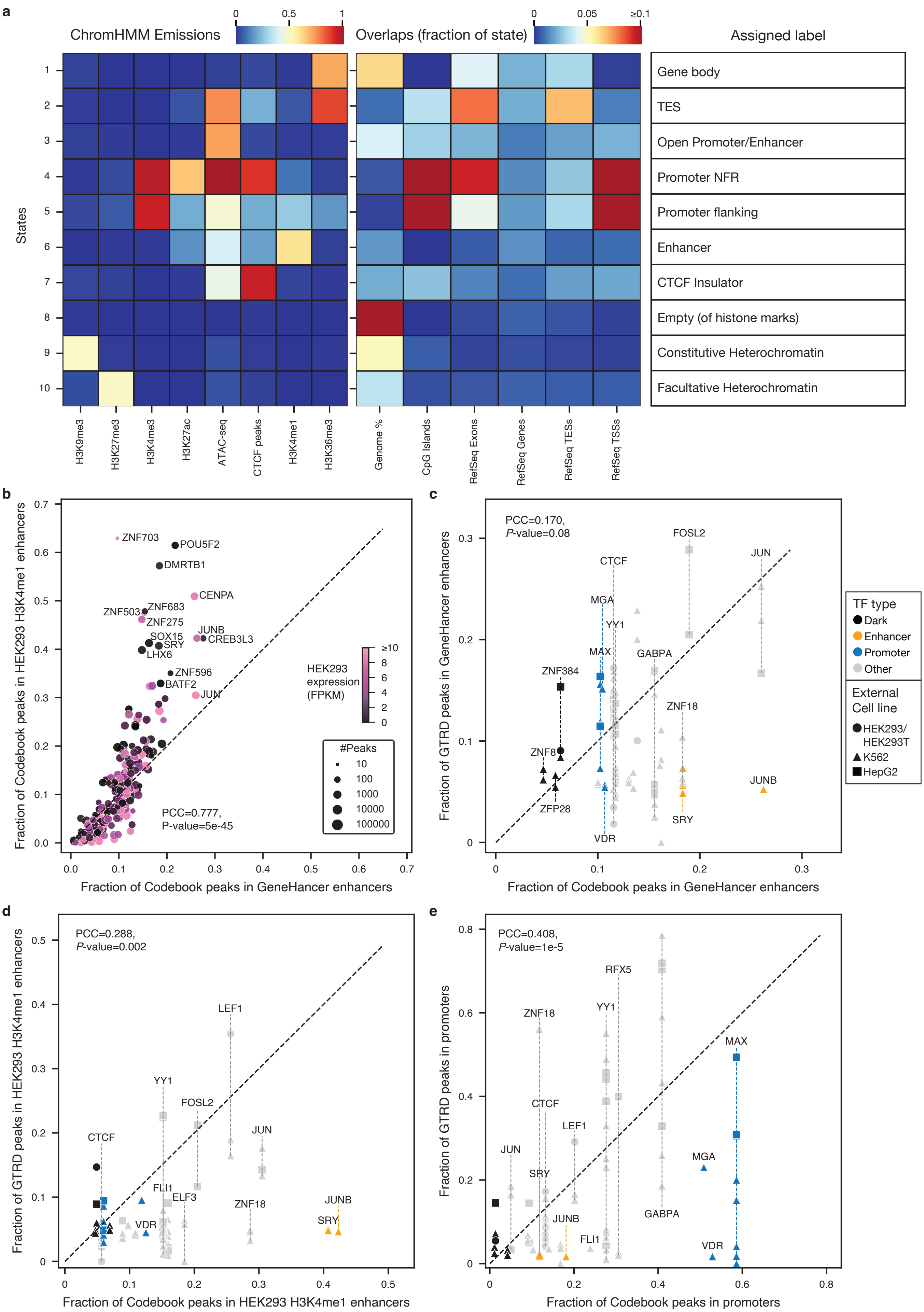
a
ChromHMM Emissions
0
0.5
1
Overlaps (fraction of state)
0
0.05
≥0.1
Assigned label
States
1
2
3
4
5
6
7
8
9
10
Gene body
TES
Open Promoter/Enhancer
Promoter NFR
Promoter flanking
Enhancer
CTCF Insulator
Empty (of histone marks)
Constitutive Heterochromatin
Facultative Heterochromatin
H3K9me3
H3K27me3
H3K4me3
H3K27ac
ATAC-seq
CTCF peaks
H3K4me1
H3K36me3
Genome %
CpG Islands
RefSeq Exons
RefSeq Genes
RefSeq TESs
RefSeq TSSs
b
Fraction of Codebook peaks in HEK293 H3K4me1 enhancers
Fraction of Codebook peaks in GeneHancer enhancers
ZNF703
POU5F2
DMRTB1
CENPA
ZNF503
ZNF683
ZNF275
JUNB
SOX15
CREB3L3
SRY
LHX6
ZNF596
BATF2
JUN
HEK293 expression (FPKM)
≥10
8
6
4
2
0
#Peaks
10
100
1000
10000
100000
PCC=0.777,
P-value=5e-45
c
PCC=0.170,
P-value=0.08
Fraction of GTRD peaks in GeneHancer enhancers
Fraction of Codebook peaks in GeneHancer enhancers
FOSL2
CTCF
JUN
MGA
YY1
MAX
GABPA
ZNF384
ZNF18
ZNF8
ZFP28
VDR
SRY
JUNB
TF type
Dark
Enhancer
Promoter
Other
External Cell line
HEK293/ HEK293T
K562
HepG2
d
PCC=0.288,
P-value=0.002
Fraction of GTRD peaks in HEK293 H3K4me1 enhancers
Fraction of Codebook peaks in HEK293 H3K4me1 enhancers
LEF1
YY1
FOSL2
CTCF
JUN
FLI1
ELF3
ZNF18
JUNB
VDR
SRY
e
PCC=0.408,
P-value=1e-5
Fraction of GTRD peaks in promoters
Fraction of Codebook peaks in promoters
RFX5
ZNF18
YY1
MAX
CTCF
LEF1
JUN
SRY
MGA
JUNB
FLI1
GABPA
VDR

Supplementary Fig. 4. Generating HEK293-specific chromatin states. (**a**) Characterization of output tracks for the ChromHMM with 10 states trained on various HEK293-specific ChIP-seq and ATAC-seq data (i.e., ChIP-seq data for H3K9me3, H3K27me3, H3K4me1, H3K4me3, H3K36me3, and H3K27ac from ENCODE project, and ATAC-seq and CTCF peaks generated here by Codebook project). Based on the correspondence between emissions and the chromatin marks and genome annotations, states were assigned to: Gene body, Transcription end site (TES), Open Promoter/Enhancer, Promoter NFR (nucleosome-free regions), Promoter flanking region, Enhancer, CTCF Insulator, Empty (of assigned histone marks), Constitutive Heterochromatin, and Facultative Heterochromatin. (**b**) the panel shows the fraction of TF ChIP-seq peaks overlap with GeneHancer annotated enhancers (x-axis) and HEK293 enhancers (defined by H3K4me1-positive regions from ChromHMM; y-axis). Circle sizes (TFs) are scaled based on peak numbers and color gradient corresponds to endogenous expression level of TFs in HEK293 cells[1]. (**c, d, e**) Comparison of Codebook ChIP-seq peaks and publicly available ChIP-seq or ChIP-exo data on GTRD, for 21 control TFs, based on the fraction of peaks overlapping with GeneHancer enhancers (**c**), HEK293 defined enhancers (**d**), and protein-coding promoters (**e**). For the Codebook data (x-axis), the merged peak set with the universal threshold has been used. Pearson correlation coefficient (PCC) and the *P*-value for the Pearson correlation test are also provided for each panel. Source data are provided as a Source Data file. TF: transcription factor, PCC: Pearson correlation coefficient, FPKM: Fragments Per Kilobase of transcript per Million mapped reads.

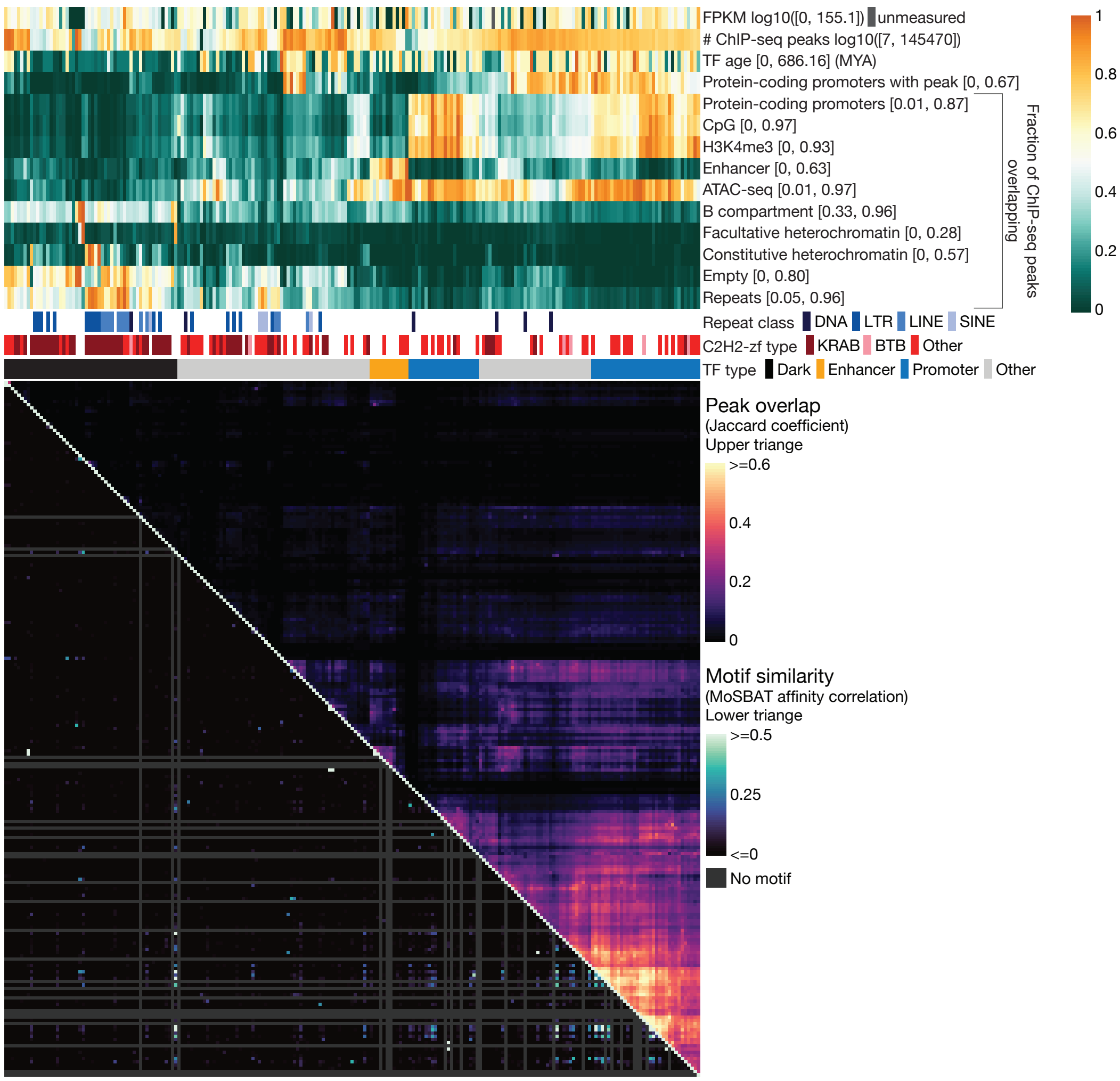

FPKM log10([0, 155.1]) unmeasured
ChIP-seq peaks log10([7, 145470])
TF age [0, 686.16] (MYA)
Protein-coding promoters with peak [0, 0.67]
Protein-coding promoters [0.01, 0.87]
CpG [0, 0.97]
H3K4me3 [0, 0.93]
Enhancer [0, 0.63]
ATAC-seq [0.01, 0.97]
B compartment [0.33, 0.96]
Facultative heterochromatin [0, 0.28]
Constitutive heterochromatin [0, 0.57]
Empty [0, 0.80]
Repeats [0.05, 0.96]
Fraction of ChIP-seq peaks overlapping
1
0.8
0.6
0.4
0.2
0
Repeat class DNA LTR LINE SINE
C2H2-zf type KRAB BTB Other
TF type Dark Enhancer Promoter Other
Peak overlap
(Jaccard coefficient)
Upper triange
>=0.6
0.4
0.2
0
Motif similarity
(MoSBAT affinity correlation)
Lower triange
>=0.5
0.25
<=0
No motif

Supplementary Fig. 5. Expanded information for genomic tracks and protein expression data for 217 TF binding sites. This is a more detailed version of **Fig. 2**. Additional tracks, such as gene expression in HEK293 cells (FPKM)[1], number of total ChIP-seq peaks (at the universal threshold of MACS2 *P*-value≤10^{-10}), TF age, fraction of human protein-coding promoters (out of 20,052) covered by TF peaks, fraction of ChIP-seq peaks falling within: CpG islands, H3K4me3-positive regions, facultative heterochromatin, and constitutive heterochromatin, with the main repeat class bound by the TFs are included. The upper triangle in the bottom square is the same as **Fig. 2**, however, the lower triangle here shows the similarity between PWMs for each pair of TFs, calculated by MoSBAT[2]. Gray stripes correspond to the TFs without a selected PWM in the Codebook set. Source data are provided as a Source Data file. TF: transcription factor, FPKM: Fragments Per Kilobase of transcript per Million mapped reads, C2H2-zf: C2H2-zinc finger, MYA: Million years ago.

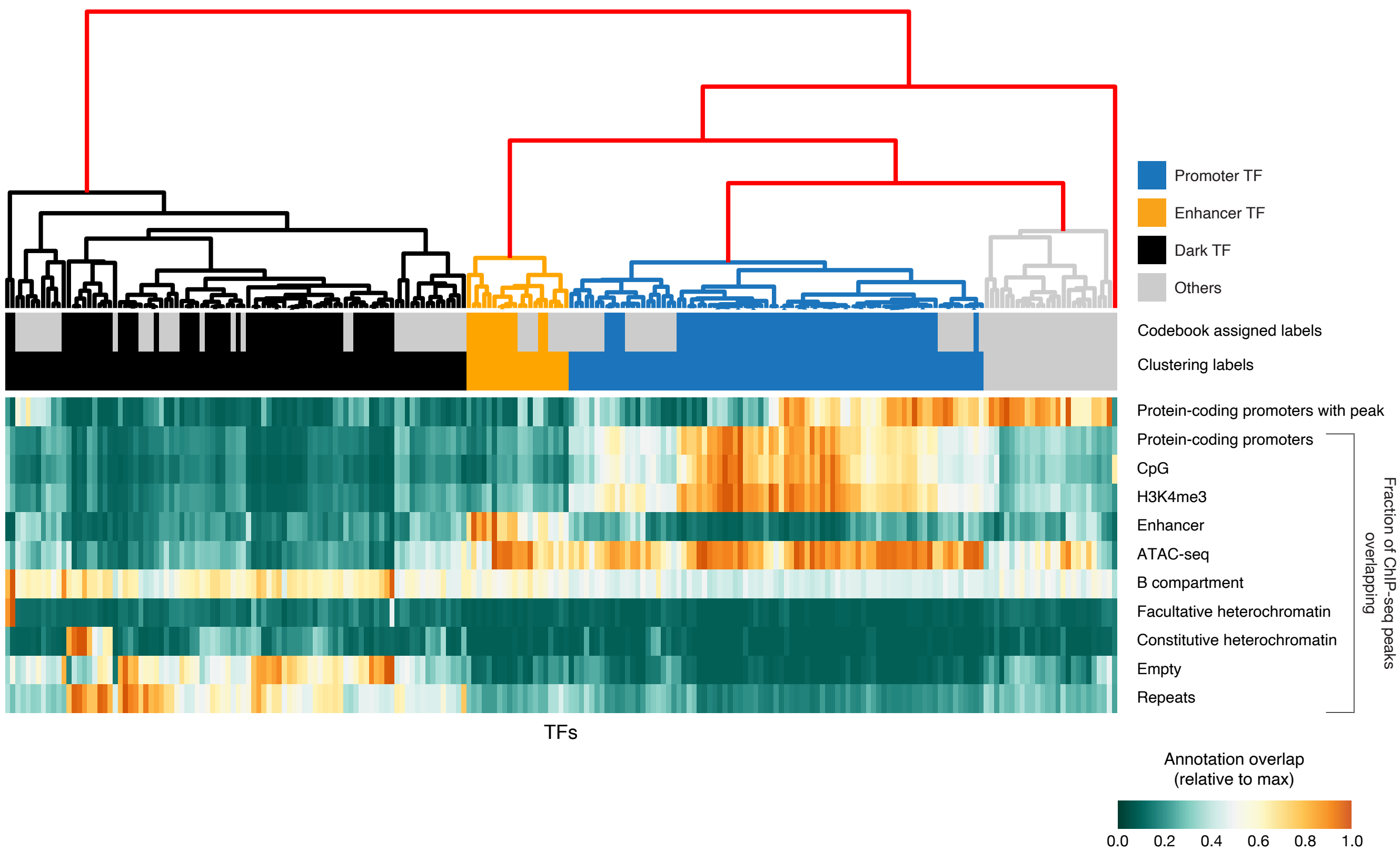

Promoter TF
Enhancer TF
Dark TF
Others
Codebook assigned labels
Clustering labels
Protein-coding promoters with peak
Protein-coding promoters
CpG
H3K4me3
Enhancer
ATAC-seq
B compartment
Facultative heterochromatin
Constitutive heterochromatin
Empty
Repeats
Fraction of ChIP-seq peaks overlapping
TFs
Annotation overlap (relative to max)
0.0
0.2
0.4
0.6
0.8
1.0

Supplementary Fig. 6. Automated clustering of the TFs and comparison with expert labeling. The 217 TFs with successful ChIP-seq data were clustered using the features presented at the top of **Fig. 2b**, applying hierarchical clustering with a correlation similarity metric and average linkage. The top five major branches (red) were then assigned to Promoter TFs, Dark TFs, and Enhancer TFs, with two branches (one containing only a single member) left as Others (see *Clustering labels*). The *Codebook assigned labels* are shown on top for comparison. TF: transcription factor.

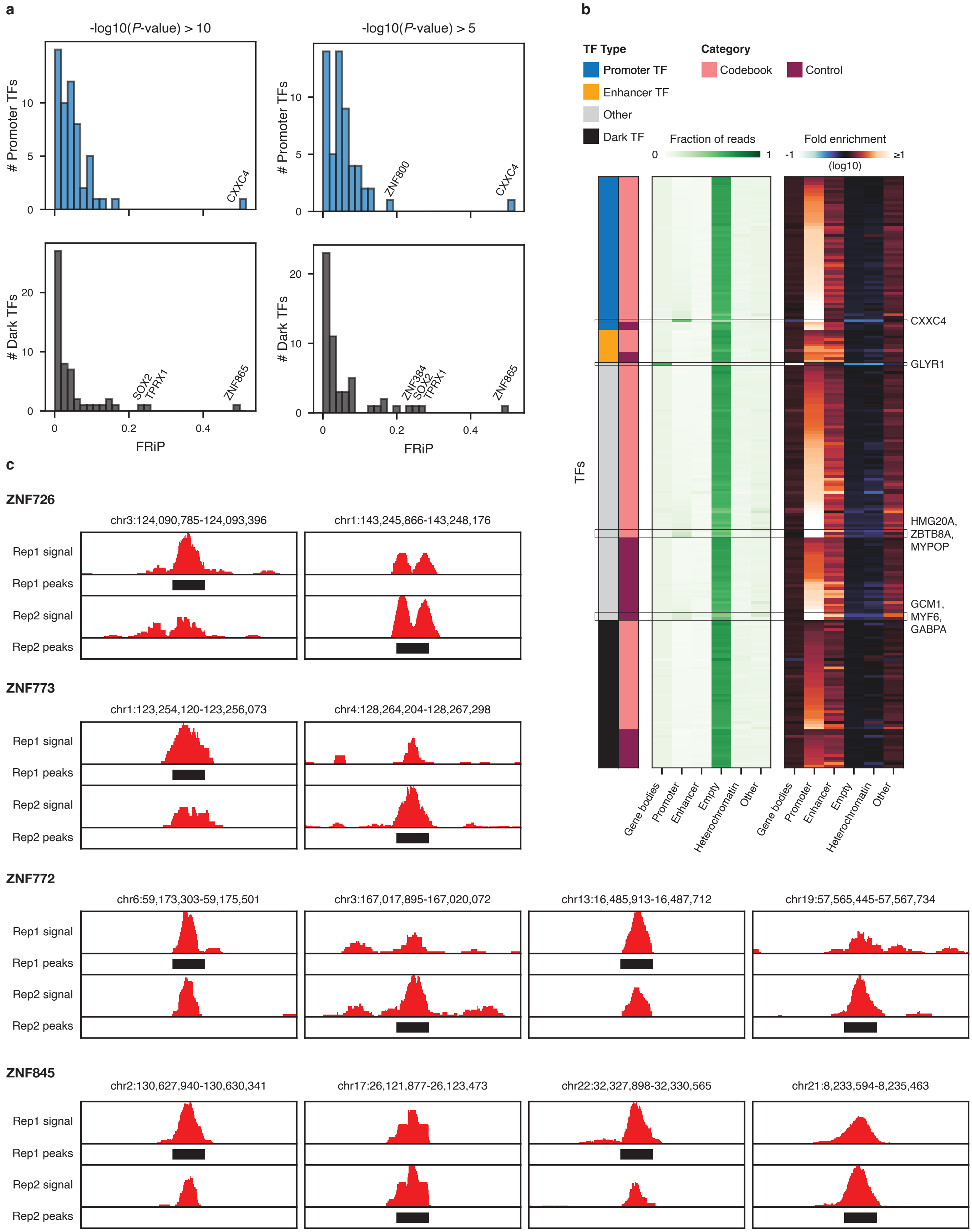
a
-log10(P-value) > 10
-log10(P-value) > 5
Promoter TFs
Dark TFs
CXXC4
ZNF800
SOX2
TPRX1
ZNF865
ZNF384
FRiP
b
TF Type
Promoter TF
Enhancer TF
Other
Dark TF
Category
Codebook
Control
Fraction of reads
Fold enrichment
(log10)
TFs
CXXC4
GLYR1
HMG20A,
ZBTB8A,
MYPOP
GCM1,
MYF6,
GABPA
Gene bodies
Promoter
Enhancer
Empty
Heterochromatin
Other
c
ZNF726
chr3:124,090,785-124,093,396
chr1:143,245,866-143,248,176
Rep1 signal
Rep1 peaks
Rep2 signal
Rep2 peaks
ZNF773
chr1:123,254,120-123,256,073
chr4:128,264,204-128,267,298
ZNF772
chr6:59,173,303-59,175,501
chr3:167,017,895-167,020,072
chr13:16,485,913-16,487,712
chr19:57,565,445-57,567,734
ZNF845
chr2:130,627,940-130,630,341
chr17:26,121,877-26,123,473
chr22:32,327,898-32,330,565
chr21:8,233,594-8,235,463

Supplementary Fig. 7. Comparative quality control for ChIP-seq peaks. (**a**) Fraction of Reads in Peaks (FRiP) for Promoter TFs and Dark TFs, at the universal threshold (MACS2 *P*-value < 1e−10) and a less stringent threshold (1e−5). (**b**) Middle heatmap: distribution of raw reads (i.e., before peak calling) in representative chromatin states (from ChromHMM) for 217 TFs with successful data. Right heatmap: $\log_{10}$ fold enrichment of the read counts (in the middle heatmap) relative to the size of each chromatin state. Labels and categories of TFs are indicated on the left. (**c**) Genome browser examples for four Dark TF, showing read pileups (red) for two replicates, at locations where both replicates show a high read coverage, but only one replicate passed the peak calling threshold (black square). Source data are provided as a Source Data file. TF: transcription factor, FRiP: Fraction of Reads in Peaks, Rep: replicate.

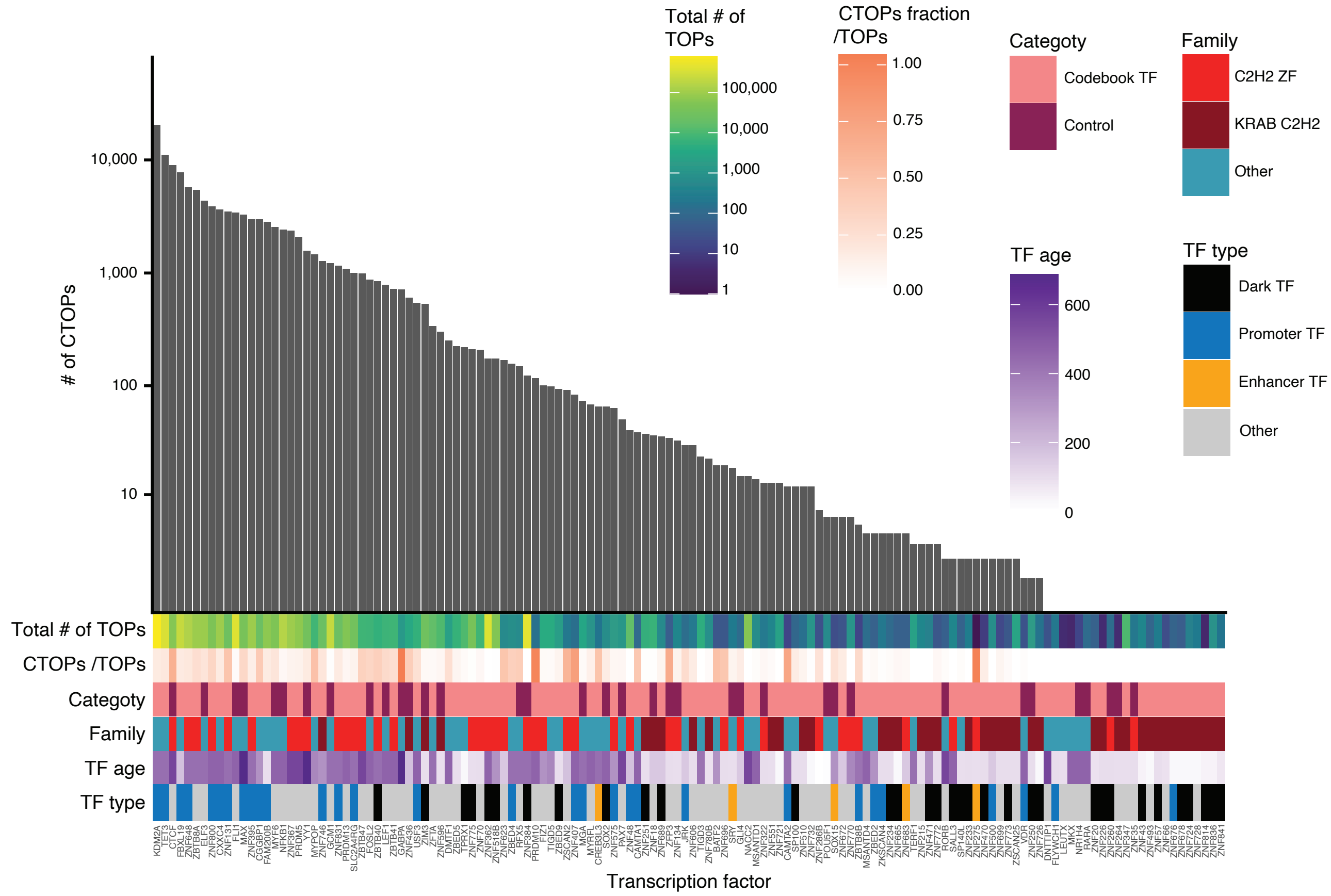

Total # of TOPs
100,000
10,000
1,000
100
10
1
CTOPs fraction /TOPs
1.00
0.75
0.50
0.25
0.00
Categoty
Codebook TF
Control
Family
C2H2 ZF
KRAB C2H2
Other
TF age
600
400
200
0
TF type
Dark TF
Promoter TF
Enhancer TF
Other
# of CTOPs
10,000
1,000
100
10
Total # of TOPs
CTOPs /TOPs
Categoty
Family
TF age
TF type
Transcription factor

Supplementary Fig. 8. Distribution of CTOP counts for 137 Codebook TFs. Bar graph displays the number of individual CTOP sites obtained for each TF. Other properties for each TF (TOP counts, CTOPs fraction/TOPs, category, DBD family, TF type, and TF age) are annotated below the bar graph. Heatmap scales are shown on the top right. **Supplementary Data 3** provides the underlying data for this figure. TF: transcription factor, TOP: triple-optimized overlap, CTOP: conserved triple-optimized overlap, C2H2-zf: C2H2-zinc finger.

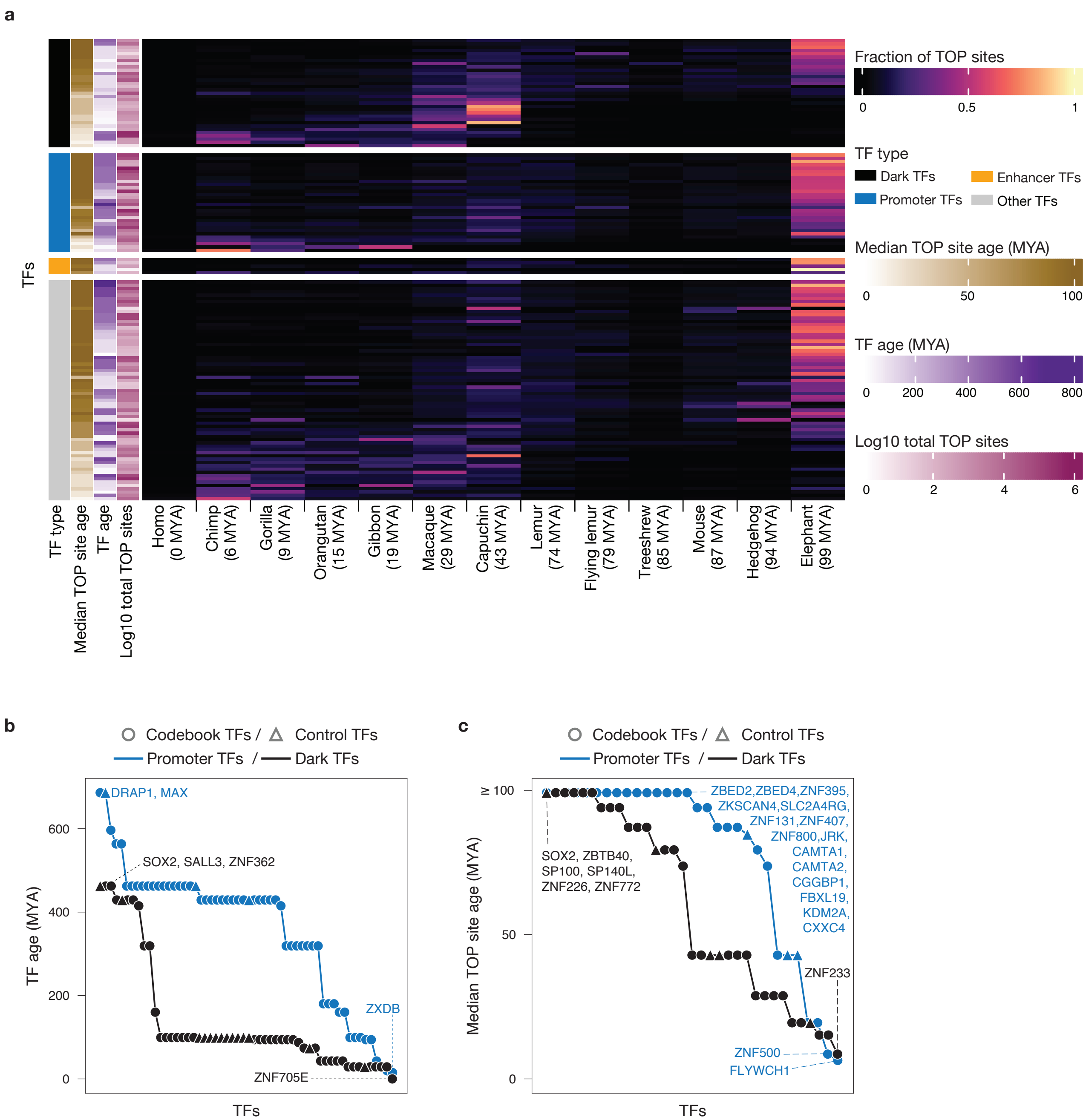

a
Fraction of TOP sites
0
0.5
1
TF type
Dark TFs
Enhancer TFs
Promoter TFs
Other TFs
Median TOP site age (MYA)
0
50
100
TF age (MYA)
0
200
400
600
800
Log10 total TOP sites
0
2
4
6
TFs
TF type
Median TOP site age
TF age
Log10 total TOP sites
Homo (0 MYA)
Chimp (6 MYA)
Gorilla (9 MYA)
Orangutan (15 MYA)
Gibbon (19 MYA)
Macaque (29 MYA)
Capuchin (43 MYA)
Lemur (74 MYA)
Flying lemur (79 MYA)
Treeshrew (85 MYA)
Mouse (87 MYA)
Hedgehog (94 MYA)
Elephant (99 MYA)
b
Codebook TFs / Control TFs
Promoter TFs / Dark TFs
DRAP1, MAX
SOX2, SALL3, ZNF362
ZXDB
ZNF705E
TF age (MYA)
600
400
200
0
TFs
c
Codebook TFs / Control TFs
Promoter TFs / Dark TFs
≥ 100
50
0
Median TOP site age (MYA)
ZBED2,ZBED4,ZNF395,
ZKSCAN4,SLC2A4RG,
ZNF131,ZNF407,
ZNF800,JRK,
CAMTA1,
CAMTA2,
CGGBP1,
FBXL19,
KDM2A,
CXXC4
SOX2, ZBTB40,
SP100, SP140L,
ZNF226, ZNF772
ZNF233
ZNF500
FLYWCH1
TFs

Supplementary Fig. 9. Age distribution relationship between TFs and their binding sites. (**a**) Heatmap showing the fraction of TOP sites for each TF, with an exact match within ancestral genomes available in Zoonomia (13 clades in human lineage up to 99 million years ago; MYA) ordered by age. In the left side for each TOP, the related TF types, median age of TOP sites, TF ages (MYA), and log 10 of total TOP sites are indicated. (**b**) The age comparison of Dark TFs and Promoter TFs. (**c**) Comparing the median age of the TOP sites between Promoter and Dark TFs in both control and Codebook categories. Source data are provided as a Source Data file. TF: transcription factor, TOP: triple-optimized overlap, MYA: Million years ago.

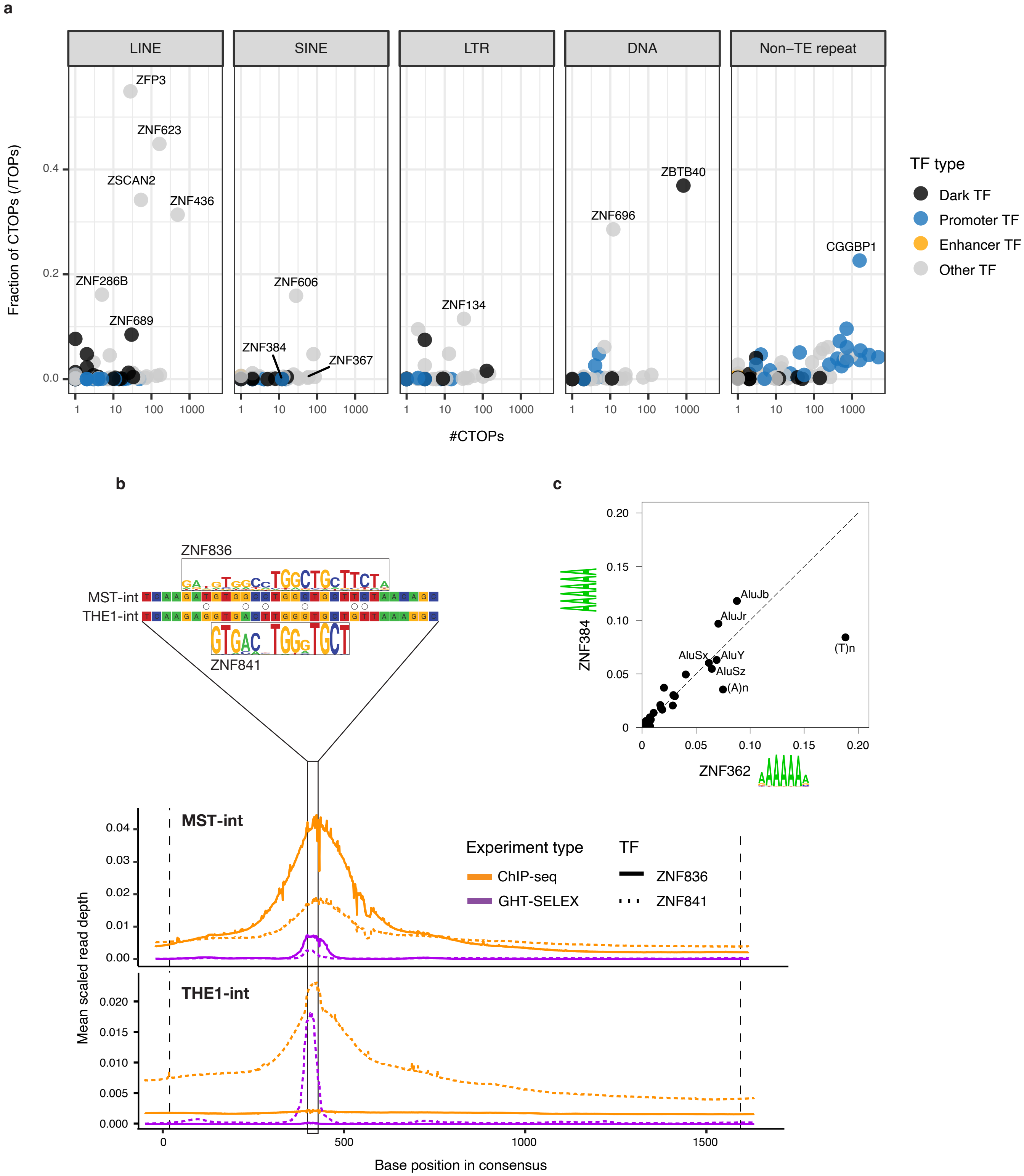

a
LINE
SINE
LTR
DNA
Non−TE repeat
Fraction of CTOPs (/TOPs)
#CTOPs
ZFP3
ZNF623
ZSCAN2
ZNF436
ZNF286B
ZNF689
ZNF606
ZNF384
ZNF367
ZNF134
ZBTB40
ZNF696
CGGBP1
TF type
Dark TF
Promoter TF
Enhancer TF
Other TF
b
ZNF836
MST-int
THE1-int
ZNF841
Mean scaled read depth
Experiment type
ChIP-seq
GHT-SELEX
TF
ZNF836
ZNF841
Base position in consensus
c
ZNF384
ZNF362
AluJb
AluJr
AluSx
AluY
AluSz
(A)n
(T)n

Supplementary Fig. 10. Codebook TFs exhibit diverse co-evolutionary interactions with repeats. (**a**) Plots showing the fraction of each TF's TOPs that are conserved (i.e. CTOPs) and overlap a major class of transposable elements or non-TE repeats. The fraction of TOPs that are conserved and overlap a repeat class is shown on the y-axis, and the log10 count of these sites is shown on the x-axis. Each TF is colored according to its classification as a Dark TF, Promoter TF, Enhancer TF, and Other TFs. Only proteins with a fraction greater than 0.1 of conserved TOPs that fall in a repeat class are labeled. TFs discussed in the main text are also labeled. (**b**) Top plot shows binding motifs of two paralogous Dark TFs, ZNF836 and ZNF841, aligned to the consensus sequences of their specific TE targets, MSTA-int and THE1-int, respectively. They bind to a homologous region in the two different but related LTR families. Circles indicate nucleotide differences between the two TEs. The bottom plot shows the average ChIP-seq and GHT-SELEX signal across all the instances of MST-int and THE1-int. (**c**) compares the fractions of TOP sites for two Dark TFs, ZNF362 and ZNF384, that overlap with various repeat elements. Both bind poly-A sequences; motifs are shown adjacent to the axis labels. Alu elements are the dominant target TEs for both TFs. Source data are provided as a Source Data file. TF: transcription factor, TOP: triple-optimized overlap, CTOP: conserved triple-optimized overlap.

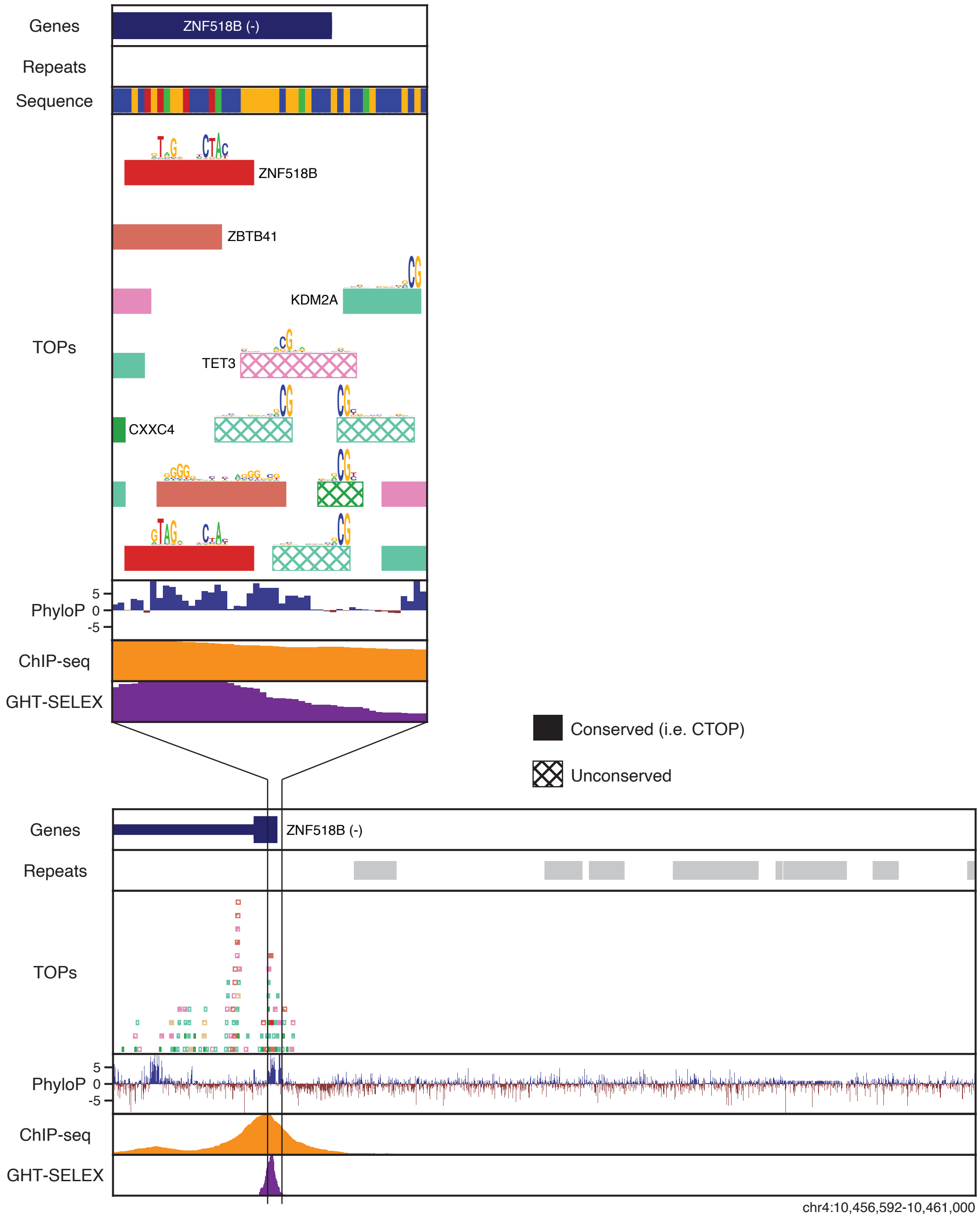
Genes
ZNF518B (-)
Repeats
Sequence
TOPs
ZNF518B
ZBTB41
KDM2A
TET3
CXXC4
PhyloP
5
0
-5
ChIP-seq
GHT-SELEX
Conserved (i.e. CTOP)
Unconserved
Genes
ZNF518B (-)
Repeats
TOPs
PhyloP
5
0
-5
ChIP-seq
GHT-SELEX
chr4:10,456,592-10,461,000

Supplementary Fig. 11. Dark TF, ZNF518B, is a potential self-regulator. Two palindromic conserved binding sites of ZNF518B (red) are in the promoter of ZNF518B itself. Binding sites of four other TFs (i.e. TOPs and CTOPs for ZBTB41, KDM2A, TET3, and CXXC4) are also present in this region. TOP: triple-optimized overlap, CTOP: conserved triple-optimized overlap.

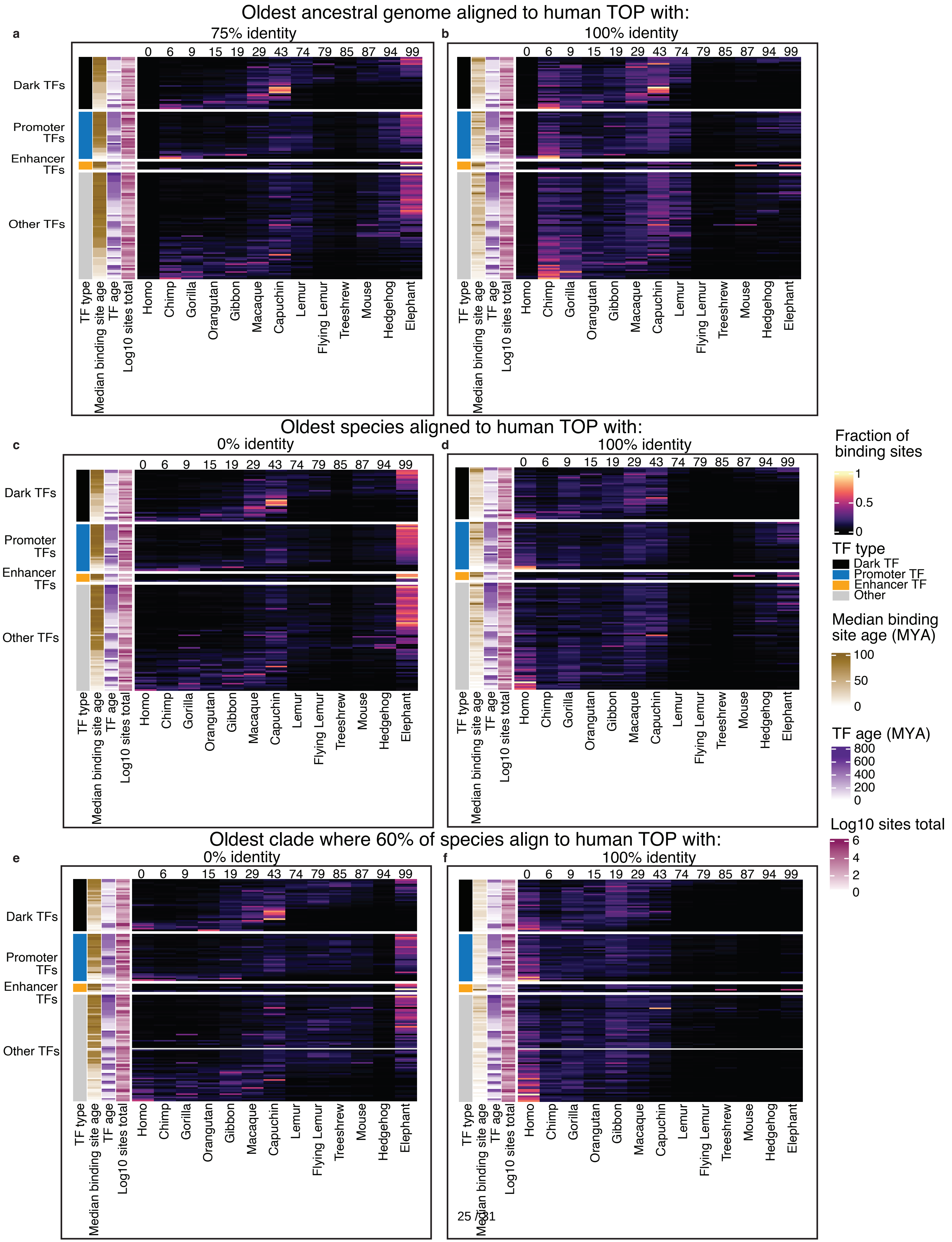
Oldest ancestral genome aligned to human TOP with:
a
75% identity
b
100% identity
Oldest species aligned to human TOP with:
c
0% identity
d
100% identity
Oldest clade where 60% of species align to human TOP with:
e
0% identity
f
100% identity
0 6 9 15 19 29 43 74 79 85 87 94 99
Dark TFs
Promoter TFs
Enhancer TFs
Other TFs
TF type
Median binding site age
TF age
Log10 sites total
Homo
Chimp
Gorilla
Orangutan
Gibbon
Macaque
Capuchin
Lemur
Flying Lemur
Treeshrew
Mouse
Hedgehog
Elephant
Fraction of binding sites
1
0.5
0
TF type
Dark TF
Promoter TF
Enhancer TF
Other
Median binding site age (MYA)
100
50
0
TF age (MYA)
800
600
400
200
0
Log10 sites total
6
4
2
0

Supplementary Fig. 12. Age estimation of TOPs. Three different tests and two different thresholds were applied. Heatmaps show the proportion of each TF's TOPs (rows) inferred to be a certain age, as in **Fig. 5**, but with each panel utilizing a different scheme. Top row: Age of each TOP site inferred as that of oldest ancestral genome with a gapless alignment to the human TOP site and minimum 75% identity (**a**) or 100% identity (**b**). (**Fig. 5** shows this same analysis with a 0% identity threshold). Middle row: Age of each TOP site inferred as that of oldest *species* with a gapless alignment to the human TOP site and minimum 0% identity (**c**) or 100% identity (**d**). Bottom row: Age of each TOP site inferred as that of the oldest clade where 60% of the species have a gapless alignment to the human TOP with a minimum 0% identity (**e**) or 100% identity (**f**). Source data are provided as a Source Data file. TF: transcription factor, TOP: triple-optimized overlap, MYA: Million years ago.

Supplementary Note 1: Overview of Dark TFs' function

We examined existing literature and databases to survey known and potential functions for the Dark TFs, and related it to the data we collected (**Fig. 7**, **Supplementary Data 6**). Most Dark TFs have apparent roles in repression of transcription. 36 out of 54 are KZNFs, and for 18 of them (and one non-KRAB TF, ZNF888), physical association with KAP1 has been verified[1,3-6]. The KZNFs may also have repressive functions beyond the recruitment of KAP1[1,3]. Five of these Dark TF KZNFs also interact with TRIM39, which itself interacts with numerous ubiquitin conjugating enzymes, H3K4 demethylase KDM1A, and dozens of other KZNFs[4,7]. Two additional Dark TF KZNFs (and two other Dark TFs) interact with TRIM33, a member of the TIF1Y complex that specifically suppresses TGFβ-responsive genes by directly interacting with the histone subunits as well as E3 ubiquitin ligase[8].

Nine of the eleven non-KRAB Dark C2H2-zf proteins also appear to contribute to the formation and maintenance of heterochromatin, by association with chromatin proteins (CBX/HP1) directly, or via recruitment of other C2H2-zf proteins. One of them, ZNF518B, was identified as a partner of both H3K27 methylase EZH2 and H3K9/H3K27 methylase G9A, and to promote H3K9me2[9]. ZNF518B and ZNF280D both associate with multiple CBX/HP1 proteins[4,7]. ZNF518B binds many primate-specific L1 elements, but its most conserved binding sites are in its own promoter, suggesting a critical negative feedback mechanism (**Supplementary Fig. 11**). In another example, ZNF516 associates with the multifunctional CTBP1/KDM1A/RCOR1 corepressor complex, and its repressive function was shown in reporter assays[10]. Intriguingly, 22 of the 47 C2H2-zf proteins, including both KRAB and non-KRAB C2H2-zf proteins (as well as transposon-derived ZBED9, SOX2, and SP100) interact with other C2H2-zf proteins, often extensively[4], suggesting a potentially widespread role in organization of chromosome topology.

Four additional Dark TFs have other potential roles in repression of transcription. Three of them are the paralogous nuclear speckle proteins SP100, SP140, and SP140L. Each contains a SAND domain, which we confirmed binds to unmethylated CG dinucleotides *in vitro*[11], and CG-containing motifs are enriched in their ChIP-seq peaks[12]. These proteins also contain PHD and BRD domains, which typically function as epigenetic readers[13]. In our ChIP-seq data, they are enriched at sites of H3K27me3 methylation (**Fig. 7**, **Supplementary Data 6**). The fourth protein is SCML4, a polycomb group protein that was included in our study because it contains an AT hook, but we did not obtain evidence for its sequence-specific DNA binding. Thus, it may be more properly described as a chromatin protein. SCML4 is reported to associate with H3K4 demethylase KDM5C[14] as well as ubiquitination factors FBXO11 and UBR1[15].

Five of the non-KZNF Dark TFs may have roles other than repression. One of them, SOX2, is a well-known pioneer factor that can bind to motif matches within unmodified closed chromatin, but is inhibited to some extent by H3K9me3[16]. Indeed, in the ChIP-seq data reported here, most (66%) of its TOP sites are in "empty" chromatin, and only 5% overlap with ATAC-seq peaks in unperturbed HEK293 cells, consistent with its pioneer function. Less than 2% of SOX2 peaks overlap with heterochromatin (defined

by ChromHMM mainly by H3K9me3 and H327me3), consistent with H3K9me3 being refractory to SOX2 binding. Two additional Dark TFs may also represent pioneers: TPRX1 has recently been described as a master regulator in zygotic genome activation[17], while SALL3 controls the differentiation of hiPSCs into cardiomyocytes vs neural cells[18]. In contrast, two other Dark TFs have been described as impacting DNA metabolism. ZNF384, which we find binds many Alu and Poly-A repeats, as described above, is also known to bind Ku and recruit NHEJ factors to double-strand breaks[19]. ZNF146 binds L1 elements, and its depletion slows the replication fork[20].

Reported physiological consequences for some the Dark TFs perturbations are available and are summarized in **Fig. 7**, right column (**Supplementary Data 6** provides the values and sources). The correspondence between recorded phenotypes and the discovered binding sites is discussed in the main text. Expanding on one Dark TF, ZBTB40, its most conserved TOP resides within the 3'UTR of PRKACA (**Fig. 5g**). PRKACA encodes the catalytic subunit α of protein kinase A, whose deficiency is associated with fertility defects in male mice and humans[21]. This site is also less than 1 kb from the TSS of the chromatin regulator SAMD1, which impacts sperm cells[22].

Supplementary Note 2: Analysis of binding site ages for TFs

In addition to insertions such as TEs, new TF binding sites can emerge from random mutations in pre-existing sequences. This mechanism is thought to be dominant for traditional enhancer-binding TFs[23]. To determine whether binding sites for Codebook TFs evolved from ancestral DNA, we estimated the age of each TOP site for each TF.

We gauged binding site age as that of the oldest ancestral genome that contains the entire site, using halLiftover[24] to map TOP PWM hits to syntenic loci in all other genomes in the Zoonomia 240-mammal alignment[25], including 9 reconstructed genomes ancestral to human, and calculated each sequence's % identity to the aligned human TOP.

We then assigned an age using multiple criteria, identifying the oldest ancestral genome with a gapless alignment of any % identity to the human PWM hit (**Supplementary Fig. 9a).** This is a simple heuristic, but we obtained a qualitatively similar conclusions using other approaches to estimate binding site age (**Supplementary Fig. 12**), including the oldest extant *species* with a gapless alignment to a human TOP (at various threshold % identities), or the oldest *clade* wherein 60% of species have a gapless alignment to the human TOP (at various threshold % identities). We acquired the age of each clade from TimeTree[26].

As expected, TOP sites for the Dark TFs (most of which correspond to TEs) are estimated to be considerably younger on average than those of Promoter TFs (median ages of 19.5 and >=99.2 MYA, respectively, **Supplementary Fig. 9a**)**,** but there is a large overlap of age distributions between the two TF groups. Both groups also contain TFs with binding sites at both extremes (i.e. very old or very young binding sites). Thus, average age of the binding site is not a discriminating characteristic of Promoter vs. Dark TFs.

We also estimated the ages of the TFs, by first acquiring all vertebrate ortholog annotations and ortholog quality statistics for each TF from Ensembl[27], and ages of each pair of species from TimeTree[26] (**Supplementary Fig. 9c**, **Supplementary Data 3**). The age of a TF was taken as the oldest ortholog annotated as having a 1-1 relationship with human, or having a gene order conservation (GOC, a metric of synteny) score >= 50 and classification as a high confidence ortholog by Ensembl.

Overall, TFs in both classes tend to be older than the sites they bind: Promoter TFs have a median age of 429 MYA, while Dark TFs have a median age of 99.19 MYA, which is approximately as old as a typical binding site even for Promoter TFs. These results are consistent with the established phenomenon of TF binding site turnover[23]. They are also reminiscent of previous observations with KZNFs showing weak correlation between the age of the KZNFs and the age of their binding sites, and inconsistent with a model in which the KZNFs evolve only to silence TEs[28]. Together with the retention of many KZNFs that bind extinct TEs, this finding supports the notion that KZNFs must frequently take on additional roles, e.g., in regulation of host genes.

SUPPLEMENTARY REFERENCES

1. Schmitges, F.W. *et al.* Multiparameter functional diversity of human C2H2 zinc finger proteins. *Genome Res* **26**, 1742–1752 (2016).
2. Lambert, S.A., Albu, M., Hughes, T.R. & Najafabadi, H.S. Motif comparison based on similarity of binding affinity profiles. *Bioinformatics* **32**, 3504–3506 (2016).
3. Helleboid, P.Y. *et al.* The interactome of KRAB zinc finger proteins reveals the evolutionary history of their functional diversification. *EMBO J* **38**, e101220 (2019).
4. Huttlin, E.L. *et al.* Dual proteome-scale networks reveal cell-specific remodeling of the human interactome. *Cell* **184**, 3022–3040 e28 (2021).
5. Silva, F.P., Hamamoto, R., Furukawa, Y. & Nakamura, Y. TIPUH1 encodes a novel KRAB zinc-finger protein highly expressed in human hepatocellular carcinomas. *Oncogene* **25**, 5063–70 (2006).
6. Kim, J.J. *et al.* Systematic bromodomain protein screens identify homologous recombination and R-loop suppression pathways involved in genome integrity. *Genes Dev* **33**, 1751–1774 (2019).
7. Oughtred, R. *et al.* The BioGRID database: A comprehensive biomedical resource of curated protein, genetic, and chemical interactions. *Protein Sci* **30**, 187–200 (2021).
8. Agricola, E., Randall, R.A., Gaarenstroom, T., Dupont, S. & Hill, C.S. Recruitment of TIF1gamma to chromatin via its PHD finger-bromodomain activates its ubiquitin ligase and transcriptional repressor activities. *Mol Cell* **43**, 85–96 (2011).
9. Maier, V.K. *et al.* Functional Proteomic Analysis of Repressive Histone Methyltransferase Complexes Reveals ZNF518B as a G9A Regulator. *Mol Cell Proteomics* **14**, 1435–46 (2015).
10. Li, L. *et al.* ZNF516 suppresses EGFR by targeting the CtBP/LSD1/CoREST complex to chromatin. *Nat Commun* **8**, 691 (2017).
11. Huggenvik, J.I. *et al.* Characterization of a nuclear deformed epidermal autoregulatory factor-1 (DEAF-1)-related (NUDR) transcriptional regulator protein. *Mol Endocrinol* **12**, 1619–39 (1998).
12. Vorontsov, I.E. *et al.* Cross-platform DNA motif discovery and benchmarking to explore binding specificities of poorly studied human transcription factors. *bioRxiv*, 2024.11.11.619379 (2024).
13. Fraschilla, I. & Jeffrey, K.L. The Speckled Protein (SP) Family: Immunity's Chromatin Readers. *Trends Immunol* **41**, 572–585 (2020).
14. Tumber, A. *et al.* Potent and Selective KDM5 Inhibitor Stops Cellular Demethylation of H3K4me3 at Transcription Start Sites and Proliferation of MM1S Myeloma Cells. *Cell Chem Biol* **24**, 371–380 (2017).
15. Huttlin, E.L. *et al.* The BioPlex Network: A Systematic Exploration of the Human Interactome. *Cell* **162**, 425–440 (2015).
16. Soufi, A., Donahue, G. & Zaret, K.S. Facilitators and impediments of the pluripotency reprogramming factors' initial engagement with the genome. *Cell* **151**, 994–1004 (2012).
17. Zou, Z. *et al.* Translatome and transcriptome co-profiling reveals a role of TPRXs in human zygotic genome activation. *Science* **378**, abo7923 (2022).

18. Kuroda, T. *et al.* SALL3 expression balance underlies lineage biases in human induced pluripotent stem cell differentiation. *Nat Commun* **10**, 2175 (2019).
19. Singh, J.K. *et al.* Zinc finger protein ZNF384 is an adaptor of Ku to DNA during classical non-homologous end-joining. *Nat Commun* **12**, 6560 (2021).
20. Feu, S. *et al.* OZF is a Claspin-interacting protein essential to maintain the replication fork progression rate under replication stress. *FASEB J* **34**, 6907–6919 (2020).
21. Burton, K.A. *et al.* Haploinsufficiency at the protein kinase A RI alpha gene locus leads to fertility defects in male mice and men. *Mol Endocrinol* **20**, 2504–13 (2006).
22. Stielow, B. *et al.* The SAM domain-containing protein 1 (SAMD1) acts as a repressive chromatin regulator at unmethylated CpG islands. *Sci Adv* **7**(2021).
23. Villar, D. *et al.* Enhancer evolution across 20 mammalian species. *Cell* **160**, 554–66 (2015).
24. Hickey, G., Paten, B., Earl, D., Zerbino, D. & Haussler, D. HAL: a hierarchical format for storing and analyzing multiple genome alignments. *Bioinformatics* **29**, 1341–2 (2013).
25. Armstrong, J. *et al.* Progressive Cactus is a multiple-genome aligner for the thousand-genome era. *Nature* **587**, 246–251 (2020).
26. Kumar, S. *et al.* TimeTree 5: An Expanded Resource for Species Divergence Times. *Mol Biol Evol* **39**(2022).
27. Dyer, S.C. *et al.* Ensembl 2025. *Nucleic Acids Res* **53**, D948–D957 (2025).
28. Imbeault, M., Helleboid, P.Y. & Trono, D. KRAB zinc-finger proteins contribute to the evolution of gene regulatory networks. *Nature* **543**, 550–554 (2017).
