# Supplementary material for: Extensive binding of poorly characterized human transcription factors to genomic dark matter
Source: Nat Commun. 2026 Aug 5;17:7770. doi: 10.1038/s41467-026-75376-z (PMC13444102; doi:10.1038/s41467-026-75376-z)
Supplement: Supplementary file 2 [file 41467_2026_75376_MOESM5_ESM.pdf]

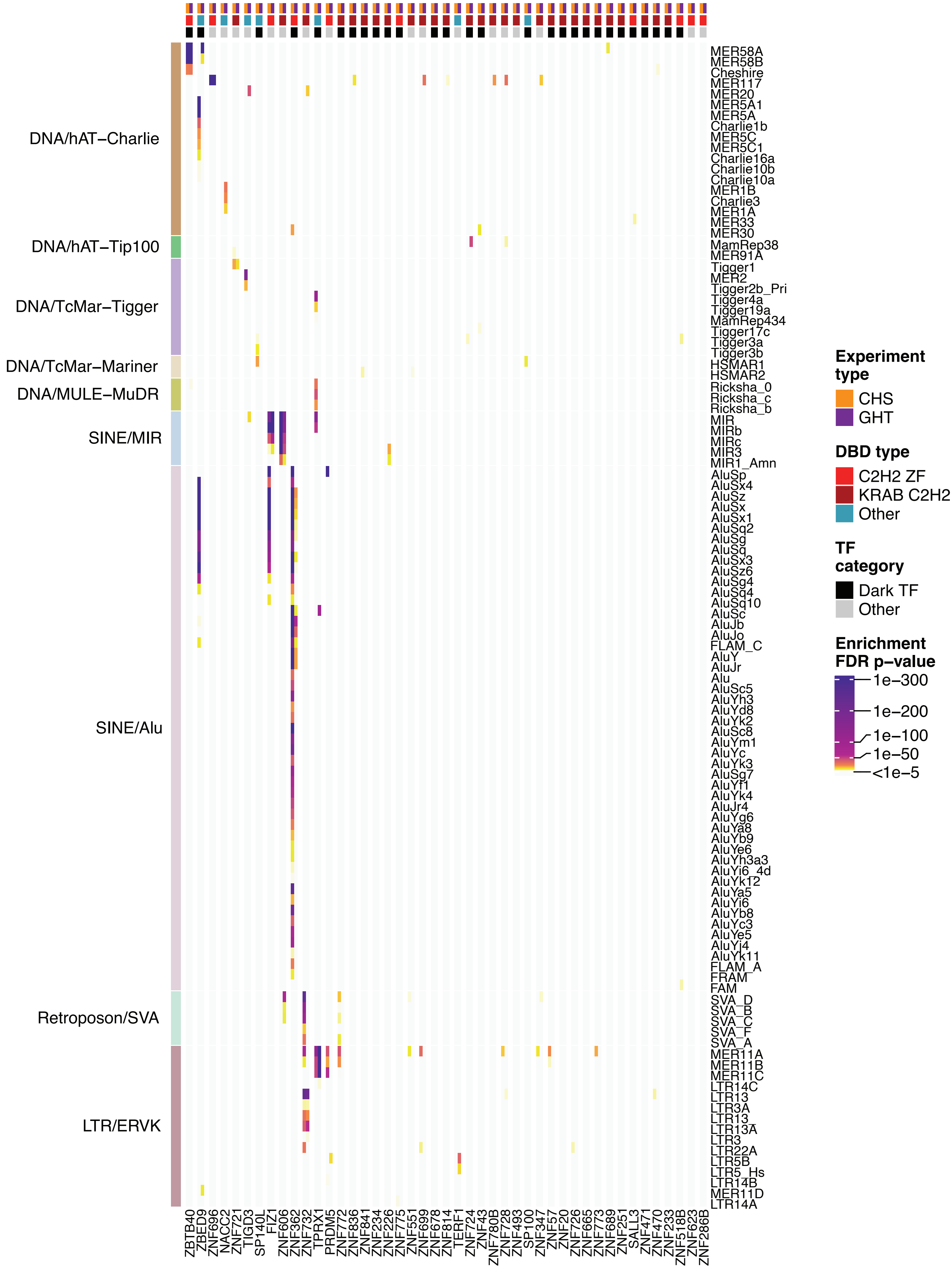
DNA/hAT−Charlie
DNA/hAT−Tip100
DNA/TcMar−Tigger
DNA/TcMar−Mariner
DNA/MULE−MuDR
SINE/MIR
SINE/Alu
Retroposon/SVA
LTR/ERVK
MER58A
MER58B
Cheshire
MER117
MER20
MER5A1
MER5A
Charlie1b
MER5C
MER5C1
Charlie16a
Charlie10b
Charlie10a
MER1B
Charlie3
MER1A
MER33
MER30
MamRep38
MER91A
Tigger1
MER2
Tigger2b_Pri
Tigger4a
Tigger19a
MamRep434
Tigger17c
Tigger3a
Tigger3b
HSMAR1
HSMAR2
Ricksha_0
Ricksha_c
Ricksha_b
MIR
MIRb
MIRc
MIR3
MIR1_Amn
AluSp
AluSx4
AluSz
AluSx
AluSx1
AluSq2
AluSg
AluSq
AluSx3
AluSz6
AluSg4
AluSq4
AluSq10
AluSc
AluJb
AluJo
FLAM_C
AluY
AluJr
Alu
AluSc5
AluYh3
AluYd8
AluYk2
AluSc8
AluYm1
AluYc
AluYk3
AluSg7
AluYf1
AluYk4
AluJr4
AluYg6
AluYa8
AluYb9
AluYe6
AluYh3a3
AluYi6_4d
AluYk12
AluYa5
AluYi6
AluYb8
AluYc3
AluYe5
AluYj4
AluYk11
FLAM_A
FRAM
FAM
SVA_D
SVA_B
SVA_C
SVA_F
SVA_A
MER11A
MER11B
MER11C
LTR14C
LTR13
LTR3A
LTR13
LTR13A
LTR3
LTR22A
LTR5B
LTR5_Hs
LTR14B
MER11D
LTR14A
ZBTB40
ZBED9
ZNF696
NACC2
ZNF721
TIGD3
SP140L
FIZ1
ZNF606
ZNF362
ZNF732
TPRX1
PRDM5
ZNF772
ZNF836
ZNF841
ZNF234
ZNF226
ZNF775
ZNF551
ZNF699
ZNF678
ZNF814
TERF1
ZNF724
ZNF43
ZNF780B
ZNF728
ZNF493
SP100
ZNF347
ZNF57
ZNF20
ZNF726
ZNF665
ZNF773
ZNF689
ZNF251
SALL3
ZNF471
ZNF470
ZNF233
ZNF518B
ZNF623
ZNF286B
Experiment type
CHS
GHT
DBD type
C2H2 ZF
KRAB C2H2
Other
TF category
Dark TF
Other
Enrichment FDR p−value
1e−300
1e−200
1e−100
1e−50
<1e−5

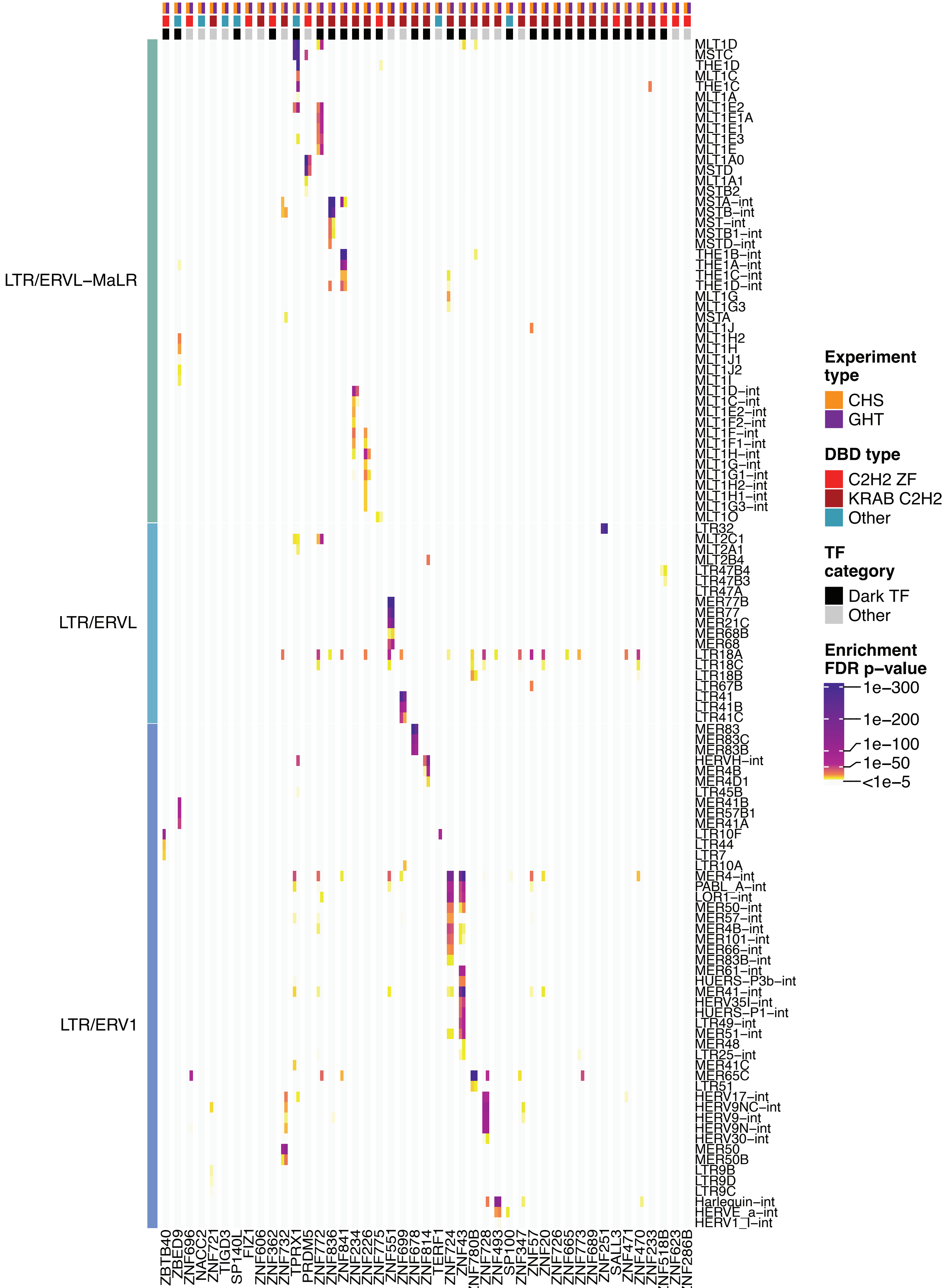

LTR/ERVL−MaLR
LTR/ERVL
LTR/ERV1
MLT1D
MSTC
THE1D
MLT1C
THE1C
MLT1A
MLT1E2
MLT1E1A
MLT1E1
MLT1E3
MLT1E
MLT1A0
MSTD
MLT1A1
MSTB2
MSTA−int
MSTB−int
MST−int
MSTB1−int
MSTD−int
THE1B−int
THE1A−int
THE1C−int
THE1D−int
MLT1G
MLT1G3
MSTA
MLT1J
MLT1H2
MLT1H
MLT1J1
MLT1J2
MLT1I
MLT1D−int
MLT1C−int
MLT1E2−int
MLT1F2−int
MLT1F−int
MLT1F1−int
MLT1H−int
MLT1G−int
MLT1G1−int
MLT1H2−int
MLT1H1−int
MLT1G3−int
MLT1O
LTR32
MLT2C1
MLT2A1
MLT2B4
LTR47B4
LTR47B3
LTR47A
MER77B
MER77
MER21C
MER68B
MER68
LTR18A
LTR18C
LTR18B
LTR67B
LTR41
LTR41B
LTR41C
MER83
MER83C
MER83B
HERVH−int
MER4B
MER4D1
LTR45B
MER41B
MER57B1
MER41A
LTR10F
LTR44
LTR7
LTR10A
MER4−int
PABL_A−int
LOR1−int
MER50−int
MER57−int
MER4B−int
MER101−int
MER66−int
MER83B−int
MER61−int
HUERS−P3b−int
MER41−int
HERV35I−int
HUERS−P1−int
LTR49−int
MER51−int
MER48
LTR25−int
MER41C
MER65C
LTR51
HERV17−int
HERV9NC−int
HERV9−int
HERV9N−int
HERV30−int
MER50
MER50B
LTR9B
LTR9D
LTR9C
Harlequin−int
HERVE_a−int
HERV1_I−int
ZBTB40
ZBED9
ZNF696
NACC2
ZNF721
TIGD3
SP140L
FIZ1
ZNF606
ZNF362
ZNF732
TPRX1
PRDM5
ZNF772
ZNF836
ZNF841
ZNF234
ZNF226
ZNF775
ZNF551
ZNF699
ZNF678
ZNF814
TERF1
ZNF724
ZNF43
ZNF780B
ZNF728
ZNF493
SP100
ZNF347
ZNF57
ZNF20
ZNF726
ZNF665
ZNF773
ZNF689
ZNF251
SALL3
ZNF471
ZNF470
ZNF233
ZNF518B
ZNF623
ZNF286B
Experiment type
CHS
GHT
DBD type
C2H2 ZF
KRAB C2H2
Other
TF category
Dark TF
Other
Enrichment FDR p−value
1e−300
1e−200
1e−100
1e−50
<1e−5

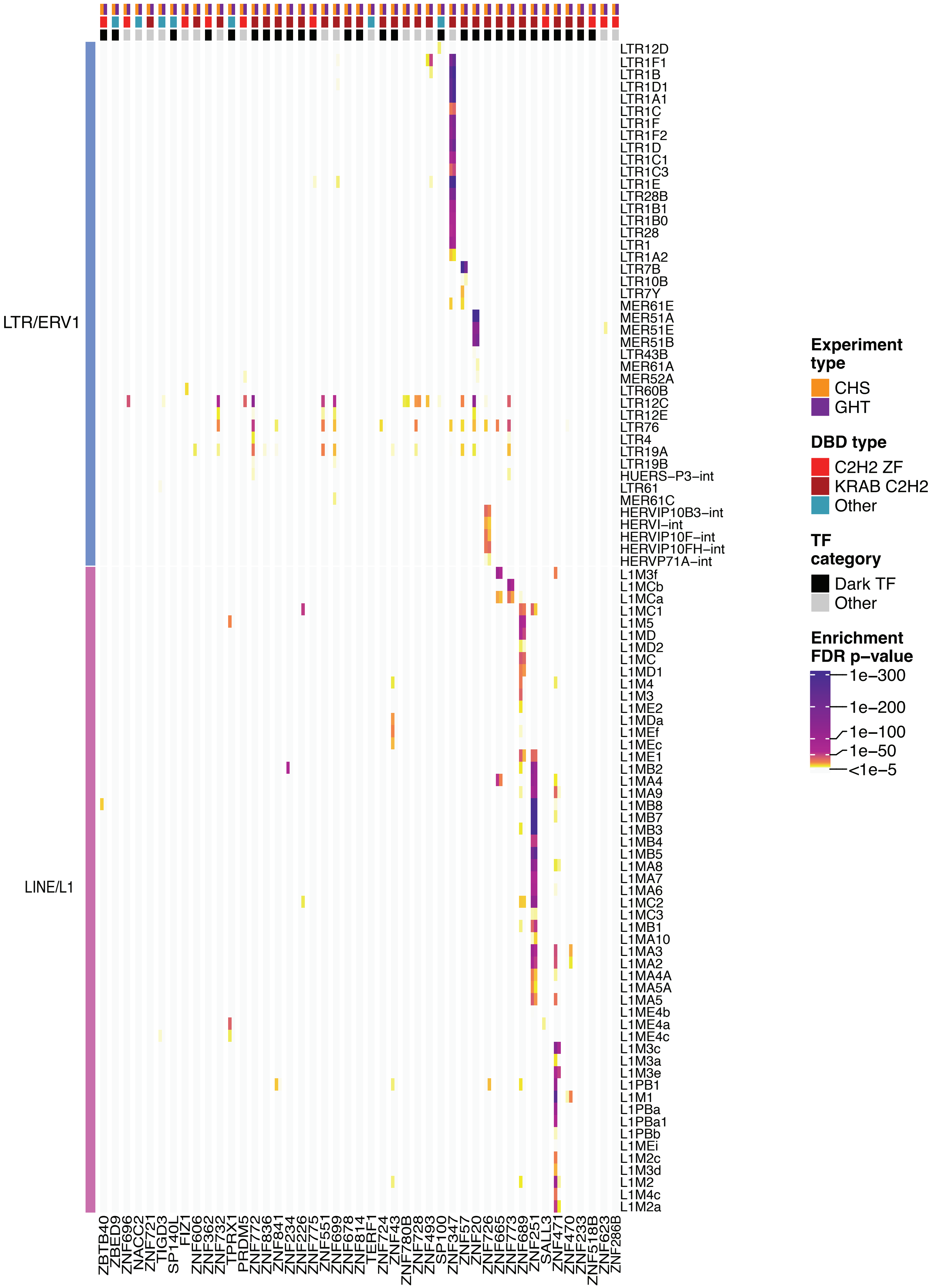

LTR/ERV1
LINE/L1
LTR12D
LTR1F1
LTR1B
LTR1D1
LTR1A1
LTR1C
LTR1F
LTR1F2
LTR1D
LTR1C1
LTR1C3
LTR1E
LTR28B
LTR1B1
LTR1B0
LTR28
LTR1
LTR1A2
LTR7B
LTR10B
LTR7Y
MER61E
MER51A
MER51E
MER51B
LTR43B
MER61A
MER52A
LTR60B
LTR12C
LTR12E
LTR76
LTR4
LTR19A
LTR19B
HUERS−P3−int
LTR61
MER61C
HERVIP10B3−int
HERVI−int
HERVIP10F−int
HERVIP10FH−int
HERVP71A−int
L1M3f
L1MCb
L1MCa
L1MC1
L1M5
L1MD
L1MD2
L1MC
L1MD1
L1M4
L1M3
L1ME2
L1MDa
L1MEf
L1MEc
L1ME1
L1MB2
L1MA4
L1MA9
L1MB8
L1MB7
L1MB3
L1MB4
L1MB5
L1MA8
L1MA7
L1MA6
L1MC2
L1MC3
L1MB1
L1MA10
L1MA3
L1MA2
L1MA4A
L1MA5A
L1MA5
L1ME4b
L1ME4a
L1ME4c
L1M3c
L1M3a
L1M3e
L1PB1
L1M1
L1PBa
L1PBa1
L1PBb
L1MEi
L1M2c
L1M3d
L1M2
L1M4c
L1M2a
ZBTB40
ZBED9
ZNF696
NACC2
ZNF721
TIGD3
SP140L
FIZ1
ZNF606
ZNF362
ZNF732
TPRX1
PRDM5
ZNF772
ZNF836
ZNF841
ZNF234
ZNF226
ZNF775
ZNF551
ZNF699
ZNF678
ZNF814
TERF1
ZNF724
ZNF43
ZNF780B
ZNF728
ZNF493
SP100
ZNF347
ZNF57
ZNF20
ZNF726
ZNF665
ZNF773
ZNF689
ZNF251
SALL3
ZNF471
ZNF470
ZNF233
ZNF518B
ZNF623
ZNF286B
Experiment type
CHS
GHT
DBD type
C2H2 ZF
KRAB C2H2
Other
TF category
Dark TF
Other
Enrichment FDR p−value
1e−300
1e−200
1e−100
1e−50
<1e−5

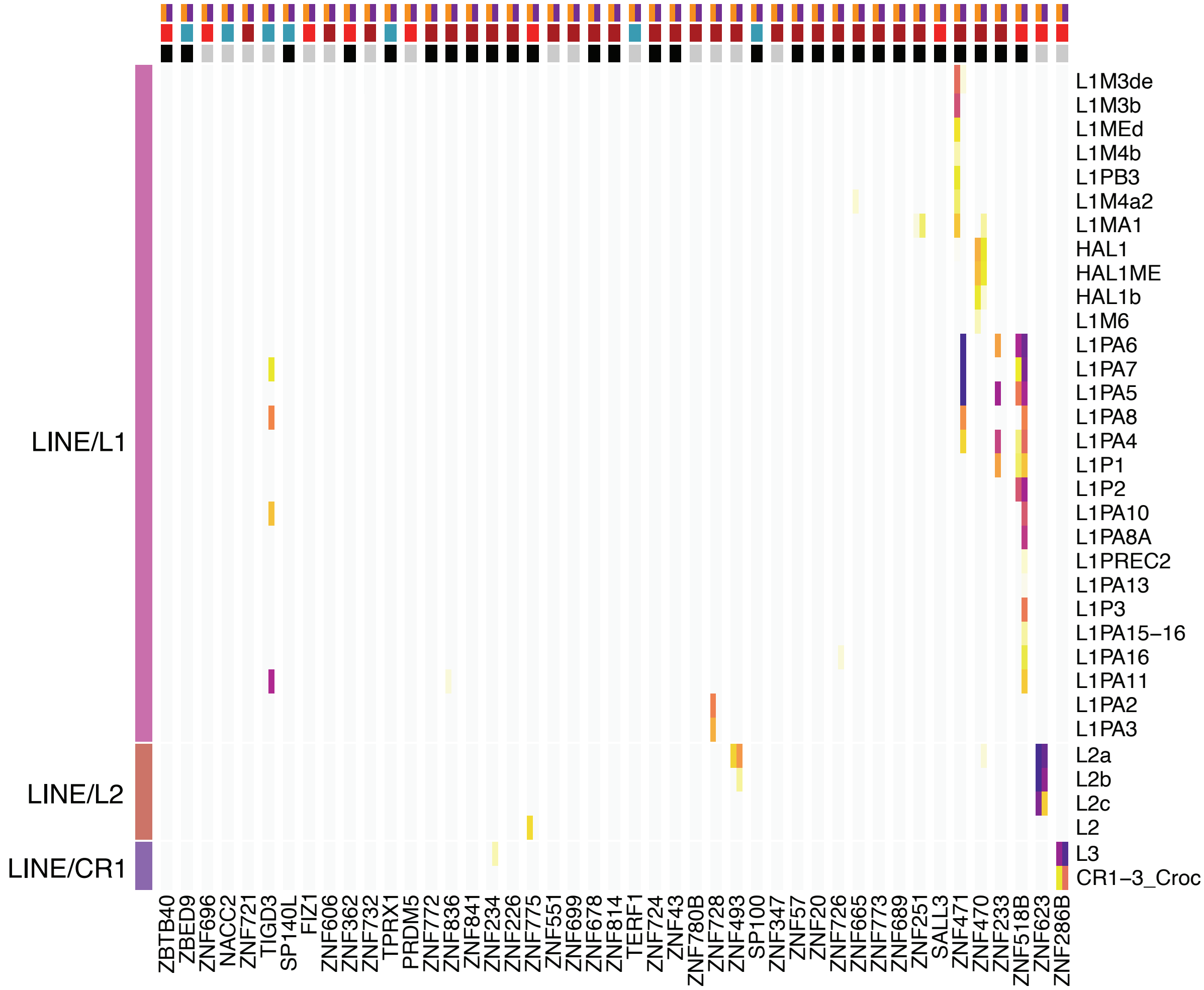
LINE/L1
LINE/L2
LINE/CR1
L1M3de
L1M3b
L1MEd
L1M4b
L1PB3
L1M4a2
L1MA1
HAL1
HAL1ME
HAL1b
L1M6
L1PA6
L1PA7
L1PA5
L1PA8
L1PA4
L1P1
L1P2
L1PA10
L1PA8A
L1PREC2
L1PA13
L1P3
L1PA15−16
L1PA16
L1PA11
L1PA2
L1PA3
L2a
L2b
L2c
L2
L3
CR1−3_Croc
ZBTB40
ZBED9
ZNF696
NACC2
ZNF721
TIGD3
SP140L
FIZ1
ZNF606
ZNF362
ZNF732
TPRX1
PRDM5
ZNF772
ZNF836
ZNF841
ZNF234
ZNF226
ZNF775
ZNF551
ZNF699
ZNF678
ZNF814
TERF1
ZNF724
ZNF43
ZNF780B
ZNF728
ZNF493
SP100
ZNF347
ZNF57
ZNF20
ZNF726
ZNF665
ZNF773
ZNF689
ZNF251
SALL3
ZNF471
ZNF470
ZNF233
ZNF518B
ZNF623
ZNF286B
