# Supplementary material for: Extensive binding of poorly characterized human transcription factors to genomic dark matter
Source: Nat Commun. 2026 Aug 5;17:7770. doi: 10.1038/s41467-026-75376-z (PMC13444102; doi:10.1038/s41467-026-75376-z)
Supplement: Supplementary file 4 [file 41467_2026_75376_MOESM8_ESM.pdf]

nature portfolio

Corresponding author(s): Timothy R. Hughes

Last updated by author(s): May 18, 2026

Reporting Summary

Nature Portfolio wishes to improve the reproducibility of the work that we publish. This form provides structure for consistency and transparency in reporting. For further information on Nature Portfolio policies, see our Editorial Policies and the Editorial Policy Checklist.

Statistics

For all statistical analyses, confirm that the following items are present in the figure legend, table legend, main text, or Methods section.

n/a	Confirmed	
☐	☒	The exact sample size (*n*) for each experimental group/condition, given as a discrete number and unit of measurement
☐	☒	A statement on whether measurements were taken from distinct samples or whether the same sample was measured repeatedly
☐	☒	The statistical test(s) used AND whether they are one- or two-sided<br>*Only common tests should be described solely by name; describe more complex techniques in the Methods section.*
☒	☐	A description of all covariates tested
☐	☒	A description of any assumptions or corrections, such as tests of normality and adjustment for multiple comparisons
☐	☒	A full description of the statistical parameters including central tendency (e.g. means) or other basic estimates (e.g. regression coefficient) AND variation (e.g. standard deviation) or associated estimates of uncertainty (e.g. confidence intervals)
☐	☒	For null hypothesis testing, the test statistic (e.g. *F*, *t*, *r*) with confidence intervals, effect sizes, degrees of freedom and *P* value noted<br>*Give P values as exact values whenever suitable.*
☒	☐	For Bayesian analysis, information on the choice of priors and Markov chain Monte Carlo settings
☒	☐	For hierarchical and complex designs, identification of the appropriate level for tests and full reporting of outcomes
☐	☒	Estimates of effect sizes (e.g. Cohen's *d*, Pearson's *r*), indicating how they were calculated

Our web collection on statistics for biologists contains articles on many of the points above.

Software and code

Policy information about availability of computer code

Data collection: No software was used in data collection.

Data analysis: Python 3, R (v4.3.2), and BEDTools (v2.30.0) were used for various analyses and calculations.
Position weight matrices (PWMs) were identified using numerous tools, described in an accompanying manuscript (Vorontsov et al., 2025).
For read processing and mapping, cutadapt (v4.9), bowtie2 (v2.4.1), and samtools (v1.16.1), sambamba (v1.0.1), Picard (v3.3.0) were used.
PWM scanning was performed using MOODS (v1.9.4).
ChIP-seq peak calling was performed with MACS2 (v2.2.9.1).
MACS3 (v3.0.2) was used for ATAC-seq and H3K9me3 ChIP-seq peak calling.
GHT-SELEX peaks were called using MAGIX: https://github.com/csglab/MAGIX.
To make some of the bigwig files, UCSC tool bedGraphToBigWig (v2.9) was used.
DeepTools (v3.5.3) was used to prepare the matrix of phyloP values around TOP sites (computeMatrix) and to shift ATAC-seq ends (alignmentSieve).
Code for triple overlap site identification and conservation analysis is available at https://github.com/imyellan/Codebook_CTOP_scripts.
SciPy (v1.14.0) was used to calculate Fisher's exact test scores.
MoSBAT (v1) was used to calculate motif similarities in Supplementary Fig. 5.

For manuscripts utilizing custom algorithms or software that are central to the research but not yet described in published literature, software must be made available to editors and reviewers. We strongly encourage code deposition in a community repository (e.g. GitHub). See the Nature Portfolio guidelines for submitting code & software for further information.

Data

Policy information about availability of data

All manuscripts must include a data availability statement. This statement should provide the following information, where applicable:

- Accession codes, unique identifiers, or web links for publicly available datasets
- A description of any restrictions on data availability
- For clinical datasets or third party data, please ensure that the statement adheres to our policy

The sequencing raw data for the experiments have been deposited in the SRA database under identifiers PRJEB78913 [https://www.ebi.ac.uk/ena/browser/view/PRJEB78913] (ChIP-seq), PRJEB76622 [https://www.ebi.ac.uk/ena/browser/view/PRJEB76622] (GHT-SELEX), and PRJEB61115 [https://www.ebi.ac.uk/ena/browser/view/PRJEB61115] (HT-SELEX). Genomic interval information generated for the ChIP-seq and GHT-SELEX has been deposited in GEO under accessions GSE280248 [https://www.ncbi.nlm.nih.gov/geo/query/acc.cgi?acc=GSE280248] (ChIP-seq genomic intervals) and GSE278858 [https://www.ncbi.nlm.nih.gov/geo/query/acc.cgi?acc=GSE278858] (GHT-SELEX genomic intervals), respectively. Information on constructs, experiments, analyses, processed data, comparison tracks, including many accessory files and browsable results, is available at https://codebook.ccbr.utoronto.ca. A larger motif collection from an accompanying study can be browsed at https://mex.autosome.org and downloaded at https://doi.org/10.5281/ZENODO.8327372. Genomic tracks for histone marks were downloaded from ENCODE, with the accession codes ENCFF351MDO [https://www.encodeproject.org/files/ENCFF351MDO] (H3K4me1), ENCFF617NUV [https://www.encodeproject.org/files/ENCFF617NUV] (H3K4me3), ENCFF655QDU [https://www.encodeproject.org/files/ENCFF655QDU] (H3K36me3), and ENCFF225FPN [https://www.encodeproject.org/files/ENCFF225FPN] (H3K27ac), or from public data on GEO, under the accession codes GSM4859393 [https://www.ncbi.nlm.nih.gov/geo/query/acc.cgi?acc=GSM4859393] (H3K9me3), and GSM4859391 [https://www.ncbi.nlm.nih.gov/geo/query/acc.cgi?acc=GSM4859391] (H3K27me3). The Hi-C compartments track for HAP1 was downloaded from the 4D Nucleome Data Portal, under the accession code 4DNFIAXG3ZPL [https://data.4dnucleome.org/files/4DNFIAXG3ZPL/]. PhyloP scores from the 241 eutherian mammal Zoonomia alignment were used for conservation analysis, downloaded from UCSC [https://hgdownload.soe.ucsc.edu/goldenpath/hg38/cactus241way/]. GENCODE (release 44) [https://www.gencodegenes.org/human/release_44.html] was used for the location of protein-coding promoters. Bismap (downloaded from UCSC [https://hgdownload.soe.ucsc.edu/gbdb/hg38/hoffmanMappability/]) was used for the location of mappable regions. The consensus repeat sequences were downloaded from Dfam [https://www.dfam.org/]. Source Data are provided with this paper.

Research involving human participants, their data, or biological material

Policy information about studies with human participants or human data. See also policy information about sex, gender (identity/presentation), and sexual orientation and race, ethnicity and racism.

Reporting on sex and gender	N/A
Reporting on race, ethnicity, or other socially relevant groupings	N/A
Population characteristics	N/A
Recruitment	N/A
Ethics oversight	N/A

Note that full information on the approval of the study protocol must also be provided in the manuscript.

Field-specific reporting

Please select the one below that is the best fit for your research. If you are not sure, read the appropriate sections before making your selection.

☒ Life sciences ☐ Behavioural & social sciences ☐ Ecological, evolutionary & environmental sciences

For a reference copy of the document with all sections, see nature.com/documents/nr-reporting-summary-flat.pdf

Life sciences study design

All studies must disclose on these points even when the disclosure is negative.

Sample size	ChIP-seq experiments were performed on 315 TFs with previously uncharacterized binding specificities and 58 controls. This is part of the larger Codebook Consortium, with the primary purpose of assessing 332 uncharacterized and putative human TFs for sequence-specific DNA-binding activity and producing trustworthy motifs for them, in order to assemble a complete human TF motif collection. This is described in more detail in an accompanying manuscript (Jolma et al. 2026a).
Data exclusions	We describe a collection of 790 ChIP-seq experiments. We excluded 274 experiments from further analysis due to low peak numbers. For the remaining 516 experiments, we utilized expert curation to identify experiments that displayed consistent results across replicates and platforms. This expert curation system is described extensively in the accompanying manuscripts (Jolma et al. 2026a, Voronstov et al. 2025).
Replication	For 338 TFs (out of the entire 373, 91%), ChIP-seq was performed twice or more times using the same protein; 169 (50%) were successful in more than one replicate (153 in all replicates tested for that protein), 143 (42%) were unsuccessful in all experiments, and 26 (8%) yielded one successful and one or multiple unsuccessful experiments.

Randomization	ChIP-seq experiments were distributed across batches and experimenters to balance replicates, sequencing runs, TF DNA-binding domain subtypes, and control versus uncharacterized TFs. Proteins were assigned largely according to practical considerations such as protein availability and alphabetical order by gene/protein symbol. Formal randomization was not applicable because the study was designed as a systematic survey of DNA-binding activity across many putative TFs rather than as a comparison between randomized treatment groups. Systematic batching also minimized unnecessary reagent use and experimental handling, and reduced the risk of sample tracking errors or cross-contamination.
Blinding	Researchers were not blinded to transcription factor identity. Experiments and analyses were performed systematically in batches of many proteins, and the primary objective was to determine whether the analyzed putative transcription factors displayed sequence-specific DNA-binding activity. Blinding was not considered applicable because the study did not involve subjective assessment of outcomes or comparison between treatment groups, and the experimental readouts were analyzed systematically. Blinding would have also increased unnecessary reagent use and experimental handling, and increased the risk of sample tracking errors or cross-contamination.

Reporting for specific materials, systems and methods

We require information from authors about some types of materials, experimental systems and methods used in many studies. Here, indicate whether each material, system or method listed is relevant to your study. If you are not sure if a list item applies to your research, read the appropriate section before selecting a response.

Materials & experimental systems

n/a	Involved in the study	
☐	☒	Antibodies
☐	☒	Eukaryotic cell lines
☒	☐	Palaeontology and archaeology
☒	☐	Animals and other organisms
☒	☐	Clinical data
☒	☐	Dual use research of concern
☒	☐	Plants

Methods

n/a	Involved in the study	
☐	☒	ChIP-seq
☒	☐	Flow cytometry
☒	☐	MRI-based neuroimaging

Antibodies

Antibodies used	Anti-eGFP antibody (Ab290, Abcam) was used for TF ChIP-seq experiments, and anti-H3K9me3 (Ab8898, Abcam) was used for H3K9me3 ChIP-seq. Each ChIP-seq reaction used 2 µl of undiluted polyclonal antiserum, corresponding to 10 µg total IgG, immobilized on 60 µl of protein G magnetic bead suspension (Dynabead 10004D, ThermoFisher).
Validation	These are a common laboratory reagents, cited in over 3370 (Ab290) and 1730 (Ab8898) publications; see https://www.abcam.com/en-us/products/primary-antibodies/gfp-antibody-ab290 and https://www.abcam.com/en-us/products/primary-antibodies/histone-h3-tri-methyl-k9-antibody-chip-grade-ab8898 for more details.

Eukaryotic cell lines

Policy information about cell lines and Sex and Gender in Research

Cell line source(s)	HEK293 Flp-In T-REx cells 608 (Invitrogen)
Authentication	This Flp-In T-REx HEK293 cell line is a commonly-used laboratory cell line that contains a recombination site. Our cells are isogenic cell lines, and each transgenic cell line specifically expressed the specific TF that was inserted to the Flp site after doxycycline treatment (verified by EGFP expression), and further confirmed using the sequencing results. This cell line has many citations, many are listed on the Invitrogen web site.
Mycoplasma contamination	Cell line tested negative for mycoplasma by PCR.
Commonly misidentified lines (See ICLAC register)	HEK293 Flp-In T-REx cells are not listed as commonly misidentified cells.

Plants

Seed stocks	*Report on the source of all seed stocks or other plant material used. If applicable, state the seed stock centre and catalogue number. If plant specimens were collected from the field, describe the collection location, date and sampling procedures.*
Novel plant genotypes	*Describe the methods by which all novel plant genotypes were produced. This includes those generated by transgenic approaches, gene editing, chemical/radiation-based mutagenesis and hybridization. For transgenic lines, describe the transformation method, the number of independent lines analyzed and the generation upon which experiments were performed. For gene-edited lines, describe the editor used, the endogenous sequence targeted for editing, the targeting guide RNA sequence (if applicable) and how the editor was applied.*
Authentication	*Describe any authentication procedures for each seed stock used or novel genotype generated. Describe any experiments used to assess the effect of a mutation and, where applicable, how potential secondary effects (e.g. second site T-DNA insertions, mosiacism, off-target gene editing) were examined.*

ChIP-seq

Data deposition

☒ Confirm that both raw and final processed data have been deposited in a public database such as GEO.

☒ Confirm that you have deposited or provided access to graph files (e.g. BED files) for the called peaks.

Data access links<br>*May remain private before publication.*	The sequencing raw data for the ChIP-seq experiments have been deposited to the SRA database under identifier PRJEB78913. Genomic interval information generated for ChIP-seq has been deposited into GEO under the accession GSE280248.
Files in database submission	The list of all the files produced are available at Supplementary Data 2 and https://codebook.ccbr.utoronto.ca.
Genome browser session<br>(e.g. UCSC)	*Provide a link to an anonymized genome browser session for "Initial submission" and "Revised version" documents only, to enable peer review. Write "no longer applicable" for "Final submission" documents.*

Methodology

Replicates	For 338 TFs (out of the 373 total; 91%), ChIP-seq was performed two or more times using the same protein. Supplementary Data 2 outlines all replicated ChIP-seq experiments and their success status for all tested TFs.
Sequencing depth	20M (targeted)
Antibodies	Anti-eGFP antibody (Ab290, Abcam)
Peak calling parameters	MACS2 with sample-specific controls, using options --format BAM --gsize hs --pvalue=0.001 (described in detail in the manuscript).
Data quality	We used two criteria to determine which experiments were successful. First, the data were analyzed as part of a larger Codebook benchmarking effort, which is described in more detail in accompanying manuscripts (Voronstov et al. 2025, Jolma et al. 2026a). The Codebook benchmarking included expert curation that relied mainly on obtaining similar motifs for the same TF from different in vivo and in vitro data types (ChIP-seq, Protein Binding Microarrays, SMiLE-seq, and several variants of HT-SELEX) as evidence of direct, sequence-specific DNA binding. This Codebook motif benchmarking identified 130 Codebook TFs and 49 controls with successful, motif-enriched ChIP-seq data, meaning that sequence-specific DNA binding observed in ChIP-seq (in vivo) is supported in almost all cases by in vitro experimental data (Supplementary Data 1).<br>Second, we identified experiments in which the peak overlaps of biological replicates exceeded what is expected at random (i.e., with TF identities permuted). ChIP-seq experiments that were classified as successful based on the motif similarity analysis described above displayed a higher overlap between TF replicates relative to mismatched TFs (median Kulczynski II coefficient of 0.57 vs. 0.034; Supplementary Fig. 2a; this statistic is a modified Jaccard value that compensates for class imbalance). A Kulczynski II coefficient threshold of 0.4 captures 79% of approved experiments with replicates, and 90% of controls, but eliminates 94.5% of mismatched experiments; thus, we used this value as a threshold for success of replicate experiments, independent of motifs. By this measure, an additional 36 putative TFs (and two controls) were considered technically successful, despite not displaying motif enrichment (Supplementary Data 1 and 2). The DNA binding in these cases may be indirect, i.e., the proteins may be DNA-associated chromatin factors, rather than TFs. Alternatively, they may recognize properties of the DNA sequence that are not captured by common motif models. Nevertheless, we included these 38 TFs in subsequent analyses. Cautioning that these proteins may be chromatin factors, for simplicity, we refer to the entire set of 217 successful proteins (130 putative + 49 control TFs successful in the first round, and 36 putative + 2 controls with matching replicates; Supplementary Fig. 3a and Supplementary Data 3) as “TFs” in the text.
Software	MACS2
