# Supplementary material for: Extensive binding of poorly characterized human transcription factors to genomic dark matter
Source: Nat Commun. 2026 Aug 5;17:7770. doi: 10.1038/s41467-026-75376-z (PMC13444102; doi:10.1038/s41467-026-75376-z)
Supplement: Supplementary file 5 [file 41467_2026_75376_MOESM9_ESM.pdf]

nature portfolio

Peer Review File

Extensive binding of nebulous human transcription factors to genomic dark matter

Corresponding Author: Professor Timothy Hughes

This file contains all reviewer reports in order by version, followed by all author rebuttals in order by version.

Version 0:

Reviewer comments:

Reviewer #1

(Remarks to the Author)
Review Razavi et al.
In this study, the authors identify the genomic binding locations of 166 uncharacterized human TFs. They use the term 'Our genome's Dark Matter' and 'Dark TFs', and their binding sites, for TFs that bind to often non-conserved regions of our DNA, often containing many TE sequences. The emphasis of this manuscript lies with these Dark TFs, many of which are KRAB ZNF proteins (KZNFs). Overall the aim of the study is clear, the authors use ChIP-seq data to explore the binding patterns of many previously uncharacterized TFs in our genome. However, I have serious concerns about the validity of conclusions regarding the Dark TFs and about how 'uncharacterized' these TF really are.

Main comments:

I am very worried about a potential bias in the ChIP-peak calling parameters that might explain the high number of binding sites of many of the Dark TFs to the 'Dark Genome', and how this ends up contrasting to the set of TFs that bind to promoters and enhancers. In mapping of ChIP-seq data and the subsequent peak calling, one can expect that a certain TF with a strong DNA binding affinity to promoters and enhancers, would yield a peak set where the strongest peaks (to promoters/enhancers) are predominating. The presence of many strong peaks in the dataset may actually compromise the detection of occasions where this same TF binds weakly to other genomic regions: While promoter/enhancer peaks will predominate the ChIP-seq dataset of promoter/enhancer binding TFs, the occasional interaction of this same TF to the 'Dark Matter' or our genome may not reach the peak-calling thresholds. In contrast, a Dark-TF which does NOT have a strong affinity to any particular DNA sequence, may still bind to DNA in regions where there is just enough protein-DNA interaction for the weak TF to clamp on. In the absence of a set of strong peaks, the peak calling parameters could favor the peak calling of those many weak DNA interactions. The bias I am afraid is at play, is that the described feature of Dark TFs to bind to the 'Dark Matter' of our DNA may not at all be specific to Dark TFs: The same may occur with any over-expressed TF in a ChIP-seq setting (or in vivo), including promoter/enhancer interacting TFs, but due to the presence of much stronger interactions in the latter case, the weakest interactions may simply not reach the cutoff for peak-calling.

Indeed, the authors note in lines 324-328 that " many Promoter and Enhancer TFs have a greater tendency to bind "empty" and"heterochromatin" state DNA in vitro than in cells" which could be a confirmation of this ChIP-seq calling or detection bias described above: Promoter/Enhancer TFs can evenly well bind to these empty" and"heterochromatin"DNA elements, but these 'inferior' binding sites are simply not called in the ChIP-seq peak calling.

One other concern lies with the author's definition and classification of Dark TFs and the Dark genome; In a way, the analyses in this manuscript provides a clear argument that a big proportion of the idenfitied Dark TFs that recurrently bind to specific TE classes (Figure 5) may not actually qualify for this definition: They bind to clearly annotated genomic elements in our genome (namely specific TE classes) which makes them clearly different from other Dark TFs that bind to seemingly random and non-TE content of our genome. In a way, by determining the genome wide binding pattern of these uncharacterized TFs, the authors have opened up possibilities to provide a much better classification than what is provided now, and even though the terms 'Dark TFs' and the 'dark genome' may be catch, it may not be appropriate for many of the well-annotated KZNF Dark TFs that bind to equally well annotated TEs elements in our genome.

Finally, during the review of the manuscript, it became increasingly unclear what criteria were used to define a TFs as

uncharacterized. This was obviously the central aim of this study; to provide genome wide binding data for previously uncharacterized TFs: However, in the paragraph ‘Functions of Dark TFs’ and Figure 7, the authors raise serious doubts about how uncharacterized these TFs really are: Many of them have described functions, many hae known interactors, or have previously been associated with cellular functions. This is an important point, because the title and abstract raises sincere interest in a set of ‘166 uncharacterized human TFs’, for which the vast majority, as it appears at the end of the manuscript are not at all truly uncharacterized.

Other remarks:
In Figure 2B, it is not clear what it means that the Jaccard coefficient for most of the ‘Dark TFs’ are so low, and what It means that they are so much in contrast to the high values of manu promoter/enhancer binding TFs. Despite an elaborate description, the message of this figure, and the accompanying text remains unclear, much more clarification would be needed to make this accessible to a broad readership.

The text and figures are really complicated in language, and very bioinformatics-heavy, and certainly not accessible to the average Nat Communication reader.

(Remarks on code availability)
n/a

Reviewer #2

(Remarks to the Author)
The manuscript “Extensive binding of uncharacterized human transcription factors to genomic dark matter” by Razavi and colleagues describes an analysis of a subset of transcription factors assayed as part of the Codebook consortium. The subset that this paper focuses consists of factors that bind to regions of the genome with closed chromatin in HEK293 cells.

Overall, the approach and experimental and computational methods of the Codebook consortium are to be commended. The motivation for the project is based on our still strikingly incomplete knowledge of where human transcription factors bind and how they function within the interconnected system of genome biology. Experimental protocols, resulting data and the presented analysis seem to confirm accuracy and consistency. These data are valuable and will be highly used by other researchers working on these questions.

Unfortunately, as I reviewed this manuscript there are incredible gaps in the analysis and interpretation of the so-called Dark TFs that this manuscript is focused on. Many of these gaps are because the division of information in this manuscript and the accompanying Jolma, et al manuscript available on biorxiv is muddled and confused making it difficult to make sense oy the context of the results presented here. As a reviewer of this manuscript only and not of both of them, I will focus my comments on this work, but repeatedly include examples and comments on how the failure to adequately distinguish this work from the companion paper dramatically hampers this current paper. Several major comments elucidate these concerns.

1. Dark TFs are categorized as a set of 54 proteins where “most peaks” live outside of states annotated as promoter, enhancer, insulator or gene bodies and fewer than half overlap ATAC-seq. As the authors note with the examples of YY1, this category definition is somewhat arbitrary. At least from the point of view of promoter and enhancer designation, figure 2A does not provide a clear separation from the set of DNA binding proteins categorized as “other” and those categorized as Dark TFs (presumably this overlap results from the use of the ATAC-seq filter in figure 2B). Figure 2B also does not suggest that the author’s definition is a clear one. The TF categories presented by the authors are fundamentally arbitrary and this arbitrary nature affects all of the results in the manuscript. Unbiased clustering of the various characteristics of the binding regions represented by tracks at the top of figure 2B would be a more convincing way to create this definition than the one the authors use as would other, more simple, overlap strategies. Additionally and demonstrating the frustrating division of information between this manuscript and the Jolma manuscript, a plot of TF category with respect to age, DND binding type, and conservation is not found in this manuscript, but instead is in Jolma as figure S4, although the process leading to the “TF category” in that figure seems to be completely missing from the Jolma manuscript and only described here – perhaps the figure S4 is in the wrong manuscript?

2. The point of figure 3C is not clear. The promoter sites have, by definition, more that 37% of their binding sites in promoters. The point appears to be that the GHT-SELEX peaks and the ChIP-seq peaks are highly concordant. Similarly, figure 3E shows the effects of the same definition of Dark TFs – more than 50% of the binding site are defined to be in regions of empty or heterochromatin. Rather than presenting that a second data type designed to measure the same phenomenon sees the same definitional thresholds, more effective presentation would be a global comparison of correlation of the ChIP-seq and GHT-SELEX data (which may be the point of the Vorontsov, et al, companion paper currently on biorxiv).

3. Analysis of Dark TFs is incomplete. For example, the transcription factor ZNF698 is reported in this manuscript to have a “much smaller number of CTOP sites, and is enriched for binding L1M5 elements” (line 372) later quantified to “~50 conserved sites enriched for L1M5” (line 527) out of a total of 558 ZNF698 binding sites (figure 4A). In Jolma, we learn that ZNF698 is an example of a strongly conserved binding site in an L1ME1 transposon just over 100bp from a predicted enhancer with a CTCF binding site. The information presented make it challenging to get a full picture of the relevant biology here. The Jolma example of the ZNF698 binding must be an example of a clustered CTOP that is a major focus of the Jolma manuscript, but ignored here where ZNF698 binding sites are presented as not highly conserved. Obviously both of these things can be true, but this presentation is scattered and incomplete.

4. The discussion of conserved triple occupancy sites (CTOPs) is also confused between this manuscript and Jolma making it hard to follow and ultimately unsuccessfully presented because key results necessary for this manuscript to make sense are omitted. Specifically, in this manuscript, we are told that CTOPs exist for both promoter TFs and Dark TFs, but the number and fraction of CTOPs in promoters is much higher (line 357) along with figure 4 showing promoter TFs. We are told that CTOPs adjacent to other CTOPs are explored in Jolma (line 363). In Jolma, we are told that there are 121,785 CTOPs in the Codebook and control sets and that the "strong support for the functional importance of Codebook TF binding in the genome" are summarized in greater detail in this manuscript. Examples of critical information omitted from this manuscript include Jolma providing results about what fraction of CTOPs are variant free, although we have no information in this manuscript about the fractions of Dark TF CTOPs that are variant free, which would give information about function of these binding sites (which according to the note in Jolma quoted above this manuscript is supposed to provide). We also have apparently no results presented here about how often CTOPs for Dark TFs cluster even though this clustering is considered in Jolma to be "indicating a likely biochemical function for these elements". The clustering of CTOPs and the relation of these to function are important enough in Jolma to take up nearly one page of text and most of one figure.

In summary, read in concert with the Jolma, et al manuscript and the Vorontsov, et al manuscript on bioxriv, the distinct feeling this reviewer gets is a reminder of the ENCODE pilot project publication effort in 2006 and 2007 where the consortium members divided the work into five publications that were overlapping, incomplete and fundamentally not particularly interesting on their own. The Nature editors wisely forced ENCODE to combine the results into a single paper (on which all five author lists remain) that become a seminal description of integrated genome biology. I believe a similar approach is appropriate here.

(Remarks on code availability)

Reviewer #3

(Remarks to the Author)
Razavi et al. aim to better identify the binding motifs and sites of uncharacterized transcription factors (TFs) as part of a larger initiative called the "Codebook". They utilize various methodologies to measure TF occupancy in the genome. This represents a significant amount of work employing experimentally-sound methodologies, such as ChIP-seq, along with rigorous analytical processing. Multiple experiments were conducted with replicates across several cell lines by different researchers, focusing on both known and unknown TFs. They utilized the flp-in HEK293T cell line for genome-integrated, doxycycline (DOX)-induced overexpression of all TFs, confirmed by a GFP tag and immunoprecipitation.

The authors were very stringent in selecting binding sites for further analysis. They began with curation based on reliable motifs identified across multiple techniques used in previous studies. Next, they focused on TFs that exhibited consistently overlapping peaks between ChIP-seq replicates from this study to classify them according to their genome binding locations. Further filtering of the ChIP peaks involved overlapping them with the in vitro technique GHT-SELEX (conducted for another study) and assessing whether there is a motif match at the peak location. The inclusion of GHT-SELEX enhanced their confidence in identifying motifs bound by these uncharacterized TFs, which is a major goal for the authors. In their discussion, the authors first address the conservation of TF-binding sites and their evolutionary age while comparing "promoter" and "dark" TFs. Ultimately, they leverage their data alongside existing literature to provide insights into the function of TF binding in the dark genome, beyond merely silencing transposable elements.

Overall, this manuscript is well written, and the data support their conclusions. However, the study lacks functional validations of some of their interpretations, such as the importance of SOX2 binding to the dark genome in relation to its pioneering or reprogramming ability. Moreover, the authors highlight that their focus is limited to the identification of TF binding sites and not how this binding is linked to TF function. Nevertheless, I believe the extensive data released in this study will be highly beneficial for the field investigating TF binding patterns.

Minor Corrections:
- At times, I found it confusing to identify which TFs or peaks the authors were analyzing for each figure. A supplementary figure that explains the progressive filtering of proteins and peaks, indicating which are used in each main figure of the paper, would improve clarity.

- In bubble/scatter plots (e.g., Fig. 2A or Fig. 3A-E) and lists of TFs (e.g., Fig. 3F and Fig. 5A), it would be informative for the reader to distinguish between control and Codebook TFs.

- In Fig. 3A and B, I suggest changing the y-axis label to "TOPs" instead of "specific binding," as this is how they are referred to in the text.

- Since the authors focus significantly on the age and evolution of TF-binding sites, should this be mentioned earlier in the abstract and introduction to attract the attention of potential readers?

- Regarding lines 397-398, Figure 5A: If these are indeed the TOP sites, then the strong correlation between ChIP and GHT-SELEX is expected, as they have filtered each other already.

(Remarks on code availability)

Reviewer #4

(Remarks to the Author)
In this manuscript, the authors report novel analyses on the genomic binding preferences and sequence specificity of many uncharacterised transcription factors (TFs). Their findings are based on substantial ChIP-sequencing datasets obtained in HEK293 cells upon inducible expression of a set comprising 315 uncharacterised TFs and 58 control TFs.

Following quality control of these data, the authors report genomic binding preferences across promoter, enhancer and heterochromatin genomic regions (defined via canonical histone mark enrichment). This allows them to define subsets of uncharacterised transcription factors preferentially binding promoters, enhancers or “dark” heterochromatin in HEK293 cells; however the rest of the study largely focuses on TFs binding preferentially to promoters or heterochromatin (“dark TFs”). The manuscript next focuses on investigating the relationship between in vivo and in vitro binding preferences across the two classes, examining conservation patterns of sequence motifs within in vivo binding sites, and characterising the association of genomic binding and sequence specificity with repetitive elements (in particular for “dark” TFs) and the evolutionary age of binding sequences and transcription factor proteins. Lastly, the authors propose candidate functions on transcriptional repression across “dark” TFs with genomic binding to heterochromatin regions, based on available literature evidence and genomic, proteomic and epigenomic data.

Overall, this is a very interesting study reporting a novel resource to explore the biochemical signatures and potential roles of a large number of previously uncharacterised proteins with predicted TF function. The authors' results are likely to be significance to a wide readership, given the novelty of the interrogated proteins and the scope of the presented analyses. However, in its present form we have some concerns on the clarity of presentation in some areas, and the contextualisation of observations and interpretations to the author’s experimental system and supporting data.

Major concerns:

1) Although some details are included in the manuscript, the authors should be clearer on how ectopic expression of TFs in their HEK293 experiments compares with endogenous TF binding in previous ChIP-seq datasets. To the author’s credit, their design includes 58 control transcription factors. Incorporating a more detailed comparison of their HEK293 data for these proteins versus available endogenous datasets would be helpful to provide a benchmark for interpretation of results on their uncharacterised putative TFs. For instance, from the data presented it is not clear whether genomic binding of control TFs (e.g. CTCF or YY1) significantly differs from endogenous datasets (whether in the same regulatory context i.e. HEK293 cells or in different cell lines or tissues). There is substantial evidence in the literature for promiscuous TF binding in ChIP-seq data upon ectopic TF expression, and the authors should address this potential caveat with their control datasets.

More clarity on the above may also help interpret some of the results in the manuscript. For instance, the observed low number of novel TFs with preferential binding to enhancer elements may be partly explained by ectopic expression of transcription factors in a single cellular and chromatin context (which could be compatible with the authors observation on the ‘GeneHancer’ set, lines 209-213). For control TFs, are genomic binding preferences across promoters and enhancers similar between the author's ectopic data in HEK293 and various endogenous contexts across previous datasets?

2) On a similar note, the author’s observations in Figure 2 regarding much lower correlations in binding positions across “dark” TFs compared to promoter TFs should be supported with more detailed analyses. As the author’s rightly point out, heterochromatin can be systematically depleted from ChIP-seq experiments due to sonication biases, which could partly explain the author’s observations. This caveat could also suggest ChIP-seq data for heterochromatin-binding TFs may have systematic experimental biases, such as lower signal to noise or a lower number of reproducible peaks. To ensure the data can be interpreted accurately, the authors should incorporate analyses explicitly comparing metrics such as number of reproducible peaks, genome coverage across all peaks and fractions of reads in peaks across the three TF classes they define. For instance, does data for “dark” TFs typically yield lower numbers or reproducible peaks, or peaks that cover a smaller fraction of the genome? Some of these data is available in the supplemental tables, but is not explicitly annotated or compared (for instance, a TF class column for promoter, enhancer, dark or other should be added to Table S3).

3) The authors should consider a more careful use of the term “functional” across the manuscript, especially in its current context as no experimental validations are presented. As the authors mention in the discussion, the relationship between biological function and evolutionary turnover is debated in the context of gene regulation. In the later figures of the manuscript, the authors are also using the term “functional” repeatedly to describe supporting literature evidence (which can be said to be limited, as these are largely uncharacterised TFs) and proteomic, genomic and epigenomic annotation/data. A more cautious presentation of these analyses and their conclusions would help contextualise these results as largely based on biochemical readouts (rather than experimental validation experiments). Indeed, incorporating a few targeted validations in Figure 7 (for instance by measuring changes in heterochromatin following expression of candidate “dark” TFs) would have strengthened the authors’ results.

Minor comments:

- The methods are presented succinctly and, on occasion, in a convoluted manner. For instance, the section on ChIP-seq

peak calling and the author's use of input controls is difficult to follow as presented. Have the author's used input controls matched to experiments for each TF (i.e. in a way that inputs correct for experimental batches and variables such as cells passage number, days under selection, etc)? Or is their approach rather based on use of a more limited set of input experiments and a "consensus" set of background control regions, in a manner similar to greylisting? From the current description in lines 640-661 it is not clear which of the two approaches above is conceptually closer to the author's method (although details in Table S2 seems to suggest inputs are not matched for each TF's cell line). The authors should consider carefully revising the methods for clarity and detail, and expand if needed (there do not seem to be supplementary methods elsewhere in the manuscript).

- It is not clear from the manuscript how the candidate 315 uncharacterised TFs have been selected with regards to their tissue-specific gene expression. Are properties such as tissue-specific expression scores different across the classes of TFs the authors define (e.g. in Figure 2A)? Similar to comments above, the authors may want to incorporate supporting analyses in this area, for instance using human GTEX data.

- Being clearer across the manuscript about the numbers of TFs represented in the various analyses would add clarity to the authors' data presentation. As far as we can tell, the authors are presenting 217 TFs in Figure 2 and 137 TFs in Figures 3 and 4. This can be gleaned from the text but could be clearer in the figures themselves and their legends. In later figures this information could be made for explicit (e.g it seems less than 317 TFs are selected for Figure 5, but it is not clear what specific numbers are included in this analyses).

- The data presentation in some of the figure panels could be made clearer. Some examples:
- The difference in Figure 2B are very difficult to see with the current shading and magnification. The authors could consider using a white background for this plot, and/or including separate correlation plots for Promoter&Enhancer vs Dark TFs
- The right hand-side panel in Figure 3F does not clearly relate to the presentation of this figure in the associated results section, in which CTOPs have not been yet introduced as a concept. The authors could consider removing this panel for clarity (also given the following figure focuses on CTOPs).
- The panels in Figure 5A are very dense and difficult to follow. For clarity, the authors may consider tweaking the data presentation here.

- The authors may need to clarify any limitations in their ability to map TF binding to repetitive elements in the reported ChIP-seq data (especially for young repeat families with many identical or near-identical copies across the human genome). This limitation may not apply to the specific examples highlighted in figures, but even then would be useful to comment on for clarity.

- The analyses presented in Figures 6B and 6C suggest the combined information on binding sites age (6B) and TF sequence age (6C) may discriminate between promoter and dark TFs. The authors may want to explore this possibility and specify whether this is the case.

- Referencing in the discussion section is rather sparse and should be revised.

(Remarks on code availability)

Reviewer #6

(Remarks to the Author)
I co-reviewed this manuscript with one of the reviewers who provided the listed reports. This is part of the Nature Communications initiative to facilitate training in peer review and to provide appropriate recognition for Early Career Researchers who co-review manuscripts.

(Remarks on code availability)

Version 1:

Reviewer comments:

Reviewer #1

(Remarks to the Author)
The authors have addressed all of my concerns and questions, and added new data to substantiate their interpretations and conclusions. The manuscript has significantly improved and I am pleased to see the current form of manuscript, including the title and abstract, which are more appropriately describing the TFs that are part of their identification and profiling. I have seen the other reviewers raised similar and novel comments, that I believe were handled well by the authors as well.

(Remarks on code availability)

Reviewer #2

(Remarks to the Author)
The authors have revised their manuscript in response to my comments and those of the other reviewers. Although the manuscript is improved especially in way that the results are self-contained and not excessively reliant on cross-reference with other manuscripts, there are still a number of concerns that limit the impact of this work.

Major comments:

1. The inclusion of unbiased clustering of the TF binding features in supplementary figure 6 is appreciated. Although partly reassuring that the overlap between the manual classification and the clustered one does not include any classified dark TF clustered with classified promoter TFs (or vice versa), the author's assertion in the rebuttal that their manual classification "produced more stringent results" and "removes TFs in which the classifications are less clear" is without any presented evidence in the rebuttal or the manuscript. In fact, based on the clustering in many cases they failed to manually classify TFs that cluster extremely closely with TFs that they do manually classify. This effect is most striking for dark TFs, but occurs in all categories. Additionally, the authors describe the clustering results as "very similar" to their manual classification, when in fact 32% (69/127) factors change classification.
For example, only ~60% of the TF that cluster into the dark category are classified as dark TFs for the subsequent analysis. Considering the general focus in the manuscript on global characteristics of dark transcription factors, this feels like either a bias or a missed opportunity.
Specifically, the authors make the point that the set of dark TFs has a number of fundamentally different characteristics compared to the set of promoter TFs and this is true whether the manual or clustered sets are chosen. However, nearly all of their claims about the characteristics of the dark TFs seem to be considerably less trustworthy given the large number of TFs (including many than apparently cluster very closely) that they have manually excluded from the analysis.
It is, of course, a challenge to create a sensible partition of the transcription factors to anchor the various analysis in this paper. However, the process used here dramatically limits general statements that can be made. If the authors continue with their manual classification, they must provide both a more complete justification, potentially with a workflow, to describe the "meaningful large-scale trends" that they used to create the set. Their current approach appears to be ad hoc either because it is or because they have made classification decisions that are not transparently presented.

2. The effort to better organize this paper and separate it from other papers of the Codebook consortium is appreciated. The revisions and the change in the order the results are presented have made significant progress here and the rebuttal provides more clarity about what the authors are trying to do in this manuscript and in the companion manuscripts. Specifically, the authors are clear that they are not trying to completely characterize individual transcription factors as aspects of the biology of a given factor may be split between their analysis manuscripts. Instead, they are trying to make general statements about their set of dark TFs and provide examples that illustrate this point. This is a valid approach likely to have the most impact.
Following this, once the authors commit to focus on a comparison between promoter and dark TFs (page 9 lines 315-316), they should strongly consider omitting the "other" transcription factors from the main text figures unless necessary for very specific and limited points. This is especially true for figure 3b where the inclusion of the "other" factors muddies the point of this panel making an already difficult figure harder to understand. Of course, one reason that almost 40% of the factors presented in figure 3b are classified as "other" (17/45) is that they are misclassified and actually cluster with the dark transcription factors, potentially suggesting again that their manual classification scheme seems to have considerable problems in reflecting transcription factor biology that the manuscript is attempting to described and is potentially inappropriate.

In sum, this manuscript describes a potentially very important dataset, but one that has seemingly been analyzed incoherently based on a manual classification that the author's own results seem to show is poorly justified and potentially problematic. This flaw substantially mars the results.

Other comments:

1. In the first paragraph of the introduction, the authors state that "function of genomic dark matter in gene regulation is also supported by the existence of TFs that are capable of binding TE and ERE sequences" – this is not logically correct. The existence of DNA binding proteins that bind TE and ERE sequences is evidence that these DBPs are functional and that their function is to bind and/or silence TE and ELE elements. The binding itself is not evidence that these regions participate in gene regulation.

2. The definition of a conserved TOP as having at least one base pair with an FDR corrected phyloP score greater than one is appropriate for setting a threshold on a set of TOPs to analyze for confirmation. However, the suggestion that "the corresponding TFs have conserved functions at promoters" or other binding sites (page 12, lines 449-455) is not supported by the data with these thresholds. The is a comment to soften the language of functional claims rather than challenging the CTOP analysis.

3. Jolma, et al is listed twice in the references (35 and 48)

4. TOP sites are originally defined as "triple overlap" sites, but sometimes called "triple optimized overlap" sites. There are also occasionally references to "optimized thresholds". In the supplementary material there are "triple peaks" which is clearly something different from TOPs. This terminology confusion may be a remnant of addressing my previous comments about

the overlap and confusion between this manuscript and Jolma, et al. Regardless of the cause, the triple and TOP terminology should be thoroughly reviewed throughout the manuscript to ensure consistency. This is especially important because the characteristics of the TOP sites are a major part of this manuscript.

5. Figure 7 has different annotations on the top (DBD category) and in the legend (TF family).

6. The figure style is figures 4e, 4f, 5b, 5c, S9b and S9c is misleading. For all of these figures, the x axis is simply an individual transcription factor ordered by the value on the y axis. There is no connection between the factors, so it is inappropriate to connect the plotted values with lines. These data may be more appropriately plotted by box plots or in separate panels for promoters and dark TFs (i.e. each current panel becomes two panels). If the authors feel that there is explanatory value in using shapes to annotate controls and codebook TFs, the latter may be the best option. At a minimum, the plot should be remade without the connecting lines.

6. More generally, as mentioned indirectly in my comments and directly by one of the other reviewers, both the manuscript text and figures are often complicated, dense and difficult to parse. The revision has partly addressed this, but much of the manuscript remains difficult.
Many of the main text figures seem to have as their main message “we have a lot of data” and should be presented in more straightforward ways within the main text to highlight specific results and/or key differences between the promoter and dark TF sets while putting the complete figure into the supplementary material. Figure 3 is especially difficult to make sense of as are figure 2b, figure 4d and figure 7. Simply put, one main text figure early in the manuscript is sufficient to make the claim that there is a lot of data, other figures comparing specific differences between promoter and dark TFs (i.e. not many differences simultaneously as in the impossible to parse figure 3a). Finally figures with individual TF results where figure 5 and figure 6 are both already excellent in this regard.

7. Finally, it is true that many researchers have not read the nearly 25-year old Lander et al. paper in its entirety. It remains to be seen whether the work described in this manuscript is equivalent to sequencing the human genome.

(Remarks on code availability)

Reviewer #3

(Remarks to the Author)
The authors have addressed all my concerns and I fully support the publication of the manuscript in Nature communications.

(Remarks on code availability)

Reviewer #4

(Remarks to the Author)
The authors have performed a careful revision of the manuscript that addresses most of our comments (and those from the other reviewers). Their revision includes the addition of new data and analyses, and results in a much improved manuscript. It was particularly nice to see they now have incorporated some validation experiments and improved the narrative presentation of the study.

We have some minor comments regarding data presentation and interpretation that the authors are encouraged to consider:

- Some of the data presentation in the main figures could be improved to draw the attention of readers to key observations in the author’s analyses, especially for some of the more complex plots. An example is Figure 5a (phyloP scores across TOP sites for two promoter and two dark TFs). It seems the key observation in these analyses is the signature of selective constraint on key positions in the PWM for these TFs, yet this is somewhat buried in a complex plot that also shows “background” signal in a large window of surrounding sequence and unconserved sites. The authors should consider expanding the data around the motif logo for each TFs’ conserved sites, which would improve readability of the plot and draw the reader’s attention to this key observation. A similar concept could be applied to key TF/repeat pairs in Figure 3b.

- The data is Figure 6 could be made more compelling by addition of a graphical diagram outlining the experimental design for these experiments (as described in the results). We think this is really interesting new data, and its impact is somewhat diminished by the current presentation.

- The discussion could be streamlined and referenced in a more rigorous manner, especially for the last two paragraphs. Some of the discussion about the relationship between evolutionary conservation and TF function reads somewhat oversimplified, including with regards to the relative age of TFs and their binding sites. It should also reference key work in this area.

- Similarly, the discussion should mention some of the key limitations of the study. A surprising omission in this regard is the fact the author’s data is based on (ectopic) ChIP-seq profiling in a single human cell line, and therefore in a very specific chromatin and regulatory context. The authors should discuss in detail how this caveat contextualises their observations,

including on the applicability of the ChIP-seq profiles they report to other human cell types or tissues.

- The data annotation in some of the supplementary figures can be improved for readability (e.g. left axis on supplementary figure 8 for the bottom heatmaps, which could be clearly annotated as in supplementary figure 12).

(Remarks on code availability)

Reviewer #6

(Remarks to the Author)
I co-reviewed this manuscript with one of the reviewers who provided the listed reports. This is part of the Nature Communications initiative to facilitate training in peer review and to provide appropriate recognition for Early Career Researchers who co-review manuscripts.

(Remarks on code availability)

Open Access This Peer Review File is licensed under a Creative Commons Attribution 4.0 International License, which permits use, sharing, adaptation, distribution and reproduction in any medium or format, as long as you give appropriate credit to the original author(s) and the source, provide a link to the Creative Commons license, and indicate if changes were made.
In cases where reviewers are anonymous, credit should be given to 'Anonymous Referee' and the source.
The images or other third party material in this Peer Review File are included in the article's Creative Commons license, unless indicated otherwise in a credit line to the material. If material is not included in the article's Creative Commons license and your intended use is not permitted by statutory regulation or exceeds the permitted use, you will need to obtain permission directly from the copyright holder.
To view a copy of this license, visit https://creativecommons.org/licenses/by/4.0/

We are grateful for the careful consideration and thoughtful feedback provided by all the reviewers. Our responses to each reviewers' points are given in blue.

Reviewer #1 (Remarks to the Author):

Review Razavi et al.
In this study, the authors identify the genomic binding locations of 166 uncharacterized human TFs. They use the term 'Our genome's Dark Matter' and 'Dark TFs', and their binding sites, for TFs that bind to often non-conserved regions of our DNA, often containing many TE sequences. The emphasis of this manuscript lies with these Dark TFs, many of which are KRAB ZNF proteins (KZNFs). Overall the aim of the study is clear, the authors use ChIP-seq data to explore the binding patterns of many previously uncharacterized TFs in our genome. However, I have serious concerns about the validity of conclusions regarding the Dark TFs and about how 'uncharacterized' these TF really are.

Main comments:

I am very worried about a potential bias in the ChIP-peak calling parameters that might explain the high number of binding sites of many of the Dark TFs to the 'Dark Genome', and how this ends up contrasting to the set of TFs that bind to promoters and enhancers. In mapping of ChIP-seq data and the subsequent peak calling, one can expect that a certain TF with a strong DNA binding affinity to promoters and enhancers, would yield a peak set where the strongest peaks (to promoters/enhancers) are predominating. The presence of many strong peaks in the dataset may actually compromise the detection of occasions where this same TF binds weakly to other genomic regions: While promoter/enhancer peaks will predominate the ChIP-seq dataset of promoter/enhancer binding TFs, the occasional interaction of this same TF to the 'Dark Matter' or our genome may not reach the peak-calling thresholds. In contrast, a Dark-TF which does NOT have a strong affinity to any particular DNA sequence, may still bind to DNA in regions where there is just enough protein-DNA interaction for the weak TF to clamp on. In the absence of a set of strong peaks, the peak calling parameters could favor the peak calling of those many weak DNA interactions. The bias I am afraid is at play, is that the described feature of Dark TFs to bind to the 'Dark Matter' of our DNA may not at all be specific to Dark TFs: The same may occur with any over-expressed TF in a ChIP-seq setting (or in vivo), including promoter/enhancer interacting TFs, but due to the presence of much stronger interactions in the latter case, the weakest interactions may simply not reach the cutoff for peak-calling.

Indeed, the authors note in lines 324-328 that " many Promoter and Enhancer TFs have a greater tendency to bind "empty" and "heterochromatin" state DNA in vitro than in cells" which could be a confirmation of this ChIP-seq calling or detection bias described above: Promoter/Enhancer TFs can evenly well bind to these empty" and

"heterochromatin" DNA elements, but these 'inferior' binding sites are simply not called in the ChIP-seq peak calling.

If we are understanding correctly, the concern of the reviewer is that the binding of what we call "Dark TFs" to the empty and heterochromatin regions of the genome could be explained as poor binding or lack of specificity of these TFs, resulting in peak calls in what are essentially random locations. A related notion is that the promoter/enhancer binding TFs are not observed binding the empty/heterochromatin regions mainly because the signal in the assay is dominated by strong binding to promoters and enhancers, so the impact of noisy, low-level binding is obscured. The reviewer also brings in the issue that overexpression of TFs could skew the results.

Several lines of evidence indicate that the prevalence of peaks for Dark TFs in empty and heterochromatin regions represent *bona fide*, sequence-specific binding sites, and not technical artifacts or non-specific binding:

(1) We employed an industry-standard peak calling algorithm (MACS 2.0), which uses a local window for peak calling (<10 kb); thus, peaks in one region should not be impacted by peaks in other regions of the genome. Moreover, the peak calling threshold in the initial analyses that identified the Dark TFs was $P<10^{-10}$. This is thought to be a stringent cutoff, and the peaks typically have strong enrichment values (see **Supplementary Fig. 7c**; data for all peaks is found in GEO entry GSE280248).

(2) The Fraction of Reads in Peaks (FRiP) scores for the Promoter TFs and Dark TFs are similar, indicating that the Dark TF binding sites are not "inferior" at the level of the primary data. The newly added **Supplementary Fig. 7a** shows histograms of these FRiP scores. For both Promoter and Dark TFs, at $P<10^{-10}$ peak calling cutoff, more than 75% of reads are not within peaks (and with one exception (CXXC4), all promoter TFs have >85% of their ChIP-seq reads outside promoters (**Supplementary Fig. 7b**). This is a consistent trend; at a more liberal cut off threshold of $P<10^{-5}$ more than 60% of reads are outside of the peak regions for both Promoter and Dark TFs. Thus, the Promoter TFs large number of non-peak reads would have the same opportunity as the Dark TFs to form peaks in random regions of the genome.

(3) There is strong agreement between replicate peaks sets for the same Dark TF. To further support the non-random interaction of empty/heterochromatin DNA with Dark TFs, we show high peak overlap between biological replicates of the same Dark TFs, but not between mismatched Dark TFs, as shown in **Supplementary Fig. 2a-c**.

(4) Dark TF binding is sequence-specific. Dark TFs typically bind specifically to many of the same sites *in vitro* (GHT-SELEX) that they bind in ChIP-seq (**Fig.**

3a), and these sites typically correspond to motif matches derived from in vitro selection experiments. The simplest explanation for these observations is that these sites are driven by direct binding of the TF.

(5) For TFs with published ChIP-seq data utilizing antibodies against native proteins, there is strong overlap between our peaks and those in the published studies. Briefly, in the revised paper, we compared our peak sets for four control TFs (CTCF, YY1, MAX, GABPA) with at least two external (i.e. previously published by another group) ChIP-seq data sets performed against the endogenously-expressed TF in HEK293, K562, or HepG2. The Jaccard similarity score for peak overlap between our data set and the other data sets (i.e. Codebook vs. GTRD) is comparable and often exceeds the Jaccard score between different endogenous experiments. The new **Supplementary Fig. 1** shows the similarity score at a range of peak numbers, illustrating that the trend is independent of thresholds.

(6) More generally, we note that the peak numbers we obtain are within the same range as generally reported for antibody-based ChIP-seq, at the same cutoffs (median 12,200, mean 18,650). Thus, heterologous expression does not appear to produce an unusually large number of binding sites.

(7) The fact that *in vitro*-detected binding sites of Promoter TFs within empty/heterochromatin regions are not observed in cells can be explained by any of several plausible mechanisms: (i) ChIP-seq is biased towards open chromatin; (ii) truly preferential binding of a TF to open chromatin, (iii) lack of ability of a TF to access closed chromatin; (iv) indirect binding via cofactors present at promoters. This is now noted in the manuscript (lines 389-394).

One other concern lies with the author's definition and classification of Dark TFs and the Dark genome; In a way, the analyses in this manuscript provides a clear argument that a big proportion of the idenfitied Dark TFs that recurrently bind to specific TE classes (Figure 5) may not actually qualify for this definition: They bind to clearly annotated genomic elements in our genome (namely specific TE classes) which makes them clearly different from other Dark TFs that bind to seemingly random and non-TE content of our genome. In a way, by determining the genome wide binding pattern of these uncharacterized TFs, the authors have opened up possibilities to provide a much better classification than what is provided now, and even though the terms 'Dark TFs' and the 'dark genome' may be catch, it may not be appropriate for many of the well-annotated KZNF Dark TFs that bind to equally well annotated TEs elements in our genome.

In defence of what we acknowledge is an intentionally catchy monicker, the Dark Matter Genome is a term that is widely used, was not coined by us, and does typically include repetitive sequences and TEs (e.g. PMID: 33782602).

Still, the reviewer is making an excellent point – highlighting how our current data provides an unprecedented opportunity for better annotation and potential functions of much of the genome known so far as the dark matter DNA. Of course, these regions have already been labelled by Repeatmasker as repetitive elements; we are identifying proteins that bind the specific known sub-classes. We are therefore hesitant to incorporate new terms and classifications when the existing labels appear sufficient.

This comment, and other reviewer concerns, also prompted us to rewrite much of the Introduction to focus more on genomic dark matter, which we now realize had not previously been defined.

Finally, during the review of the manuscript, it became increasingly unclear what criteria were used to define a TFs as uncharacterized. This was obviously the central aim of this study; to provide genome wide binding data for previously uncharacterized TFs: However, in the paragraph 'Functions of Dark TFs' and Figure 7, the authors raise serious doubts about how uncharacterized these TFs really are: Many of them have described functions, many hae known interactors, or have previously been associated with cellular functions. This is an important point, because the title and abstract raises sincere interest in a set of '166 uncharacterized human TFs', for which the vast majority, as it appears at the end of the manuscript are not at all truly uncharacterized.

"TFs and TF-like proteins with no known PWM model in 2018" would be the literal description of how we chose proteins included in the study – this is explained at the beginning of the Results. "Uncharacterized" seemed a simpler term. It admittedly does not encompass information that has emerged since 2018, however, nor does it capture e.g. protein-protein interactions that have been previously described, or activities that could be reasonably inferred from the protein domain structure.

We now employ the term "nebulous", which encompasses the fact that aspects of the functions of these TFs were previously unclear, particularly their DNA-binding activity and associated genomic loci.

Other remarks:
In Figure 2B, it is not clear what it means that the Jaccard coefficient for most of the 'Dark TFs' are so low, and what It means that they are so much in contrast to the high values of manu promoter/enhancer binding TFs. Despite an elaborate description, the message of this figure, and the accompanying text remains unclear, much more clarification would be needed to make this accessible to a broad readership.

We have clarified the description of **Fig. 2b** in the manuscript (lines 248-250):

"**Fig. 2b** (bottom) shows a symmetric heatmap of Jaccard values (intersection/union) between the peak sets of the 217 successful TFs, providing an overview of similarities in genomic localization."

In other words, the Jaccard heatmap in **Fig. 2b** illustrates the similarity between the binding sites of individual TF pairs. A higher value for a TF pair indicates that the TFs bind to an overlapping set of locations, as is the case for many of the Promoter TFs (bright square in the lower right part of this heatmap). i.e., two Promoter TFs will often bind an overlapping set of promoters. A lower value for a TF pair, on the other hand, indicates that the TFs bind to different (i.e. non-overlapping) genomic locations, as is the case for most of the Dark TFs. This is also mentioned in the manuscript (lines 267-269):

“A second main feature of **Fig. 2b** is the diagonal line in the upper left quadrant of the heatmap. These are cases in which each peak set has very little overlap with any other peak set.”

The text and figures are really complicated in language, and very bioinformatics-heavy, and certainly not accessible to the average Nat Communication reader.

We have revised the text for simplification where possible and reviewed and rewritten the figure legends to ensure they can be understood by a non-computational biologist.

We have also added **Supplementary Fig. 3 (a-c)** which provides a “roadmap” of the study and definitions that are used in this manuscript for further clarification.

It is true that the paper is bioinformatics-heavy, but we believe it will be accessible to a general audience.

Reviewer #1 (Remarks on code availability):

n/a

Reviewer #2 (Remarks to the Author):

The manuscript “Extensive binding of uncharacterized human transcription factors to genomic dark matter” by Razavi and colleagues describes an analysis of a subset of transcription factors assayed as part of the Codebook consortium. The subset that this paper focuses consists of factors that bind to regions of the genome with closed chromatin in HEK293 cells.

Overall, the approach and experimental and computational methods of the Codebook consortium are to be commended. The motivation for the project is based on our still strikingly incomplete knowledge of where human transcription factors bind and how they function within the interconnected system of genome biology. Experimental protocols,

resulting data and the presented analysis seem to confirm accuracy and consistency. These data are valuable and will be highly used by other researchers working on these questions.

Unfortunately, as I reviewed this manuscript there are incredible gaps in the analysis and interpretation of the so-called Dark TFs that this manuscript is focused on. Many of these gaps are because the division of information in this manuscript and the accompanying Jolma, et al manuscript available on biorxiv is muddled and confused making it difficult to make sense oy the context of the results presented here. As a reviewer of this manuscript only and not of both of them, I will focus my comments on this work, but repeatedly include examples and comments on how the failure to adequately distinguish this work from the companion paper dramatically hampers this current paper. Several major comments elucidate these concerns.

1. Dark TFs are categorized as a set of 54 proteins where “most peaks” live outside of states annotated as promoter, enhancer, insulator or gene bodies and fewer than half overlap ATAC-seq. As the authors note with the examples of YY1, this category definition is somewhat arbitrary. At least from the point of view of promoter and enhancer designation, figure 2A does not provide a clear separation from the set of DNA binding proteins categorized as “other” and those categorized as Dark TFs (presumably this overlap results from the use of the ATAC-seq filter in figure 2B). Figure 2B also does not suggest that the author’s definition is a clear one. The TF categories presented by the authors are fundamentally arbitrary and this arbitrary nature affects all of the results in the manuscript. Unbiased clustering of the various characteristics of the binding regions represented by tracks at the top of figure 2B would be a more convincing way to create this definition than the one the authors use as would other, more simple, overlap strategies.

As requested by the reviewer, we now provide an unbiased clustering using the tracks at the top of **Fig. 2b** (**Supplementary Fig. 6**), which shows very similar results to our original thresholds.

It is true that our thresholds and criteria were selected manually, but not arbitrarily; we were attempting to capture what appeared to be meaningful large-scale trends within the dataset. It appears from the analysis in **Supplementary Fig. 6** that application of our manually-derived discriminating features mainly produces more stringent results than the clustering. i.e. it removes TFs in which the classifications are less clear.

The paper does point out (lines 285-289) that there are few monolithic groups:

“We sought to gain a better understanding of the properties and characteristics of TFs (and their binding sites) that represent the major patterns shown in **Fig. 2**. As a starting point, we defined four mutually exclusive groups (see **Supplementary Table 3** for TF

labels) based on the dominant annotation types that overlap with their genomic binding sites (i.e. ChIP-seq peaks)."

The "other" category, as elaborated in the Discussion, in particular contains more heterogeneous binding site features than Enhancer/Promoter/Dark TF categories.

Overall, while categorization of regulatory sites is clearly instructive, any classification scheme is likely to be more useful for interpretation by humans than it is in describing in detail the activities of TFs and regulatory sites, given that they are all unique, and these classifications are almost never absolute.

Nonetheless, the Promoter TFs and Dark TFs are clearly very different from each other, and are associated with the specific properties that we used to define them.

Additionally and demonstrating the frustrating division of information between this manuscript and the Jolma manuscript, a plot of TF category with respect to age, DND binding type, and conservation is not found in this manuscript, but instead is in Jolma as figure S4, although the process leading to the "TF category" in that figure seems to be completely missing from the Jolma manuscript and only described here – perhaps the figure S4 is in the wrong manuscript?

As per suggestion of the reviewer, we have removed **Figure S4** from Jolma et al, and included it as **Supplementary Fig. 8** in the current revised manuscript, together with associated changes to both texts. (This figure provides an overview of the DNA binding categories, conservation (TOPs, CTOPs) as well as their age.)

2. The point of figure 3C is not clear. The promoter sites have, by definition, more that 37% of their binding sites in promoters. The point appears to be that the GHT-SELEX peaks and the ChIP-seq peaks are highly concordant. Similarly, figure 3E shows the effects of the same definition of Dark TFs – more than 50% of the binding site are defined to be in regions of empty or heterochromatin. Rather than presenting that a second data type designed to measure the same phenomenon sees the same definitional thresholds, more effective presentation would be a global comparison of correlation of the ChIP-seq and GHT-SELEX data (which may be the point of the Vorontsov, et al, companion paper currently on biorxiv).

This comment prompted us to re-examine the presentation order of material in the manuscript, to make it easier to understand the data and appreciate the results. **Figure 3c**, **d**, and **e** are now **Fig. 4a**, **b**, and **c**.

The Jolma et al. paper entitled "GHT-SELEX demonstrates unexpectedly high intrinsic sequence specificity and complex DNA binding of many human transcription factors" contains these global comparisons (note that there are currently two Jolma et al. papers in review). A key point is that, while the overlap is often very significant, it is not a

measurement of the same phenomenon. This is because binding sites measured by ChIP-seq are influenced by both chromatin and cofactors. It is expected, and observed, that ChIP-seq data will contain many peaks that the same TF does not bind in GHT-SELEX (presumably due to indirect recruitment), and the GHT-SELEX will contain many peaks that are not bound in ChIP-seq (presumably due to inhibition by chromatin). The fact that they so often overlap is, in our view, a major finding, and indeed is a central point of the "GHT-SELEX" paper.

Moreover, the degree of overlap can be increased or decreased by choosing different peak-calling thresholds for either data type. In analyses later in the paper, and in Jolma et al. "GHT-SELEX", we optimize the peak numbers to obtain TOP sites (Triple Overlap between ChIP-seq, GHT-SELEX, and motif matches).

In **Fig. 4a**, **b**, and **c** (old **Figure 3c**, **d**, and **e**), however, it would be unfair to optimize the peak thresholds, given that we want to show that the two methods *independently* identify the same types of genomic regions. If we optimized the thresholds, then one data type would influence the results of the other, and we would be forcing the peak sets to overlap more than they do if they are considered independently. Thus, for **Fig. 4a**, **b**, and **c**, we used "universal thresholds" that can be applied separately and uniformly to each data type.

In section "**Selection of universal ChIP-seq and GHT-SELEX thresholds**" of the **Methods**, we now explain in greater detail what these universal thresholds are, and how they are derived. Briefly, the ChIP-seq threshold is $P<10^{-10}$ (MACS 2.0), while the GHT-SELEX threshold is defined as a Kneedle[1] specificity value of 30, with sorted MAGIX scores[2].

3. Analysis of Dark TFs is incomplete. For example, the transcription factor ZNF698 is reported in this manuscript to have a "much smaller number of CTOP sites, and is enriched for binding L1M5 elements" (line 372) later quantified to "~50 conserved sites enriched for L1M5" (line 527) out of a total of 558 ZNF698 binding sites (figure 4A). In Jolma, we learn that ZNF698 is an example of a strongly conserved binding site in an L1ME1 transposon just over 100bp from a predicted enhancer with a CTCF binding site. The information presented make it challenging to get a full picture of the relevant biology here. The Jolma example of the ZNF698 binding must be an example of a clustered CTOP that is a major focus of the Jolma manuscript, but ignored here where ZNF698 binding sites are presented as not highly conserved. Obviously both of these things can be true, but this presentation is scattered and incomplete.

The reviewer is correct that different aspects of the ZNF689 genomic binding locations are discussed in the two manuscripts, highlighting different points. The reviewer is also correct that both things can be true. The two papers concern different aspects of ZNF689, hence different aspects are shown, that are not mutually incompatible. This would be true of virtually any pair of non-redundant papers on the same protein.

As an aside, and to avoid confusion: in response to reviewer comments of the Jolma et al. "GHT-SELEX" manuscript, we made changes to the GHT-SELEX peak-calling procedure, which jittered virtually all of the numbers, without significant changes to the conclusions. The previous numbers of CTOPs/TOPs for ZNF689 (50/558) have now changed to 35/353. The text in the current manuscript has been changed accordingly (Lines 473 and 524).

4. The discussion of conserved triple occupancy sites (CTOPs) is also confused between this manuscript and Jolma making it hard to follow and ultimately unsuccessfully presented because key results necessary for this manuscript to make sense are omitted. Specifically, in this manuscript, we are told that CTOPs exist for both promoter TFs and Dark TFs, but the number and fraction of CTOPs in promoters is much higher (line 357) along with figure 4 showing promoter TFs. We are told that CTOPs adjacent to other CTOPs are explored in Jolma (line 363). In Jolma, we are told that there are 121,785 CTOPs in the Codebook and control sets and that the "strong support for the functional importance of Codebook TF binding in the genome" are summarized in greater detail in this manuscript. Examples of critical information omitted from this manuscript include Jolma providing results about what fraction of CTOPs are variant free, although we have no information in this manuscript about the fractions of Dark TF CTOPs that are variant free, which would give information about function of these binding sites (which according to the note in Jolma quoted above this manuscript is supposed to provide). We also have apparently no results presented here about how often CTOPs for Dark TFs cluster even though this clustering is considered in Jolma to be "indicating a likely biochemical function for these elements". The clustering of CTOPs and the relation of these to function are important enough in Jolma to take up nearly one page of text and most of one figure.

The two papers explore different aspects of the CTOPs because the papers have different themes. They provide complimentary perspectives that are not contradictory.

We appreciate the attention of the reviewer to Jolma et al., but we will focus here on aspects that are specifically relevant to the current manuscript (Razavi et al.).

Regarding the Dark TF CTOP variation and clustering:

- First of all, in our analyses, Dark TFs do not have many CTOPs. The 23 out of 34 Dark TF for which we have GHT-SELEX data have total of 2,546 CTOPs, many of which belong to ZBTB40 (n= 846) (**Supplementary Fig. 8** in the revised paper). In contrast, almost all the Promoter TFs (28/30) have CTOPs, and they are much more numerous. Here, they are presented mainly for contrast to the Dark TFs, since it is much less surprising that promoter-binding TFs would have many conserved binding sites in promoters: TFs are perhaps best known for binding to conserved sites in promoters.

- The current paper (Razavi et al.) had no analysis of variants, and the Jolma et al. paper refers to Razavi in the context of CTOPs, not variants. Nevertheless, to reassure the referee, 2,407/2,546 (94.5%) CTOPs of Dark TFs seem to be variant free based on our analysis. This observation does not appear to be particularly illuminating, and thus is not mentioned or discussed in Razavi et al.

- CTOP clusters are rare among Dark TFs, presumably because the Dark TF binding sites are mostly isolated, as **Fig. 2b** indicates. **Supplementary Fig. 2b** provides a more explicit enumeration of uniqueness, showing that Dark TF binding sites are much less likely to overlap with the binding sites of any other TFs (or an adjacent binding site for the same TF), relative to Promoter TFs, Enhancer TFs, and Other TFs. Thus, there is little to discuss regarding CTOP clusters for Dark TFs. Their absence does not constitute lack of function of the Dark TF binding sites, and Jolma et al. does not make such a claim. When CTOP clusters do exist, it is an indicator of potential joint function. But when they are absent, no conclusion can be drawn except that the binding site is conserved.

In summary, read in concert with the Jolma, et al manuscript and the Vorontsov, et al manuscript on bioxriv, the distinct feeling this reviewer gets is a reminder of the ENCODE pilot project publication effort in 2006 and 2007 where the consortium members divided the work into five publications that were overlapping, incomplete and fundamentally not particularly interesting on their own. The Nature editors wisely forced ENCODE to combine the results into a single paper (on which all five author lists remain) that become a seminal description of integrated genome biology. I believe a similar approach is appropriate here.

The proposed single manuscript would be very different from any of the standard journal formats and has not been offered to us as an option. We disfavour such an arrangement because it diminishes the credit that the first authors (who are mostly trainees) receive for their work. There is also the issue that few people read such long papers in their entirety – they can also be hard to follow. Far too many researchers in human genetics and molecular biology have not read the 55-page Lander et al. paper in its entirety.

Mandating such a publishing arrangement would ultimately be a decision for the editors, but it is not our preference and we would argue against it.

Reviewer #3 (Remarks to the Author):

Razavi et al. aim to better identify the binding motifs and sites of uncharacterized transcription factors (TFs) as part of a larger initiative called the “Codebook”. They

utilize various methodologies to measure TF occupancy in the genome. This represents a significant amount of work employing experimentally-sound methodologies, such as ChIP-seq, along with rigorous analytical processing. Multiple experiments were conducted with replicates across several cell lines by different researchers, focusing on both known and unknown TFs. They utilized the flp-in HEK293T cell line for genome-integrated, doxycycline (DOX)-induced overexpression of all TFs, confirmed by a GFP tag and immunoprecipitation.

The authors were very stringent in selecting binding sites for further analysis. They began with curation based on reliable motifs identified across multiple techniques used in previous studies. Next, they focused on TFs that exhibited consistently overlapping peaks between ChIP-seq replicates from this study to classify them according to their genome binding locations. Further filtering of the ChIP peaks involved overlapping them with the in vitro technique GHT-SELEX (conducted for another study) and assessing whether there is a motif match at the peak location. The inclusion of GHT-SELEX enhanced their confidence in identifying motifs bound by these uncharacterized TFs, which is a major goal for the authors. In their discussion, the authors first address the conservation of TF-binding sites and their evolutionary age while comparing "promoter" and "dark" TFs. Ultimately, they leverage their data alongside existing literature to provide insights into the function of TF binding in the dark genome, beyond merely silencing transposable elements.

Overall, this manuscript is well written, and the data support their conclusions. However, the study lacks functional validations of some of their interpretations, such as the importance of SOX2 binding to the dark genome in relation to its pioneering or reprogramming ability. Moreover, the authors highlight that their focus is limited to the identification of TF binding sites and not how this binding is linked to TF function. Nevertheless, I believe the extensive data released in this study will be highly beneficial for the field investigating TF binding patterns.

We appreciate the reviewer's positive comments, insight and helpful suggestions.

In response to how binding is linked to function, the manuscript now includes ATAC-seq and H3K9me3 ChIP-seq experiments for SOX2 (which we see as a control, although it is also true that this is valuable new data), as well as two other less-characterized Dark TFs (TPRX1 and ZNF836), demonstrating significant changes of chromatin state at the binding sites of these TFs in the "dark matter" genome upon ectopic over-expression (lines 479-500 and **Fig. 6** in the revised manuscript).

Minor Corrections:
- At times, I found it confusing to identify which TFs or peaks the authors were analyzing for each figure. A supplementary figure that explains the progressive filtering of proteins and peaks, indicating which are used in each main figure of the paper, would improve clarity.

This comment prompted us to re-examine the presentation order of material in the manuscript, to make it easier to understand the data and appreciate the results.

We have also added **Supplementary Fig. 3(a-c)** in the revised manuscript, which provides an overview of analysis workflow and definition of various classes of peaks (TOPs and CTOPs).

- In bubble/scatter plots (e.g., Fig. 2A or Fig. 3A-E) and lists of TFs (e.g., Fig. 3F and Fig. 5A), it would be informative for the reader to distinguish between control and Codebook TFs.

As per request of the reviewer, this information has been included in the revised version of all the figures. Note that some figures and panels have been rearranged.

- In Fig. 3A and B, I suggest changing the y-axis label to "TOPs" instead of "specific binding," as this is how they are referred to in the text.

This issue has been addressed in the revised version of **Figure 3**. Note that some figures and panels have been rearranged; these panels are now found in **Fig. 4**.

- Since the authors focus significantly on the age and evolution of TF-binding sites, should this be mentioned earlier in the abstract and introduction to attract the attention of potential readers?

Some of the age and evolution aspects of the TFs binding sites are now addressed in the second paragraph of the revised introduction (line 113-118), within the context of TEs "rewiring" gene regulatory sites:

"It is well-established that TEs can rewire regulatory circuitry by introducing transcription factor binding sites, thus spawning novel cis-regulatory elements[3-5]. In some cases, these elements derive from the promoter of the TE (e.g., endogenous retrovirus long terminal repeats (ERV LTRs))[6,7]. Other cases may represent inadvertent matches to a TF motif within a TE[8]; over evolutionary time, regulatory sites tend to turn over rapidly, arising seemingly at random, and TEs/EREs are a known contributor to this process[9]. …"

We note that the manuscript overall is now more focused on the binding of TFs to TEs; the previous **Figure 6**, and much of the associated text, is now **Supplementary Fig. 9** and **Supplementary Document 4**.

- Regarding lines 397-398, Figure 5A: If these are indeed the TOP sites, then the strong correlation between ChIP and GHT-SELEX is expected, as they have filtered each other already.

We are grateful to the referee for pointing out this unintentional circularity. In the revised version of the same figure, we have applied the analysis to the universal peaks before overlapping. (The previous **Figure 5A** is now **Fig. 3b**).

Reviewer #4 (Remarks to the Author):

In this manuscript, the authors report novel analyses on the genomic binding preferences and sequence specificity of many uncharacterised transcription factors (TFs). Their findings are based on substantial ChIP-sequencing datasets obtained in HEK293 cells upon inducible expression of a set comprising 315 uncharacterised TFs and 58 control TFs.

Following quality control of these data, the authors report genomic binding preferences across promoter, enhancer and heterochromatin genomic regions (defined via canonical histone mark enrichment). This allows them to define subsets of uncharacterised transcription factors preferentially binding promoters, enhancers or “dark” heterochromatin in HEK293 cells; however the rest of the study largely focuses on TFs binding preferentially to promoters or heterochromatin (“dark TFs”). The manuscript next focuses on investigating the relationship between in vivo and in vitro binding preferences across the two classes, examining conservation patterns of sequence motifs within in vivo binding sites, and characterising the association of genomic binding and sequence specificity with repetitive elements (in particular for “dark” TFs) and the evolutionary age of binding sequences and transcription factor proteins. Lastly, the authors propose candidate functions on transcriptional repression across “dark” TFs with genomic binding to heterochromatin regions, based on available literature evidence and genomic, proteomic and epigenomic data.

Overall, this is a very interesting study reporting a novel resource to explore the biochemical signatures and potential roles of a large number of previously uncharacterised proteins with predicted TF function. The authors' results are likely to be significance to a wide readership, given the novelty of the interrogated proteins and the scope of the presented analyses. However, in its present form we have some concerns on the clarity of presentation in some areas, and the contextualisation of observations and interpretations to the author’s experimental system and supporting data.

Major concerns:

1) Although some details are included in the manuscript, the authors should be clearer on how ectopic expression of TFs in their HEK293 experiments compares with endogenous TF binding in previous ChIP-seq datasets. To the author’s credit, their design includes 58 control transcription factors. Incorporating a more detailed comparison of their HEK293 data for these proteins versus available endogenous datasets would be helpful to provide a benchmark for interpretation of results on their

uncharacterised putative TFs. For instance, from the data presented it is not clear whether genomic binding of control TFs (e.g. CTCF or YY1) significantly differs from endogenous datasets (whether in the same regulatory context i.e. HEK293 cells or in different cell lines or tissues). There is substantial evidence in the literature for promiscuous TF binding in ChIP-seq data upon ectopic TF expression, and the authors should address this potential caveat with their control datasets.

The manuscript now includes the proposed comparisons in **Supplementary Fig. 1**. Briefly, we compared our peak sets for four control TFs (CTCF, YY1, MAX, GABPA) with at least two external (i.e. previously published by another group) ChIP-seq data sets performed against the endogenously-expressed TF in HEK293, K562, or HepG2. The Jaccard similarity score for peak overlap between our data set and the other data sets (i.e. Codebook vs. GTRD) is comparable and often exceeds the Jaccard score between different endogenous experiments. The new **Supplementary Fig. 1** shows the similarity score at a range of peak numbers, illustrating that the trend is independent of thresholds.

More clarity on the above may also help interpret some of the results in the manuscript. For instance, the observed low number of novel TFs with preferential binding to enhancer elements may be partly explained by ectopic expression of transcription factors in a single cellular and chromatin context (which could be compatible with the authors observation on the 'GeneHancer' set, lines 209-213). For control TFs, are genomic binding preferences across promoters and enhancers similar between the author's ectopic data in HEK293 and various endogenous contexts across previous datasets?

As per reviewer's suggestion, we compared the fraction of endogenous peaks (ChIP-seq or ChIP-exo) for control TFs in various cell lines with our own data (more specifically for Promoter and Enhancer TFs). Reassuringly, the newly added **Supplementary Fig. 4c-e** indicates that the binding patters are consistent between the datasets across the control TFs. Intriguingly, JUNB and SRY (which we classify as Enhancer TFs, consistent with the literature) bind the GeneHancer loci more specifically in the Codebook set than they do in the external endogenous context.

2) On a similar note, the author's observations in Figure 2 regarding much lower correlations in binding positions across "dark" TFs compared to promoter TFs should be supported with more detailed analyses. As the author's rightly point out, heterochromatin can be systematically depleted from ChIP-seq experiments due to sonication biases, which could partly explain the author's observations. This caveat could also suggest ChIP-seq data for heterochromatin-binding TFs may have systematic experimental biases, such as lower signal to noise or a lower number of reproducible peaks. To ensure the data can be interpreted accurately, the authors should incorporate analyses explicitly comparing metrics such as number of reproducible peaks, genome coverage across all peaks and fractions of reads in peaks

across the three TF classes they define. For instance, does data for “dark” TFs typically yield lower numbers or reproducible peaks, or peaks that cover a smaller fraction of the genome? Some of these data is available in the supplemental tables, but is not explicitly annotated or compared (for instance, a TF class column for promoter, enhancer, dark or other should be added to Table S3).

ChIP-seq is known to be biased towards open chromatin, so it is almost certain that there is a bias against detection of Dark TF binding sites. Also ChIP-seq detects indirect binding. Thus, to compare the number of peaks, we considered the number of TOPs (i.e. sites with ChIP-seq peaks, in vitro binding, and a motif match), instead of the number of peaks that are reproducible in ChIP-seq. **Fig. 4f** shows that the number of TOPs is slightly less for Dark TFs than it is for Promoter TFs, although the distributions overlap closely.

We do not have a complete set of replicates for some TFs, but the median optimized Jaccard for replicates of Dark TFs is 0.59, and for Promoter TFs, 0.55. Even at the standard threshold ($<10^{-10}$), the peak overlap between replicates of the same Dark TF is much higher than that between mismatched Dark TFs (**Supplementary Fig. 2a**). Manual inspection suggests that some of the non-overlapping peaks between replicates are explained by thresholding; although many of the Dark TF peaks are clearly visible in both replicates, but only in one replicate (and not the other) they are called as peaks. Several examples are shown in the new **Supplementary Fig. 7c**. (Note that the peak sets for replicates were merged for the main data analyses throughout the paper).

As per the reviewer’s suggestion, we have now included analysis of the Fraction of Reads in Peaks (FRiP) scores. The newly added **Supplementary Fig. 7a** shows the FRiP for the Promoter TFs and Dark TFs are similar. In both Promoter and Dark TFs and at $P<10^{-10}$ peak calling cut off, more than 75% of reads are not within peaks. This is a consistent trend: at more liberal cut off threshold of $P<10^{-5}$, more than 60% of reads are outside of peak regions in both cases. Altogether, there appears to be no systematic difference in this measure between Promoter TFs and Dark TFs.

Finally, we have added at TF class column to **Supplementary Tables 3**, together with these new metrics (optimized Jaccard for replicates and FRiP). We have also merged **Table S3** and **Table S4** (which previously contained the class labels), given that they are both tables of TF properties.

3) The authors should consider a more careful use of the term “functional” across the manuscript, especially in its current context as no experimental validations are presented. As the authors mention in the discussion, the relationship between biological function and evolutionary turnover is debated in the context of gene regulation. In the later figures of the manuscript, the authors are also using the term “functional” repeatedly to describe supporting literature evidence (which can be said to be limited, as these are largely uncharacterised TFs) and proteomic, genomic and epigenomic annotation/data. A more cautious presentation of these analyses and their conclusions

would help contextualise these results as largely based on biochemical readouts (rather than experimental validation experiments). Indeed, incorporating a few targeted validations in Figure 7 (for instance by measuring changes in heterochromatin following expression of candidate "dark" TFs) would have strengthened the authors' results.

We have now revised the section of the manuscript that describes the "functional" impacts of Dark TFs. In the new version, we have included newly obtained ATAC-seq and H3K9me3 ChIP-seq data for cell lines in which SOX2, TPRX1, and ZNF836 are induced. The current **Fig. 6** indicates significant changes of chromatin state at the binding sites of the selected TFs upon their ectopic over-expression.

Minor comments:

- The methods are presented succinctly and, on occasion, in a convoluted manner. For instance, the section on ChIP-seq peak calling and the author's use of input controls is difficult to follow as presented. Have the author's used input controls matched to experiments for each TF (i.e. in a way that inputs correct for experimental batches and variables such as cells passage number, days under selection, etc)? Or is their approach rather based on use of a more limited set of input experiments and a "consensus" set of background control regions, in a manner similar to greylisting? From the current description in lines 640-661 it is not clear which of the two approaches above is conceptually closer to the author's method (although details in Table S2 seems to suggest inputs are not matched for each TF's cell line). The authors should consider carefully revising the methods for clarity and detail, and expand if needed (there do not seem to be supplementary methods elsewhere in the manuscript).

As per reviewer's suggestion, we have rewritten the relevant Methods section for clarity (lines 673-675 and 705-730):

"For each batch of ChIP-seq experiments, we also collected "input DNA" (i.e., non-IP sonicated chromatin), chosen at random for ~10% of all the experiments (see **Supplementary Table 2**)."

"**TF ChIP-seq background generation and peak calling**. We generated sample-specific background models using a previously established approach[10], with minor modifications. For each ChIP-seq pull-down experiment, our goal was to construct a background control that closely matched its nonspecific signal while maintaining high coverage by pooling reads from multiple "input" experiments in proportions that best reproduced the background characteristics of the given pull-down.

First, we identified genomic regions showing high background signal in at least one input DNA experiment. To do so, we performed peak calling on each input sample using MACS2 (options: p-value < 0.001, and *--nomodel*)[11-13] and collected the resulting peaks

as “background hotspots”. We merged hotspots whose summits were within 50 bp across experiments to produce a unified set of background regions.

Next, we quantified read counts overlapping each hotspot. For each input DNA experiment, we counted the number of reads overlapping every region in the unified hotspot set, creating a read count matrix with rows being the hotspots and columns being input experiments. For each pull-down experiment, we also calculated read counts over the same hotspot regions; these served as the response variable in the following modeling step. We modeled the pull-down read counts using a non-negative Poisson regression, with the input DNA read count matrix as predictors. This regression estimated a set of non-negative coefficients (one coefficient per input experiment) representing the weighted combination of controls that best reconstructs the background signal observed in the pull-down.

Finally, we used these coefficients to generate a synthetic background dataset. Specifically, we sampled reads from each input BAM file in proportion to its corresponding regression weight and pooled them to form a single, pulldown-specific control file. This customized control was then used as the background input for peak calling on the pull-down sample with MACS2[11-13].”

- It is not clear from the manuscript how the candidate 315 uncharacterised TFs have been selected with regards to their tissue-specific gene expression. Are properties such as tissue-specific expression scores different across the classes of TFs the authors define (e.g. in Figure 2A)? Similar to comments above, the authors may want to incorporate supporting analyses in this area, for instance using human GTEX data.

Tissue-specific gene expression was not considered in the selection of TFs, and not used in any of our analyses, except to choose the three TFs for functional analysis. For the functional analyses, we reasoned that TFs with low expression in HEK293 would be most likely to show effects upon induction.

We now mention in the Discussion (lines 632-635) that analysis based on GTEx data and tissue expression is an excellent starting point for future functional analysis, but not something we are prepared to report on in this manuscript.

- Being clearer across the manuscript about the numbers of TFs represented in the various analyses would add clarity to the authors’ data presentation. As far as we can tell, the authors are presenting 217 TFs in Figure 2 and 137 TFs in Figures 3 and 4. This can be gleaned from the text but could be clearer in the figures themselves and their legends. In later figures this information could be made for explicit (e.g it seems less than 317 TFs are selected for Figure 5, but it is not clear what specific numbers are included in this analyses).

This comment prompted us to re-examine the presentation order of material in the manuscript, to make it easier to understand the data and appreciate the results. We anticipate that the revised manuscript is more clear.

We have also now included a new figure (**Supplementary Fig. 3**) that provides an overview of all the TFs used in various analyses, as well as classifications of peaks that were used/defined in different figures.

- The data presentation in some of the figure panels could be made clearer. Some examples:
- The difference in Figure 2B are very difficult to see with the current shading and magnification. The authors could consider using a white background for this plot, and/or including separate correlation plots for Promoter&Enhancer vs Dark TFs
- The right hand-side panel in Figure 3F does not clearly relate to the presentation of this figure in the associated results section, in which CTOPs have not been yet introduced as a concept. The authors could consider removing this panel for clarity (also given the following figure focuses on CTOPs).
- The panels in Figure 5A are very dense and difficult to follow. For clarity, the authors may consider tweaking the data presentation here.

We attempted a white background in **Fig. 2b**, and to our eyes, its interpretability is unimproved. We are also making an effort to have very different colormaps for different data types that are all represented as heatmaps. A key point of **Fig. 2b** is to contrast the TF categories, which is not possible if they are shown in different panels. The panels within Figure 3F (**Fig. 4d** in the current manuscript) are interrelated. We did minimize two of the legends in Figure 5A (presented as **Fig. 3b** in the current manuscript).

- The authors may need to clarify any limitations in their ability to map TF binding to repetitive elements in the reported ChIP-seq data (especially for young repeat families with many identical or near-identical copies across the human genome). This limitation may not apply to the specific examples highlighted in figures, but even then would be useful to comment on for clarity.

The read mapping stringency employed (MAPQ score >30) is quite high, and multi-mapping reads were rejected. This ensures that peak locations are unambiguous, but it is possible that the elimination resulted in some underestimation of binding to the youngest families, in which members are nearly identical to each other (e.g. L1HS). This would only lead to false-negatives, however. We have added this caveat to the Methods (lines 698-704):

“This stringent threshold on mapping quality ensures that reads mapped to multiple locations are removed; hence, the peak locations (see below) are unambiguous, and our analysis of repeat elements is not skewed. This elimination may, however, result in missing potential peaks in young repeat families with identical or near-identical copies

(e.g., L1HS). This could lead to an underestimation of the proportion of binding sites in the dark genome, as repeat elements are often found in those regions, but it would only result in false negatives.”

- The analyses presented in Figures 6B and 6C suggest the combined information on binding sites age (6B) and TF sequence age (6C) may discriminate between promoter and dark TFs. The authors may want to explore this possibility and specify whether this is the case.

This material has been moved to **Supplementary Fig. 9** and **Supplementary Document 4**.

Nonetheless, we asked whether the ages of TFs and binding sites (TOPs) could predict the TF class labels, using random forests. The scatterplot below shows that the TF classes are intermingled but by eye it does seem as if it would be possible. The classification ability on held-out data was near random, however (AUROC = 0.98 on the training data, but only 0.58 on the held-out data, which is near random, with a five-fold split). It is also important to note that the TOP age (vertical axis) is capped at 100 Mya, because the age of TOPs can currently be estimated only within eutherian mammals. This approach may be more effective with more data and more advanced approaches. Since it is not a critical point of the study. We did not continue with this analysis, and it is not included in the paper.

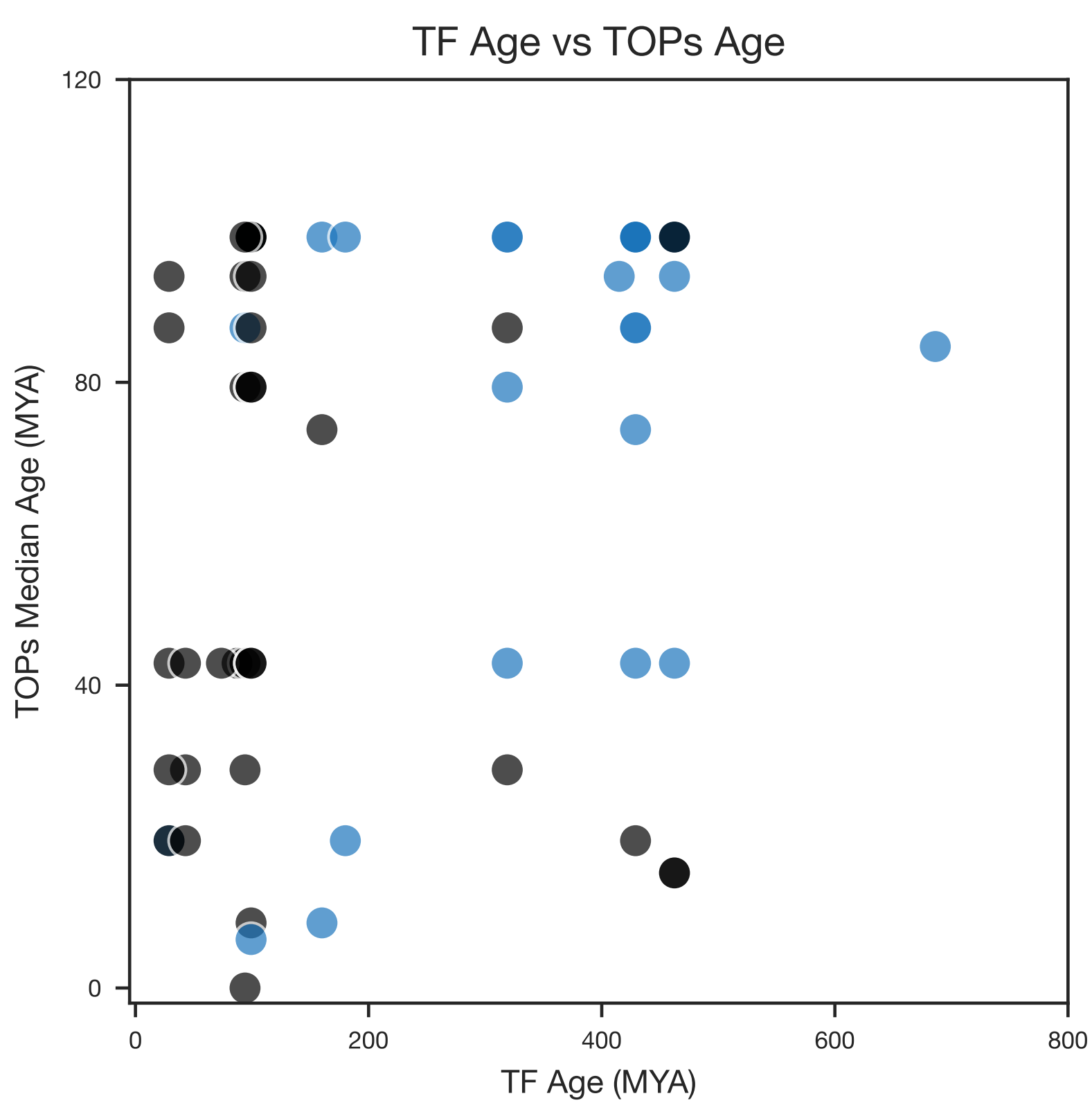

- Referencing in the discussion section is rather sparse and should be revised.

The Discussion has been revised, and several references added.

Reviewer #6 (Remarks to the Author):

I co-reviewed this manuscript with one of the reviewers who provided the listed reports. This is part of the Nature Communications initiative to facilitate training in peer review and to provide appropriate recognition for Early Career Researchers who co-review manuscripts.

1. Satopaa, V., Albrecht, J., Irwin, D. & Raghavan, B. Finding a" kneedle" in a haystack: Detecting knee points in system behavior. in *2011 31st international conference on distributed computing systems workshops* 166–171 (IEEE, 2011).
2. Jolma, A. *et al*. GHT-SELEX demonstrates unexpectedly high intrinsic sequence specificity and complex DNA binding of many human transcription factors. *bioRxiv*, 2024.11.11.618478 (2024).
3. Cosby, R.L. *et al*. Recurrent evolution of vertebrate transcription factors by transposase capture. *Science* **371**(2021).
4. Schmidt, D. *et al*. Waves of retrotransposon expansion remodel genome organization and CTCF binding in multiple mammalian lineages. *Cell* **148**, 335–48 (2012).
5. Chuong, E.B., Elde, N.C. & Feschotte, C. Regulatory evolution of innate immunity through co-option of endogenous retroviruses. *Science* **351**, 1083–7 (2016).
6. Cohen, C.J., Lock, W.M. & Mager, D.L. Endogenous retroviral LTRs as promoters for human genes: a critical assessment. *Gene* **448**, 105–14 (2009).
7. Ernst, J. *et al*. Genome-scale high-resolution mapping of activating and repressive nucleotides in regulatory regions. *Nat Biotechnol* **34**, 1180–1190 (2016).
8. Kunarso, G. *et al*. Transposable elements have rewired the core regulatory network of human embryonic stem cells. *Nat Genet* **42**, 631–4 (2010).
9. Villar, D. *et al*. Enhancer evolution across 20 mammalian species. *Cell* **160**, 554–66 (2015).
10. Schmitges, F.W. *et al*. Multiparameter functional diversity of human C2H2 zinc finger proteins. *Genome Res* **26**, 1742–1752 (2016).
11. Feng, J., Liu, T., Qin, B., Zhang, Y. & Liu, X.S. Identifying ChIP-seq enrichment using MACS. *Nat Protoc* **7**, 1728–40 (2012).
12. Liu, T. Use model-based Analysis of ChIP-Seq (MACS) to analyze short reads generated by sequencing protein-DNA interactions in embryonic stem cells. *Methods Mol Biol* **1150**, 81–95 (2014).
13. Zhang, Y. *et al*. Model-based analysis of ChIP-Seq (MACS). *Genome Biol* **9**, R137 (2008).

REVIEWER COMMENTS

Reviewer #1 (Remarks to the Author):

The authors have addressed all of my concerns and questions, and added new data to substantiate their interpretations and conclusions. The manuscript has significantly improved and I am pleased to see the current form of manuscript, including the title and abstract, which are more appropriately describing the TFs that are part of their identification and profiling. I have seen the other reviewers raised similar and novel comments, that I believe were handled well by the authors as well.

Reviewer #2 (Remarks to the Author):

The authors have revised their manuscript in response to my comments and those of the other reviewers. Although the manuscript is improved especially in way that the results are self-contained and not excessively reliant on cross-reference with other manuscripts, there are still a number of concerns that limit the impact of this work.

Major comments:

1. The inclusion of unbiased clustering of the TF binding features in supplementary figure 6 is appreciated. Although partly reassuring that the overlap between the manual classification and the clustered one does not include any classified dark TF clustered with classified promoter TFs (or vice versa), the author's assertion in the rebuttal that their manual classification "produced more stringent results" and "removes TFs in which the classifications are less clear" is without any presented evidence in the rebuttal or the manuscript. In fact, based on the clustering in many cases they failed to manually classify TFs that cluster extremely closely with TFs that they do manually classify. This effect is most striking for dark TFs, but occurs in all categories. Additionally, the authors describe the clustering results as "very similar" to their manual classification, when in fact 32% (69/127) factors change classification.

For example, only ~60% of the TF that cluster into the dark category are classified as dark TFs for the subsequent analysis. Considering the general focus in the manuscript on global characteristics of dark transcription factors, this feels like either a bias or a missed opportunity.

Specifically, the authors make the point that the set of dark TFs has a number of

fundamentally different characteristics compared to the set of promoter TFs and this is true whether the manual or clustered sets are chosen. However, nearly all of their claims about the characteristics of the dark TFs seem to be considerably less trustworthy given the large number of TFs (including many than apparently cluster very closely) that they have manually excluded from the analysis.

It is, of course, a challenge to create a sensible partition of the transcription factors to anchor the various analysis in this paper. However, the process used here dramatically limits general statements that can be made. If the authors continue with their manual classification, they must provide both a more complete justification, potentially with a workflow, to describe the "meaningful large-scale trends" that they used to create the set. Their current approach appears to be ad hoc either because it is or because they have made classification decisions that are not transparently presented.

It is true that our approach is *ad hoc*, in the sense that it is a simple solution for an immediate purpose. We believe it is also presented transparently:

"We sought to gain a better understanding of the properties and characteristics of TFs (and their binding sites) that represent the major patterns shown in **Fig. 2**. As a starting point, we defined four mutually exclusive groups (see **Supplementary Table 3** for TF labels) based on the dominant annotation types that overlap with their genomic binding sites (i.e., ChIP-seq peaks). One group we named "Promoter TFs" (56 proteins); for these proteins, more than 45% of peaks exclusively overlap with promoters (this threshold captures the most prominent features in **Fig. 2a**). Another group was designated "Enhancer TFs" (12 proteins); for these proteins, >37% of peaks overlap with enhancers (this threshold corresponds to the visual separation of data points on the vertical axis in **Fig. 2a**). A third group we named "Dark TFs", after the genomic "dark matter" (54 proteins); for these, most peaks lie within either the "empty", "constitutive heterochromatin", or "facultative heterochromatin" states (i.e. in HEK293 cells, they are outside of the states that represent promoters, enhancers, insulators, or gene bodies), and fewer than half of the peaks overlap with ATAC-seq peaks in unperturbed HEK293 cells. The remaining ~40% of TFs we labeled as "Other" (i.e., not Promoter, Enhancer, or Dark TFs); they include 30 control TFs and 65 Codebook proteins, which display diverse combinations of binding sites within the defined categories that do not reach our classification thresholds. The "Other TFs" thus present a rich landscape for additional exploration, but we did not attempt to further subclassify them here."

Clustering is fundamentally a form of exploratory data analysis, but to our knowledge, it is not intended for discriminative tasks, and should not be taken as a default gold standard for such tasks. The exploratory analyses shown in Figure 2, which do employ clustering, are what led us to develop a simpler categorization scheme.

We used careful observation and human judgement in order to generate simple classification rules, but strictly speaking, they are not manual classifications – they are numerical criteria, which are easily automated.

Obviously, slightly different conclusions might be reached depending on where one draws a line on a sliding scale. To us, the halfway mark seems a reasonable place to start, i.e. does something “mostly” do one thing or another. We adjusted the promoter and enhancer thresholds slightly to capture what seem to us to be clear trends in the data, but left it at a simple majority rule for the Dark TFs.

There are also specific reasons to avoid the use of clustering in this particular application. First, the similarity metric typically used in functional genomics (and that we used here) is Pearson correlation, which does not penalize for small effect sizes: [0,0,0,1,2] correlates perfectly with [0,0,0,50,100]. Thus, TFs that bind almost exclusively to heterochromatin can potentially cluster with those that mainly bind elsewhere but have some small tendency to bind heterochromatin. We believe it would be a mistake to group the two, if we want to study TFs that primarily bind within heterochromatin.

Second, the similarity obtained will depend on what features are incorporated. The features shown in our figures were of interest to us, and are common in the literature. If we add or omit features, however, or scale them differently, then the correlations and groupings can change.

It is also important to emphasize that the clustering analysis, which we added at the request of the reviewer in an earlier round of review, does indicate that we are not misclassifying TFs, or missing a major category. The referee is concerned that “32% (69/127) factors change classification” (referring to the threshold-based classifications, relative to the clustering), but these changes are all cases in which a TF that did not did not meet our thresholds (i.e., did not display a sufficiently uniform property to be labeled as “Promoter”, “Enhancer” or “Dark”) was moved into the “Other” category. Hierarchical clustering groups almost everything with something else - that is its nature. The goal in this case is to isolate the experiments with strong effects from those with the weaker and/or more mixed effects, and the thresholds are doing that.

For clarity, and to address reviewer concerns, we have rewritten the segment describing this new clustering analysis, removing the phrases that the referee quotes (that the classification by thresholding “produced more stringent results” and “removes TFs in which the classifications are less clear”, relative to the clustering analysis). The paragraph now reads as follows:

“The choice of features and thresholds to define the categories of TFs was to readily draw distinctions between TFs via interpretable binding behaviors. Standard clustering

analysis of the TFs using all the tracks presented on the top in **Fig. 2b** results in the same groups we defined above, but with “Other” TFs segregating with either the Promoter TF, Enhancer TF, or Dark TF groups (**Supplementary Fig. 6**), suggesting that no major category is omitted. Our thresholds do exclude some TFs that may associate significantly with specific regions but also bind many other locations; for example, the control TF YY1 bound 58% of all promoters in human, but it also had 40,764 additional binding sites (72% of all its binding sites) outside promoters. Similarly, some KZNFs were not classified as Dark TFs because they had many binding sites within open chromatin.”

2. The effort to better organize this paper and separate it from other papers of the Codebook consortium is appreciated. The revisions and the change in the order the results are presented have made significant progress here and the rebuttal provides more clarity about what the authors are trying to do in this manuscript and in the companion manuscripts. Specifically, the authors are clear that they are not trying to completely characterize individual transcription factors as aspects of the biology of a given factor may be split between their analysis manuscripts. Instead, they are trying to make general statements about their set of dark TFs and provide examples that illustrate this point. This is a valid approach likely to have the most impact.

Following this, once the authors commit to focus on a comparison between promoter and dark TFs (page 9 lines 315-316), they should strongly consider omitting the “other” transcription factors from the main text figures unless necessary for very specific and limited points. This is especially true for figure 3b where the inclusion of the “other” factors muddies the point of this panel making an already difficult figure harder to understand. Of course, one reason that almost 40% of the factors presented in figure 3b are classified as “other” (17/45) is that they are misclassified and actually cluster with the dark transcription factors, potentially suggesting again that their manual classification scheme seems to have considerable problems in reflecting transcription factor biology that the manuscript is attempting to described and is potentially inappropriate.

We thank the referee for suggesting ways we can make the paper easier to follow. A key point of Figure 3b, however, is that the correspondence between TFs binding to specific classes of transposable elements, and TFs that mainly bind within “empty” regions or heterochromatin (which we call “Dark TFs”), is far from perfect – to suggest otherwise would be both untrue, and to avoid the observation would be an oversimplification.

That said, in general, the “Other” TFs are much less likely to bind TEs and repeat elements, as shown in the graph below (right side). The “Other” class is clearly drawn from a separate distribution than the “Dark” class.

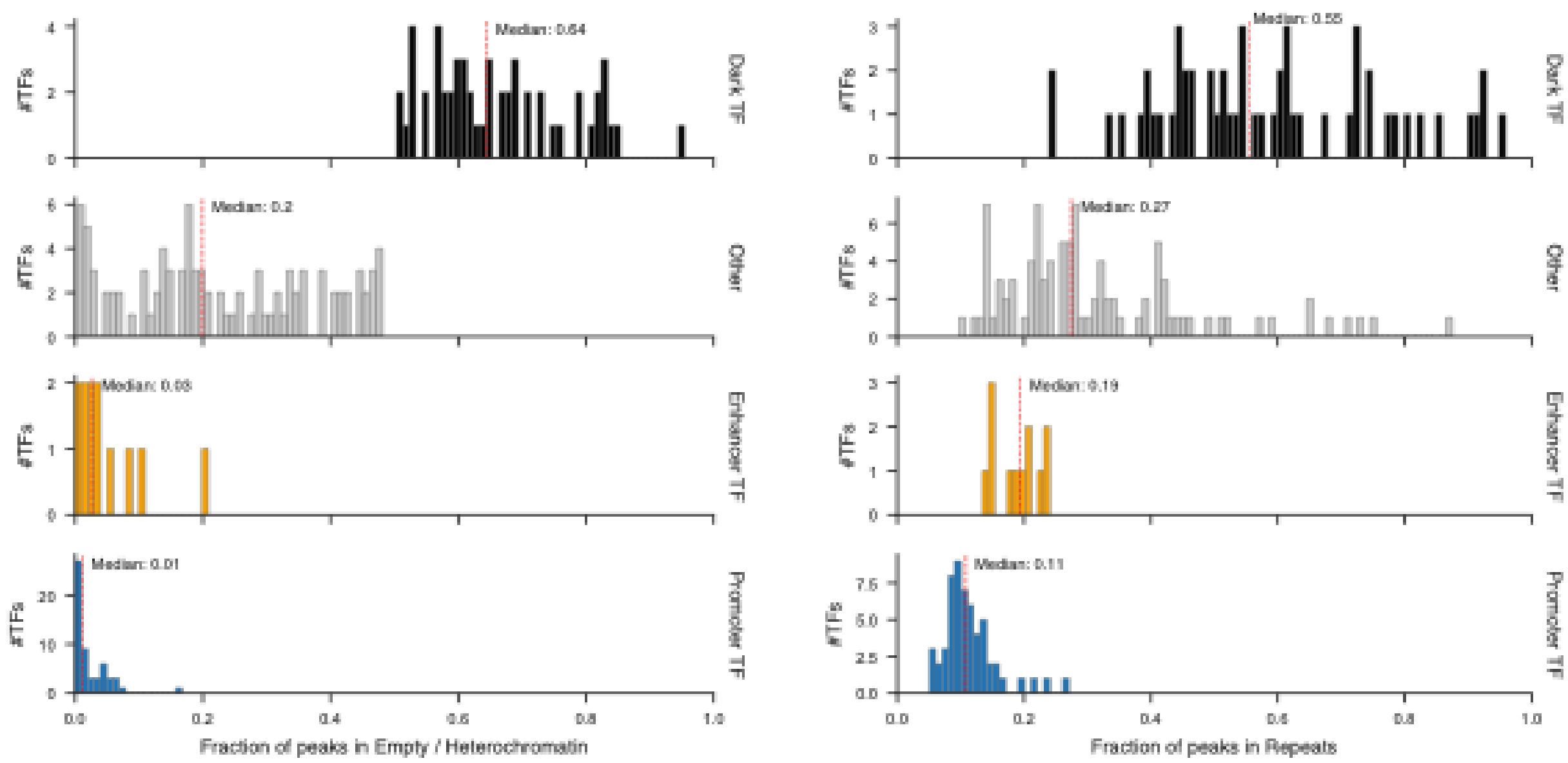

In sum, this manuscript describes a potentially very important dataset, but one that has seemingly been analyzed incoherently based on a manual classification that the author’s own results seem to show is poorly justified and potentially problematic. This flaw substantially mars the results.

Other comments:

1. In the first paragraph of the introduction, the authors state that “function of genomic dark matter in gene regulation is also supported by the existence of TFs that are capable of binding TE and ERE sequences” – this is not logically correct. The existence of DNA binding proteins that bind TE and ERE sequences is evidence that these DBPs are functional and that their function is to bind and/or silence TE and ELE elements. The binding itself is not evidence that these regions participate in gene regulation.

We have replaced the word “supported” by “suggested”.

2. The definition of a conserved TOP as having at least one base pair with an FDR corrected phyloP score greater than one is appropriate for setting a threshold on a set of TOPs to analyze for confirmation. However, the suggestion that “the corresponding TFs have conserved functions at promoters” or other binding sites (page 12, lines 449-455) is not supported by the data with these thresholds. The is a comment to soften the language of functional claims rather than challenging the CTOP analysis.

Our reading of the text is that it is already tentative in tone. The full sentence quoted is “This outcome suggests that many Promoter TF binding sites are functional, and that the corresponding TFs have conserved functions at promoters.”, i.e., the sentence as is contains noncommittal language (“suggests”).

We note that the full definition of CTOPs is more rigorous than one conserved base. The paragraph in question describes three statistical tests, and ends with “We considered a site to be conserved if any of the three criteria were met, and at least one nucleotide in the PWM hit had an FDR-corrected phyloP score $\geq$1.”. i.e., the threshold the referee states must be met, and in addition, at least one of the other criteria must be met at the same time.

Finally, we do not make any conclusive statements about individual TOPs or CTOPs, although some examples are shown. Mainly, the TOPs and CTOPs are used to examine average behaviors, e.g., comparing Dark TFs to Promoter TFs, and assessing for positional bias relative to TSS.

3. Jolma, et al is listed twice in the references (35 and 48)

Thank you, this has been corrected.

4. TOP sites are originally defined as “triple overlap” sites, but sometimes called “triple optimized overlap” sites. There are also occasionally references to “optimized thresholds”. In the supplementary material there are “triple peaks” which is clearly something different from TOPs. This terminology confusion may be a remnant of addressing my previous comments about the overlap and confusion between this manuscript and Jolma, et al. Regardless of the cause, the triple and TOP terminology should be thoroughly reviewed throughout the manuscript to ensure consistency. This is especially important because the characteristics of the TOP sites are a major part of this manuscript.

The reviewer is correct, and we have now reviewed all of the documents to ensure consistent usage.

5. Figure 7 has different annotations on the top (DBD category) and in the legend (TF family).

The reviewer is correct, they now both say “TF family”.

6. The figure style is figures 4e, 4f, 5b, 5c, S9b and S9c is misleading. For all of these figures, the x axis is simply an individual transcription factor ordered by the value on the y axis. There is no connection between the factors, so it is inappropriate to connect the

plotted values with lines. These data may be more appropriately plotted by box plots or in separate panels for promoters and dark TFs (i.e. each current panel becomes two panels). If the authors feel that there is explanatory value in using shapes to annotate controls and codebook TFs, the latter may be the best option. At a minimum, the plot should be remade without the connecting lines.

The adjacent points are connected in the sense that they are sorted, and they are all the same measurement on the same data type. The different series of points represent two different classes of TFs. The point of the graph is to compare two distributions, making them similar to cumulative distribution plots, which show differences in the shape of two sorted distributions of scores. Such graphs are typically drawn as a line, even when they are showing individual points that are sorted by score. It makes it easier to see the distribution. No logical connection between the points is implied.

It is true that we could show box plots, but those would not show all of the individual points, unless they are jittered and superimposed on the box plot.

In any case, the layout of the current plot is beneficial for display, in that it provides white space for labeling the individual TFs.

More generally, as mentioned indirectly in my comments and directly by one of the other reviewers, both the manuscript text and figures are often complicated, dense and difficult to parse. The revision has partly addressed this, but much of the manuscript remains difficult.

Many of the main text figures seem to have as their main message “we have a lot of data” and should be presented in more straightforward ways within the main text to highlight specific results and/or key differences between the promoter and dark TF sets while putting the complete figure into the supplementary material. Figure 3 is especially difficult to make sense of as are figure 2b, figure 4d and figure 7. Simply put, one main text figure early in the manuscript is sufficient to make the claim that there is a lot of data, other figures comparing specific differences between promoter and dark TFs (i.e. not many differences simultaneously as in the impossible to parse figure 3a). Finally figures with individual TF results where figure 5 and figure 6 are both already excellent in this regard.

We acknowledge that the TFs are behaving in a more complicated way than our brains would like them to – that is one of the key points of the study.

In addition, these are rich data, and the comparisons needed to address even the most obvious questions are multidimensional. We do not believe any of the analyses can be left out without compromising other parts of the paper.

We believe that the basic messages are accessible. To our reading, the Results present different analyses in a linear order, and the figures do correspond to individual questions.

7. Finally, it is true that many researchers have not read the nearly 25-year old Lander et al. paper in its entirety. It remains to be seen whether the work described in this manuscript is equivalent to sequencing the human genome.

We did not intend such a comparison to our work. Lander et al. is simply a very prominent example in which grouping everything into one big manuscript clearly had disadvantages for the community, not to mention the authors doing the work. The average researcher in human genetics has almost no knowledge about the repeat composition of the human genome, even though it comprises 20% of the human genome paper, and 50% of the human genome. If this happened with the human genome project, then surely a similar embedding of the Codebook data presented here would also suffer from inaccessibility.

Reviewer #3 (Remarks to the Author):

The authors have addressed all my concerns and I fully support the publication of the manuscript in Nature communications.

Reviewer #4 (Remarks to the Author):

The authors have performed a careful revision of the manuscript that addresses most of our comments (and those from the other reviewers). Their revision includes the addition of new data and analyses, and results in a much improved manuscript. It was particularly nice to see they now have incorporated some validation experiments and improved the narrative presentation of the study.

We have some minor comments regarding data presentation and interpretation that the authors are encouraged to consider:

- Some of the data presentation in the main figures could be improved to draw the attention of readers to key observations in the author's analyses, especially for some of the more complex plots. An example is Figure 5a (phyloP scores across TOP sites for two promoter and two dark TFs). It seems the key observation in these analyses is the signature of selective constraint on key positions in the PWM for these TFs, yet this is somewhat buried in a complex plot that also shows "background" signal in a large

window of surrounding sequence and unconserved sites. The authors should consider expanding the data around the motif logo for each TFs' conserved sites, which would improve readability of the plot and draw the reader's attention to this key observation. A similar concept could be applied to key TF/repeat pairs in Figure 3b.

We assume the referee intended that we should zoom in on the center part of figure 5a – we have done this; it seems reasonable – and to add add a few pullouts or zoom-ins on Figure 3b, which we have also done. We thank the referee for these recommendations.

- The data is Figure 6 could be made more compelling by addition of a graphical diagram outlining the experimental design for these experiments (as described in the results). We think this is really interesting new data, and its impact is somewhat diminished by the current presentation.

We have added a new panel, Figure 6a, that outlines the experimental design and data presentation.

- The discussion could be streamlined and referenced in a more rigorous manner, especially for the last two paragraphs. Some of the discussion about the relationship between evolutionary conservation and TF function reads somewhat oversimplified, including with regards to the relative age of TFs and their binding sites. It should also reference key work in this area.

We have reworked the last two paragraphs, adding ten new citations.

- Similarly, the discussion should mention some of the key limitations of the study. A surprising omission in this regard is the fact the author's data is based on (ectopic) ChIP-seq profiling in a single human cell line, and therefore in a very specific chromatin and regulatory context. The authors should discuss in detail how this caveat contextualises their observations, including on the applicability of the ChIP-seq profiles they report to other human cell types or tissues.

We have added the following at the end of the first paragraph of the Discussion:

"…. We also note that the data we collected are in only a single cell line, and therefore in a very specific chromatin and regulatory context. Some observations, especially those that relate to the genome sequence itself (e.g. conservated sites, TE or promoter binding), can be readily extended to other cell types. Others (e.g. specific binding sites) may be cell-type specific."

- The data annotation in some of the supplementary figures can be improved for readability (e.g. left axis on supplementary figure 8 for the bottom heatmaps, which

could be clearly annotated as in supplementary figure 12).

We have added labels to Supplementary Figure 8 as recommended.

Reviewer #6 (Remarks to the Author):

I co-reviewed this manuscript with one of the reviewers who provided the listed reports. This is part of the Nature Communications initiative to facilitate training in peer review and to provide appropriate recognition for Early Career Researchers who co-review manuscripts.
